# Eugenol@Montmorillonite vs. Citral@Montmorillonite Nanohybrids for Gelatin-Based Extruded, Edible, High Oxygen Barrier, Active Packaging Films

**DOI:** 10.3390/polym17111518

**Published:** 2025-05-29

**Authors:** Achilleas Kechagias, Areti A. Leontiou, Yelyzaveta K. Oliinychenko, Alexandros Ch. Stratakos, Konstatninos Zaharioudakis, Charalampos Proestos, Emmanuel P. Giannelis, Nikolaos Chalmpes, Constantinos E. Salmas, Aris E. Giannakas

**Affiliations:** 1 Department of Food Science and Technology, University of Patras, 30100 Agrinio, Greece; up1110842@upatras.gr (A.K.); aleontiu@upatras.gr (A.A.L.); zacharioudakis.k@upatras.gr (K.Z.); 2 School of Applied Sciences, College for Health, Science and Society, University of the West of England, Coldharbour Ln, Bristol BS16 1QY, UK; yelyzaveta2.oliinychenko@live.uwe.ac.uk (Y.K.O.); alexandros.stratakos@uwe.ac.uk (A.C.S.); 3 Laboratory of Food Chemistry, Department of Chemistry, National and Kapodistrian University of Athens Zografou, 15771 Athens, Greece; harpro@chem.uoa.gr; 4 Department of Materials Science and Engineering, Cornell University, Ithaca, NY 14850, USA; epg2@cornell.edu; 5 Department of Material Science and Engineering, University of Ioannina, 45110 Ioannina, Greece

**Keywords:** gelatin, eugenol, citral, montmorillonite, edible packaging, active packaging, high oxygen barrier, minced pork, shelf life

## Abstract

In the context of the circular economy, the valorization of bio-derived waste has become a priority across various production sectors, including food processing and packaging. Gelatin (Gel), a protein which can be recovered from meat industry byproducts, offers a sustainable solution in this regard. In this study, pork-derived gelatin was used to develop novel edible active packaging films, designed for meat products. Glycerol (Gl) was used as a plasticizer. Two types of montmorillonite-based nanohybrids were employed as both reinforcing agents and carriers of antioxidant/antibacterial compounds: eugenol-functionalized montmorillonite (EG@Mt) and citral-functionalized montmorillonite (CT@Mt). The active films were formulated as Gel/Gl/xEG@Mt and Gel/Gl/xCT@Mt, where x = 5, 10, or 15 wt.%. Controlled-release kinetics showed that EG@Mt released up to 95% of its adsorbed eugenol, whereas CT@Mt released up to 55% of its adsorbed citral. The films were evaluated using the DPPH (2,2-diphenyl-1-picrylhydrazyl) assay and tested for antibacterial activity against *Escherichia coli* and *Listeria monocytogenes*. Results demonstrated that the Gel/Gl/xEG@Mt films exhibited superior antioxidant and antibacterial performance compared to the Gel/Gl/xCT@Mt films. All formulations were impermeable to oxygen. Although the incorporation of EG and CT slightly reduced cell viability, values remained above 80%, indicating non-toxicity. In conclusion, the film containing 15 wt.% EG@Mt achieved an oxygen transmission rate of zero, an effective concentration (EC_60_) of 9.9 mg/L to reach 60% antioxidant activity, and reduced *E. coli* and *L. monocytogenes* populations by at least 5.8 log CFU/mL (*p* < 0.05), bringing them below the detection limit. Moreover, it successfully extended the shelf life of fresh minced pork by two days.

## 1. Introduction

Nowadays, packaging plays a vital role in ensuring that products comply with legal requirements and meet consumer expectations in terms of safety, nutritional value, and sensory properties. As a result, researchers are focused on developing packaging solutions that fulfill these standards while aligning with modern sustainability trends [1,2,3,4,5]. Addressing the pressing challenges faced by both industry and society is essential to achieving these goals. One of the major concerns is environmental pollution caused by non-biodegradable plastics, which are commonly used in food packaging. Over the past 70 years, more than 8 billion tons of plastic have been produced, much of which ends up polluting ecosystems. These petroleum-based plastics can take centuries to degrade and pose a threat to wildlife through ingestion. Moreover, the reliance on non-renewable resources for their production raises sustainability issues [6,7].

Another critical issue is the threat to human health posed by hazardous food and packaging additives used for preservation [8,9,10]. Many synthetic additives have been associated with serious health problems, including cancer, obesity, cardiovascular disease, and asthma. Additionally, harmful substances can migrate from packaging into food, exacerbating health risks [11]. Extending food shelf life is also critical to reduce waste and meet the demands of a growing global population [12,13,14,15]. A substantial portion of food is currently lost or wasted before it reaches consumers [13,14].

To promote human and environmental health while supporting bioeconomy and sustainability goals, packaging materials must evolve to: (i) replace petroleum-based plastics with biodegradable, bio-based, and/or edible biopolymers derived from renewable sources such as food waste and biomass [16,17], (ii) substitute synthetic additives with natural, bio-based compounds such as plant extracts and essential oil (EO) derivatives [18,19,20], and (iii) eliminate the direct addition of chemical additives—synthetic or natural—into food by incorporating them into packaging materials, thus enabling the development of active packaging systems with controlled-release capabilities [21,22,23].

Aligned with the first trend, gelatin (Gel) has emerged as a promising biopolymer for packaging materials [24,25,26]. Gelatin is produced by the hydrolysis of collagen under acidic (Type A) or alkaline (Type B) conditions, typically using by-products from the meat industry, such as cartilage, bones, and skin [25]. Its key advantages include biodegradability, low cost, and the ability to form edible films with good oxygen barrier properties. Furthermore, gelatin is well accepted by consumers, as it is already widely used in the food industry for its emulsifying and stabilizing functions [25,26]. However, gelatin films can be brittle and require plasticizers to improve flexibility and mechanical performance [17]. Plasticizers function by embedding themselves between polymer chains, weakening intermolecular interactions and making the material more elastic and durable [17,27]. Glycerol (Gl), a bio-based, biodegradable, and edible plasticizer, is commonly used in gelatin-based films [27]. Its hydrophilic nature allows it to disperse among gelatin macromolecules, increasing flexibility by expanding the intermolecular spacing [25]. Glycerol is widely available and cost-effective, as it is a by-product of biodiesel production [25].

Aligned with the second approach, essential oils (EOs) and their derivatives are gaining attention as natural, low-toxicity additives for food packaging due to their preservative properties [19,20,28,29]. EOs are volatile compounds extracted from plant materials [29]. Eugenol (EG), or 4-allyl-2-methoxyphenol, is a key component of clove oil and is well-suited for active packaging due to its strong antimicrobial, antioxidant, and anti-inflammatory properties [30]. Already used in pharmaceuticals and as a flavoring agent in foods and beverages, eugenol offers additional consumer familiarity and safety [30,31].

Citral (CT), another promising EO, is found in lemongrass and citrus fruits such as lemons, limes, and oranges. Citral is a monoterpene aldehyde consisting of two isomers—neral (cis) and geranial (trans)—collectively known as 3,7-dimethyl-2,6-octadien-1-al [32,33]. Like eugenol, citral exhibits strong antimicrobial activity and has been shown to extend the shelf life of food when used in active packaging systems [34].

On the other hand, the direct incorporation of essential oils (EOs) and their derivatives into packaging materials often leads to a significant loss of their antioxidant and antibacterial activity due to their high volatility. To address this limitation, researchers are increasingly aligning with the third trend: the encapsulation of EOs and their derivatives within nanocarriers such as nanoclays, zeolites, silicas, and activated carbon. These nanohybrids are then incorporated into the packaging matrix, resulting in active packaging films capable of controlled release, thereby preserving and prolonging the functional properties of the EOs throughout the product’s shelf life [35,36,37,38,39,40]. Among the various nanoclays used as nanocarriers for EOs and their derivatives, montmorillonite (Mt) is the most commonly employed due to its low cost and natural abundance [36,41]. The adsorption of essential oils (EOs) and their derivatives onto montmorillonite (Mt) is well established. It is widely recognized that these compounds typically adsorb onto the external surfaces of Mt rather than intercalating into its interlayer spaces [36,42,43,44]. In such non-intercalated EO@nanoclay hybrids, the interlayer regions remain hydrophilic, which reduces compatibility with hydrophobic polymers and biopolymers. Facilitating the intercalation of EOs and their derivatives into the interlayer spaces of nanoclays could significantly enhance the controlled release behavior of EO@nanoclay hybrids. Moreover, it could improve their dispersion and functionality within polymer matrices, particularly at higher loadings.

In this study, we present the development of gelatin type A (Gel) and glycerol (Gl) films enhanced with montmorillonite (Mt)-based nanohybrids functionalized with the essential oils eugenol (EG) and citral (CT). A comprehensive characterization of both the nanohybrids and the resulting active films is also provided. The key innovative aspects of this study, reported here for the first time include: (i) the synthesis of EG@Mt and CT@Mt nanohybrids, their release kinetics, and their physicochemical characterization using X-ray diffraction (XRD), Fourier transform infrared (FTIR) spectroscopy, and scanning electron microscopy (SEM), (ii) the fabrication of novel Gel/Gl/xEG@Mt (where x = 5, 10, and 15 wt.%) and Gel/Gl/xCT@Mt (where x = 5 and 10 wt.%) active packaging films via an extrusion molding-compression method, along with their characterization using XRD, FTIR, and SEM, and (iii) the evaluation of the films’ oxygen barrier properties (showing zero permeability), antioxidant and antibacterial activities, and biocompatibility.

## 2. Materials and Methods

### 2.1. Materials

Gelatin type A with catalog numbers AC611995000 and CAS 9000-70-8 was purchased from Thermos Scientific Chemicals (Thermo Fisher Scientific. 168 Third Avenue. Waltham, MA, USA 02451). Eugenol, 2-Methoxy-4-(2-propenyl) phenol, 4-Allyl-2-methoxyphenol, 4-Allylguaiacol with CAS number: 97-53-0 and 99.98% purity and Citral, Lemonal, 3,7-Dimethyl-2,6-octadienal with CAS number: 5392-40-5 and 99.98% purity were purchased from Sigma-Aldrich (Darmstadt, Germany). Montmorillonite nanoclay with CAS Number: 1318-93-0 30 and CEC 30 meq/100 g was purchased from Sigma-Aüldrich (Darmstadt, Germany). The compound 2,2-diphenyl-1-picrylhydrazyl (DPPH) CAS number: 1898-66-4 was purchased also from Sigma-Aüldrich (Darmstadt, Germany).

### 2.2. EG@Mt and CT@Mt Nanohybrid Synthesis

Both EG@Mt and CT@Mt nanohybrids were prepared via our laboratory method based on vacuum-assisted adsorption process, which was published recently [45]. According to this synthesis protocol, an amount of 2 g of pure Mt was dried under vacuum for 15 min at 100 °C. The free adsorbed water nanoclay was impregnated under stirring with EG and CT via dropwise adsorption. Then, the obtained EG@Mt and CT@Mt nanohybrids were removed from the glass flask and weighted to calculate the adsorbed amount of EG and CT. The weight of the obtained EG@Mt and CT@Mt nanohybrids was found to be 3.1 g for both. This means that the adsorbed amount of both EG and CT in EG@Mt and CT@Mt nanohybrids is 35.6 wt.% each. The prepared EG@Mt and CT@Mt nanohybrids were preserved at 25 °C and 50% RH for further uses.

### 2.3. Gel/Gl/xMt, Gel/Gl/xEG@Mt, and Gel/Gl/xCT@Mt Films Development

A Haake Mini Lab II mini lab extruder mounted with a twin screw, and provided by Thermo Scientific, ANTISEL, S.A., company, located in Athens, Greece, was employed for the Gel/Gl/xMt, Gel/Gl/xEG@Mt, and Gel/Gl/xCT@Mt film development. For each batch, this instrument was rotated for 5 min at 250 rpm, and the synthesis temperature was set at 110 °C. First, 4 g of Gel, 1 g of Gl, and 1.6 g of water were mixed and extruded to take the “blank” Gel/Gl sample film. To obtain Gel/Gl with 5, 10, and 15 wt.% EG@Mt content, 0.347 g, 0.733 g, and 1.160 g of EG@Mt were extruded with 4 g of Gel, 1 g of Gl, and 1.6 g, correspondingly. To obtain Gel/Gl with 5 and 10 wt.% CT@Mt content, 0.347 g and 0.733 g of CT@Mt were extruded with 4 g of Gel, 1 g of Gl, and 1.6 g, correspondingly. For comparison, Gel/Gl/xMt samples were extruded by adding 0.347 g and 0.733 g of dried Mt in 4 g of Gel, 1 g of Gl, and 1.6 g of water to obtain films with 5 and 10 wt.% Mt, correspondingly. In the case of Mt and CT@Mt-based films, the addition of pure Mt and CT@Mt nanohybrid increase the hardness of obtained composites, and we were unable to obtain Gel/Gl/x@Mt and Gel/Gl/xCT@Mt films with 15 wt.% CT@Mt content. The obtained extruded pellets of Gel/Gl/xMt (x = 5, 10 wt.%), Gel/Gl/xEG@Mt (x = 5, 10, 15 wt.%), and Gel/Gl/xCT@Mt (x = 5, 10 wt.%) were pressed for 2 min under 1 tn load using a Specac Atlas™ Series Heated Platens, provided by Specac company, located in Orpinghton, UK. The active films produced by this heat pressing exhibited an average diameter of 10 cm and an average thickness of 0.2 mm, and were preserved under 25 °C temperature and relative humidity of 50% RH for further use. Table 1 presents all material names, amounts, and synthesis conditions.

### 2.4. Physicochemical Characterization of EG@Mt and EG@Mt Nanohybrids and Gel/Gl/xMt, Gel/Gl/xEG@Mt, and Gel/Gl/xCT@Mt Films

The obtained EG@Mt and EG@CT nanohybrids as well as Gel/Gl/xMt (x = 5, 10), Gel/Gl/xEG@Mt (x = 5, 10, 15), and Gel/Gl/xCT@Mt (x = 5, 10) films were characterized with X-Ray diffraction (XRD), Fourier transform infrared spectroscopy (FTIR), and scanning electron microscopy (SEM) analysis. The instrumentation as well as the methodology followed for XRD, FTIR, and SEM analysis are given in the Supplementary Materials.

The EG and CT amounts desorbed from Mt, as well as the EG and CT release rate desorption kinetic experiments were carried out for both EG@Mt and CT@Mt nanohybrids using a moisture analyzer AXIS AS-60 (AXIS Sp. z o.o. ul. Kartuska 375b, 80–125 Gdańsk, Poland), and the methodology is described in the Supplementary Materials.

Briefly, approximately 100 mg of each nanohybrid sample was placed in a moisture analyzer and its weight was recorded over time (in triplicates) at 70, 90, and 110 °C. From the recorded weight data as a function of time (m_t_), the normalized fraction q_t_ = (1 − m_t_/m_0_) was calculated and plotted against time. These plots were then fitted using the well-known pseudo-second-order adsorption–desorption equation [46,47]. For a process of order n = 2, the overall normalized mass balance is given by:

(1) $$\frac{{dq}_{t}}{dt}=k_{2}*{\left(q_{e}-q_{t}\right)}^{2}$$

where k_2_ is the rate constant of the pseudo-second-order kinetic model (s^−1^), qt is the desorbed fraction capacity at time t, and q_e_ = (1 − m_e_/m_0_) represents the maximum desorbed fraction capacity at equilibrium, m_0_ is the initial EOs loading in the nanohybrid, and m_t_ is the EOs amount remaining in the nanohybrid at time t. By integrating Equation (1), the pseudo-second-order kinetic model is obtained:

(2) $$q_{t}=(1-\frac{m_{t}}{m_{0}})=\frac{q_{e}^{2}*k_{2}*t}{q_{e}*k_{2}*t+1}$$

The initial release rate can be determined using Equation (1) by evaluating the expression at t = 0 (i.e., when q_t_ = 0). Accordingly:

(3) $$r_{i}={\left.\frac{{dq}_{t}}{dt}\right|}_{t=0}=k_{2}*q_{e}^{2}$$

From the best-fitted plots, the values of k_2_ and q_e_ values were determined. Using the estimated k_2_ parameter, the term ln(k_2_) was calculated and plotted as a function of (1/T) to determine the desorption energy (Ε^0^_des_) based on the Arrhenius equation and the theoretical framework presented in detail in Refs. [48,49,50]:

(4) $$k_{2}=k_{0}\cdot e^{-\frac{E_{des}^{0}}{R\cdot T}}$$

and its linear transformed type:

(5) $$ln\left(k_{2}\right)=ln\left(k_{0}\right)-\frac{E_{des}^{0}}{R\cdot T}$$

where k_2_ is the rate constant of the pseudo-second-order kinetic model (s^−1^), Ε^0^_des_ is the desorption activation energy, and A is the Arrhenius constant.

### 2.5. Packaging Properties of Gel/Gl/xMt, Gel/Gl/xEG@Mt, and Gel/Gl/xCT@Mt Films

Tensile properties of all Gel/Gl/xMt, Gel/Gl/xEG@Mt, and Gel/Gl/xCT@Mt films were determined according to the ASTM D638 method by following the instrumentation and methodology described in the Supplementary Materials.

The oxygen barrier properties of Gel/Gl/xMt, Gel/Gl/xEG@Mt, and Gel/Gl/xCT@Mt films as well as the pure Gel/Gl film were determined according to ASTM D 3985 at 23 °C and 0% RH using an oxygen permeation analyzer (O.P.A., 8001, Systech Illinois Instruments Co., Johnsburg, IL, USA). For each sample, five to seven separate disk-shaped films, with an average diameter of 11 cm and a thickness ranging from 80 to 150 μm, were carefully placed inside the O.P.A. chamber. The oxygen transmission rate (O.T.R.) was determined by recording the final value of the obtained isotherm curve. Each measurement lasted a minimum of 24 h, and samples were allowed to equilibrate for at least 12 h after reaching the final equilibrium value.

In Vitro Antioxidant Activity, biocapability, and antibacterial activity of all Gel/Gl/xMt, Gel/Gl/xEG@Mt, and Gel/Gl/xCT@Mt films were determined according to the methodology described in detail in the Supplementary Materials.

### 2.6. Packaging Preservation Test of Fresh Minced Pork Wrapped with Gel/Gl/10EG@Mt, Gel/Gl/10CT@Mt Active Films, and Commercial Film

For the packaging preservation test of fresh minced pork, Gel/Gl/15EG@Mt and Gel/Gl/10CT@Mt active films were selected as the optimum to test. Fresh minced pork was offered by the Ayfantis meat processing company. Minced pork in portions of approximately 40–50 g each were aseptically wrapped between two films of Gel/Gl/10EG@Mt and Gel/Gl/10CT@Mt active films that were 10 cm in diameter, and placed inside the Ayfantis company’s commercial wrapping paper, without the inner film. For the control sample, 40–50 g of minced pork was aseptically wrapped with the commercial packaging paper of the Ayfantis company (coated with plasticized PVC). The aseptic wrapping of minced pork was conducted under sterile conditions, including thorough bench cleaning with pure ethanol and the continuous operation of two Bunsen lamps to maintain a sterile environment. For all tested packaging systems, samples for the 2nd, 4th, and 6th day of preservation were prepared and stored under dark refrigerator conditions at 4 ± 1 °C (LG GC-151SA, Weybridge, UK).

The total Viable Count (TVC), pH analysis, colorimetry analysis, and sensory analysis (color, odor, and texture) values of minced pork during the 10 days of storage were determined according to the methodology described in detail in the Supplementary Materials.

### 2.7. Statistical Analysis

k_2_, q_e_, E_0,des_, EC_50_, EC_60_, elastic modulus (E), ultimate strength (σuts), %elongation at break (%ε), biocompatibility, antibacterial, total variable counts (TVC), pH, colorimetry, and sensory analysis (color, odor, texture) mean values as well as their standard deviation were calculated. These properties were also subjected to further statistical analysis using the ANOVA with Tukey’s HSD method to investigate statistical differences/equalities between properties’ mean values. Assuming a significance level of *p* < 0.05, all measurements were conducted using five to seven separate samples of each Gel/Gl/xMt, Gel/Gl/xEG@Mt, and Gel/Gl/xCT@Mt film. Statistical analysis was carried out using SPSS software (v. 28.0, IBM, Armonk, NY, USA).

## 3. Results

### 3.1. Physicochemical Characterization of EG@Mt and CT@Mt Nanohybrids

#### 3.1.1. EG and CT Release Kinetics

**Figure 1:**
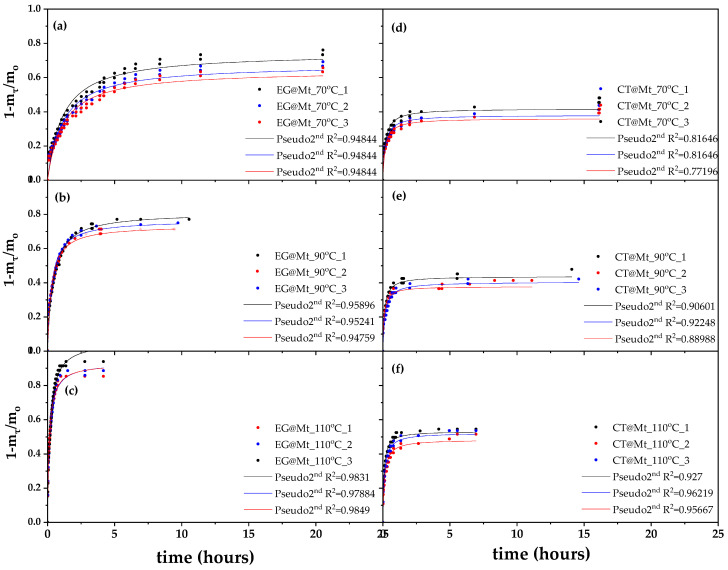
EG and CT desorption isotherm kinetic plots (in triplicates) for EG@Mt (left part (**a**–**c**) plots) and CT@Mt (right part (**d**–**f**) plots) nanohybrids at 70 °C ((**a**,**d**) plots), 90 °C ((**b**,**e**) plots), and 110 °C ((**c**,**f**) plots). Line plots show the simulation plots according to the pseudo-second-order kinetic model.

Figure 1 shows the recorded values of (1 − m_t_/m_0_) as a function of time (t) for EG@Mt and CT@Mt nanohybrids (in triplicate) at 70, 90, and 110 °C.

These plots were fitted with the pseudo-second-order kinetic model to calculate the k_2_ and q_e_ values according to Equation (1), which are listed in Table 1.

As shown in Figure 1, the pseudo-second-order kinetic model provides an excellent fit for all release profiles of eugenol (EG) and carvacrol (CT). Based on the kinetic rate constants (k_2_) summarized in Table 2, EG consistently exhibits lower release rates than CT at all tested temperatures, indicating a slower but more sustained release profile. Additionally, the equilibrium release capacities (q_e_) reveal that higher amounts of EG are released compared to CT across all temperatures. Specifically, EG release reached 69% at 70 °C, 77% at 90 °C, and 94% at 110 °C, whereas CT release was limited to 38%, 41%, and 51% at the same temperatures, respectively.

Considering that the initial adsorbed amount of both EG and CT on Mt was 35.6 wt.%, these results indicate that nearly the entire adsorbed EG content is released from the EG@Mt nanohybrid, whereas only about half of the CT content is released from the CT@Mt nanohybrid. This suggests stronger interactions or retention of CT within the Mt structure.

**Figure 2:**
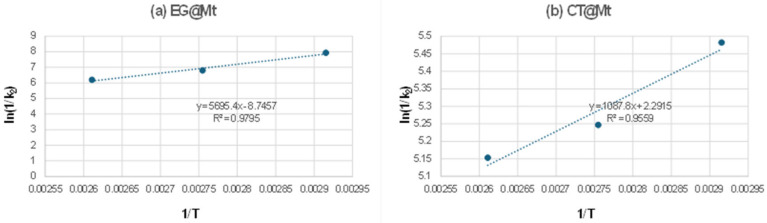
ln(1/k_2_) values as a function of (1/T) plots for (**a**) EG@Mt and (**b**) CT@Mt nanohybrids.

Furthermore, to investigate the temperature dependence of the release kinetics, the natural logarithm of the inverse kinetic rate constant, ln(1/k_2_), was plotted against the reciprocal temperature (1/T) for both EG@Mt and CT@Mt nanohybrids, as shown in Figure 2.

From the linear fitted plots in Figure 2, the calculated slopes were used with Equations (2) and (3) to determine the EG and CT desorption energies (E_0,des_). The values are 11.2 and 2.1 Kcal/mol for EG@Mt and CT@Mt, respectively. The calculated E_0,des_ values are consistent with the results above. These values imply a higher desorption energy of EG molecules than CT molecules, and thus EG release rates are lower than CT release rates.

Overall, both EG@Mt and CT@Mt nanohybrids adsorbed equal amount of EG and CT, while EG@Mt nanohybrid release higher amounts of EG with lower release rates than CT@Mt nanohybrid.

#### 3.1.2. XRD Analysis of EG@Mt and CT@Mt Nanohybrids

**Figure 3:**
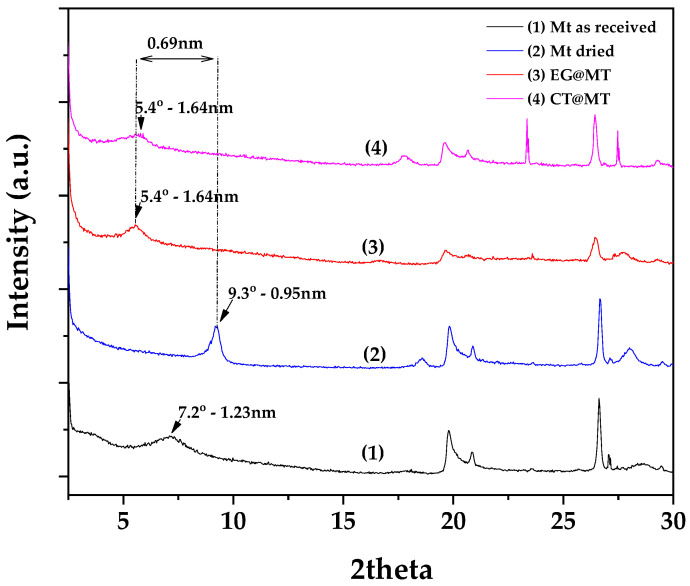
XRD plots of (1) Mt as received, (2) dried Mt, (3) EG@Mt nanohybrid, and (4) CT@Mt nanohybrid.

In Figure 3, the recorded XRD plots of Mt as received, dried Mt, as well as obtained EG@Mt and CT@Mt nanohybrids are shown for comparison. The XRD pattern of the as received Mt shows a d-spacing of 1.23 nm. After the drying process, the XRD pattern of dried Mt shows a d-spacing decreased to 0.93 nm, which corresponds to collapsed layers suggesting that vacuum-assisted drying successfully removes all of the adsorbed water molecules. In both modified EG@Mt and CT@Mt nanohybrids, the characteristic reflection of Mt’s basal space is at 2 theta = 5.4°, corresponding to a 1.64 nm interlayer space. Considering that both EG’s and CT’s molecule size is equal to that of phenol (0.5 nm), the obtained XRD patterns for both EG@Mt and CT@Mt nanohybrids suggest intercalation of both EG and CT molecules inside Mt’s galleries.

#### 3.1.3. FTIR Analysis of EG@Mt and CT@Mt Nanohybrids

**Figure 4:**
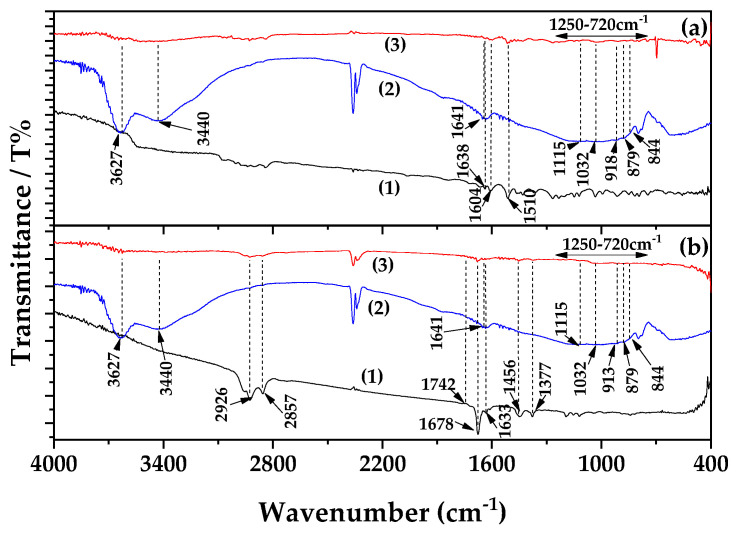
(**a**) FTIR plots of (1) pure EG, (2) pure Mt, and (3) EG@Mt nanohybrid, (**b**) FTIR plots of (1) pure CT, (2) pure Mt, and (3) CT@Mt nanohybrid.

Plot line (1) of Figure 4a corresponds to the FTIR spectrum of EG. In this spectrum, the peaks observed in the 720–1250 cm^−1^ region correspond to C=C vibrations, which are characteristic of EG [51,52]. Additionally, the sharp peaks at 1638, 1604, and 1510 cm^−1^ are attributed to C=C stretching within the aromatic ring of EG [51,52].

Plot line (1) of Figure 4b represents the FTIR spectrum of CT. The distinct peak at 1742 cm^−1^ is likely associated with the ester group and C–C stretching of CT [53,54,55,56]. The peaks at 1370 cm^−1^ and 1463 cm^−1^ correspond to the C–H bending vibrations of CH_3_ and CH_2_ groups, respectively [53,54,55,56]. Peaks at 2857 cm^−1^ and 2924 cm^−1^ are attributed to CH_2_ and CH_3_ stretching vibrations, while a strong absorption band at 1670 cm^−1^ is due to the C=O stretching vibration of CT [53,54,55,56]. Further characteristic peaks appear at 2860 cm^−1^ and 2970 cm^−1^, corresponding to symmetric and asymmetric CH_3_ stretching vibrations. The peak at 1630 cm^−1^ is assigned to C–C stretching, and those at 1450 cm^−1^ and 1380 cm^−1^ are attributed to asymmetric and symmetric bending vibrations of CH_2_ and CH_3_ groups, respectively [53,54,55,56].

Plot line (2) in Figure 4a,b corresponds to the FTIR spectrum of pristine clay. Mt shows a characteristic band at ~3626 cm^−1^, attributed to the stretching vibration of OH groups bonded to Al^3+^ cations [39,54]. The band at ~3442 cm^−1^ is associated with H_2_O stretching vibrations, while the band at ~1641 cm^−1^ is assigned to H_2_O bending. Peaks at ~1113 cm^−1^ and 1031 cm^−1^ are indicative of Si–O stretching vibrations in Mt [55]. Additional bands at 913, 879, and 844 cm^−1^ correspond to OH bending modes, with the ~913 cm^−1^ band representing AlAl–OH bending, the ~879 cm^−1^ band indicating AlFe–OH bending, and the ~844 cm^−1^ band corresponding to FeFe–OH bending.

Plot line (3) in Figure 4a,b corresponds to the FTIR spectra of EG@Mt and CT@Mt nanohybrids, respectively. These spectra combine the characteristic peaks of EG or CT with those of Mt, indicating the successful adsorption of EG and CT molecules onto Mt. The absence of any significant blue or red shifts in the observed peaks suggests that adsorption occurs via physisorption, in agreement with the desorption energy calculations presented earlier.

#### 3.1.4. SEM Images of EG@Mt and CT@Mt Nanohybrids

**Figure 5:**
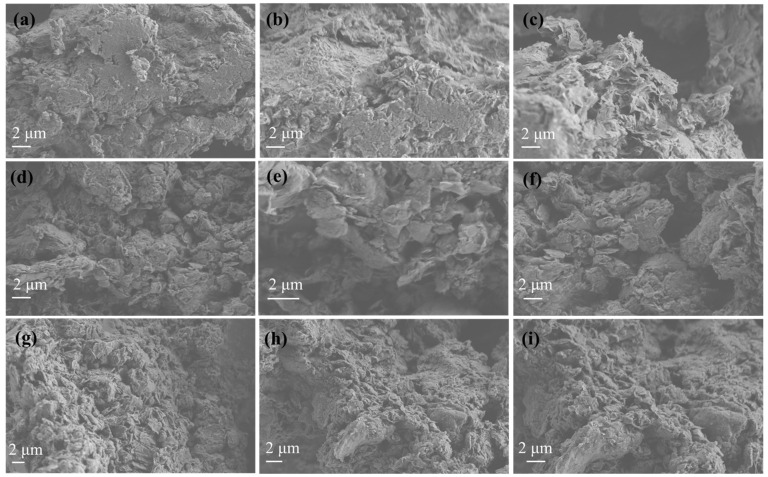
HR-SEM images of (**a**–**c**) pure Mt, (**d**–**f**) EG@Mt nanohybrid, and (**g**–**i**) CT@Mt nanohybrid.

The morphological characteristics of pure Mt, as well as the EG@Mt and CT@Mt nanohybrids, are presented in Figure 5. The pure Mt (images a, b, and c) exhibits a platelet-like morphology with a clearly defined lamellar structure. In contrast, the surfaces of the clay nanoplatelets in the EG@Mt (images d, e, and f) and CT@Mt (images g, h, and i) nanohybrids appear more compact and smoother. This change in surface texture is attributed to the intercalation of EG and CT molecules into the Mt structure. The intercalation of both EG and CT leads to a more uniform appearance of the nanohybrids, with fewer visible pores. These SEM observations are consistent with the XRD results discussed earlier, supporting the successful intercalation of EG and CT molecules within the Mt interlayer spaces.

### 3.2. Physicochemical Characterization of Gel/Gl/xMt, Gel/Gl/xEG@Mt, and Gel/Gl/xCT@Mt Films

#### 3.2.1. XRD Analysis of Gel/Gl/xMt, Gel/Gl/xEG@Mt, and Gel/Gl/xCT@Mt Films

**Figure 6:**
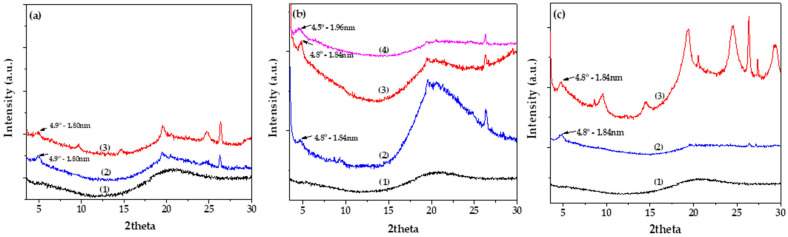
XRD patterns in the 2θ range of 2° to 30° for: (**a**) (1) Gel/Gl film, (2) Gel/Gl/5Mt film, and (3) Gel/Gl/10Mt film; (**b**) (1) Gel/Gl film, (2) Gel/Gl/5EG@Mt film, (3) Gel/Gl/10EG@Mt film, and (4) Gel/Gl/15EG@Mt film; (**c**) (1) Gel/Gl film, (2) Gel/Gl/5CT@Mt film, and (3) Gel/Gl/10CT@Mt film.

Figure 6 presents the XRD patterns of Gel/Gl/xMt films (Figure 6a), Gel/Gl/xEG@Mt active films (Figure 6b), and Gel/Gl/xCT@Mt active films (Figure 6c). In the XRD pattern of the pure Gel/Gl film (see plot line (1) in Figure 6a–c), a broad peak around 20° 2θ is observed, which corresponds to the amorphous phase of gelatin plasticized with glycerol [57,58,59]. In the Gel/Gl/xMt films (plot lines (2) and (3) in Figure 6a), the Gel/Gl/xEG@Mt films (plot lines (2), (3), and (4) in Figure 6b), and the Gel/Gl/xCT@Mt films (plot lines (2) and (3) in Figure 6c), additional peaks appear at approximately 4.5°, 4.8°, and 4.9° 2θ. These peaks indicate the successful intercalation of Gel/Gl chains into the interlayer spaces of Mt, EG@Mt, and CT@Mt, respectively. Thus, the XRD results confirm the formation of intercalated nanocomposite structures in all cases, demonstrating the effective incorporation of Mt-based nanohybrids into the Gel/Gl film matrix. Moreover, as shown in Figure 6b, a prominent amorphous phase peak appears at approximately 20^o^ in the XRD pattern of the Gel/Gl/5EG@Mt film. This peak gradually decreases in intensity as the EG@Mt content increases to 10 and 15 @wt, indicating a reduction in the amorphous phase with higher EG@Mt loading in the Gel/Gl/xEG@Mt films.

#### 3.2.2. FTIR Analysis of Gel/Gl/xMt, Gel/Gl/xEG@Mt, and Gel/Gl/xCT@Mt Films

**Figure 7:**
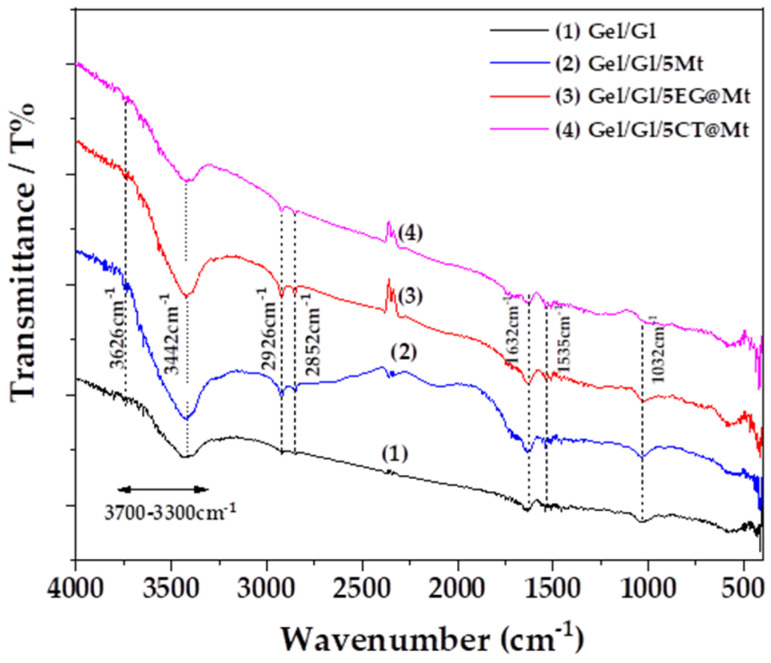
FTIR plots of (1) Gel/Gl, (2) Gel/Gl/5Mt, (3) Gel/Gl/5EG@Mt, and (4) Gel/l/5CT@Mt films.

Figure 7 presents representative FTIR spectra of Gel/Gl, Gel/Gl/5Mt, Gel/Gl/5EG@Mt, and Gel/Gl/5CT@Mt films. All films exhibit the characteristic absorption bands of gelatin (Gel). The broad band observed between 3500 and 3200 cm^−1^ is attributed to the stretching vibrations of O–H and N–H groups. Peaks at 2926 cm^−1^ and 2852 cm^−1^ correspond to C–H stretching vibrations. Additionally, characteristic amide peaks are observed at 1632 cm^−1^ (amide I, C=O stretching), 1535 cm^−1^ (amide II, N–H bending), and 1238 cm^−1^ (amide III, C–N stretching) [60,61,62,63]. All film samples also display a peak at 1032 cm^−1^, which is assigned to the O–H stretching of glycerol (Gl), confirming its role as a plasticizer.

The presence of montmorillonite (Mt) nanoclay in the Gel/Gl/5Mt, Gel/Gl/5EG@Mt, and Gel/Gl/5CT@Mt films is confirmed by a band around 3626 cm^−1^, attributed to the stretching vibrations of O–H groups bonded to Al^3+^ cations in Mt, and a characteristic band near 3442 cm^−1^ corresponding to H_2_O stretching vibrations [64].

Notably, no distinct peaks corresponding to EG or CT are observed in the FTIR spectra of the Gel/Gl/5EG@Mt and Gel/Gl/5CT@Mt films, suggesting that these molecules are successfully encapsulated within the Gel/Gl matrix and/or the Mt interlayers.

#### 3.2.3. HR-SEM Studies of Gel/Gl/xMt, Gel/Gl/xEG@Mt, and Gel/Gl/xCT@Mt Films

**Figure 8:**
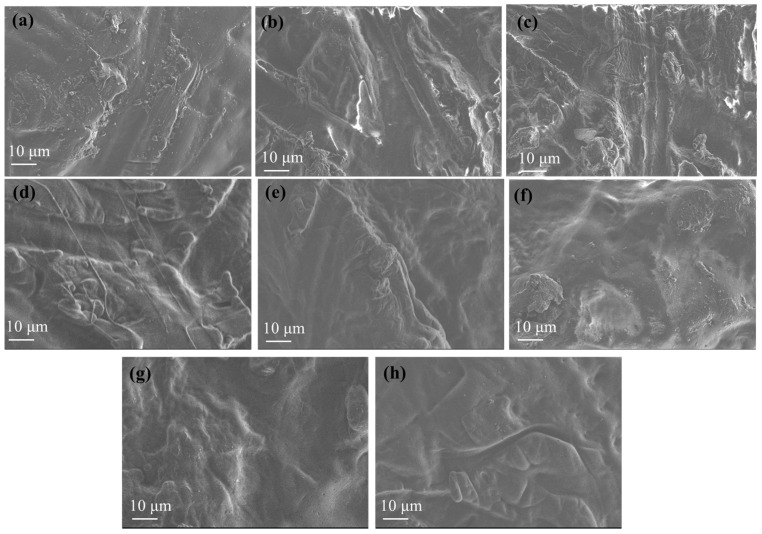
HR-SEM images of (**a**) pure Gel/Gl film, (**b**) Gel/Gl/5Mt film, (**c**) Gel/Gl/10Mt film, (**d**) Gel/Gl/5EG@Mt active film, (**e**) Gel/Gl/10EG@Mt active film, (**f**) Gel/Gl/15EG@Mt active film, (**g**) Gel/Gl/5CT@Mt active film, and (**h**) Gel/Gl/10CT@Mt active film.

Figure 8 presents high-resolution SEM (HR-SEM) images of the pure Gel/Gl film as well as the Gel/Gl/xMt, Gel/Gl/xEG@Mt, and Gel/Gl/xCT@Mt films. The pure Gel/Gl film (Figure 8a) exhibits a semi-rough surface morphology. With increasing Mt content, the Gel/Gl/xMt films show progressively greater surface roughness compared to the pure Gel/Gl film (Figure 8b,c). This increase in roughness is likely due to the aggregation of Mt particles within the Gel/Gl matrix as Mt loading increases [65,66,67,68]. Additionally, the characteristic lamellar structure of the clay is visible in the Gel/Gl/xMt films.

In contrast, both Gel/Gl/xEG@Mt (Figure 8d–f) and Gel/Gl/xCT@Mt (Figure 8g,h) films display a more compact and uniform morphology. This suggests more effective integration of the montmorillonite when EG and CT molecules are intercalated within the clay structure.

### 3.3. Tensile Properties of Gel/Gl/xMt, Gel/Gl/xEG@Mt, and Gel/Gl/xCT@Mt Films

**Figure 9:**
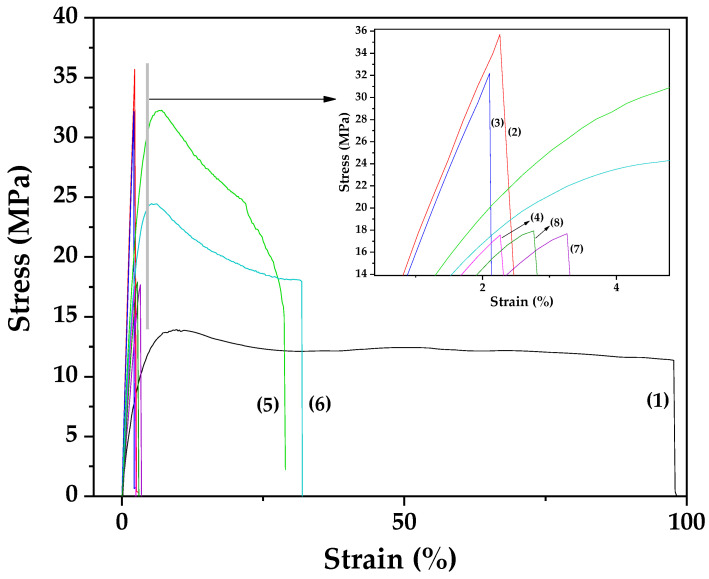
Stress–strain curves for (1) Gel/Gl, (2) Gel/Gl/5Mt, (3) Gel/Gl/10Mt, (4) Gel/Gl/5EG@Mt, (5) Gel/Gl/10EG@Mt, (6) Gel/Gl/15EG@Mt, (7) Gel/Gl/5CT@Mt, and (8) Gel/Gl/10CT@Mt film.

Figure 9 shows the stress–strain curves for all Gel/Gl/xMt, Gel/Gl/xEG@Mt, and Gel/Gl/xCT@Mt films as well as for pure Gel/Gl film.

From the stress–strain curves presented in Figure 9, the mean values of the elastic modulus (E, MPa), ultimate tensile strength (σ_uts_, MPa), and elongation at break (% strain) were calculated and are summarized in Table 3 for comparison.

As shown in Figure 7 and summarized in Table 3, the incorporation of pure Mt, as well as EG@Mt and CT@Mt nanohybrids, into the Gel/Gl matrix increases the ultimate tensile strength while reducing the ductility of the resulting nanocomposite films. Notably, the addition of pure Mt and CT@Mt significantly reduces the ductility of the Gel/Gl/xMt and Gel/Gl/xCT@Mt films, respectively. In contrast, the inclusion of the EG@Mt nanohybrid leads to a comparatively smaller reduction in ductility. Overall, the Gel/Gl/xEG@Mt films demonstrate enhanced mechanical strength while maintaining a more favorable level of flexibility, making them mechanically superior among the tested compositions.

### 3.4. Oxygen Barrier Properties of Gel/Gl/xMt, Gel/Gl/xEG@Mt, and Gel/Gl/xCT@Mt Films

Table 4 lists the observed oxygen transmission rate (OTR) values of all tested Gel/Gl/xMt, Gel/Gl/xEG@Mt, and Gel/Gl/xCT@Mt films for comparison. As shown in Table 4, all films exhibited zero oxygen transmission rate (OTR) and oxygen permeability (PeO_2_) values, indicating that they are completely impermeable to oxygen. Gel is a protein, and protein-based films are well-known for their excellent oxygen barrier properties, making them promising candidates for packaging applications [68,69,70]. It is known that the oxygen permeability of extruded gelatin-based films decreases as the glycerol content approaches 20 wt.% [71]. Additionally, increasing the extrusion temperature above 100 °C and employing compression molding processes are reported to further enhance the oxygen barrier properties of gelatin-based films [72,73]. The compression molding process is also recognized for promoting intercalated structures in polymer or biopolymer/clay nanocomposite materials, which are known to enhance barrier properties [74,75]. Therefore, the zero oxygen permeability values observed in our films support the effectiveness of using a low Gl wt.% content, an extrusion temperature above 100 °C, and the application of compression molding in the fabrication process. It should also be emphasized that to the best of our knowledge, zero oxygen barrier value gelatin-based films are reported here for the first time. Furthermore, the incorporation of pure Mt, EG@Mt, or CT@Mt nanohybrids maintains the observed oxygen permeability at zero.

### 3.5. Antioxidant Activity of Gel/Gl/xEG@Mt and Gel/Gl/xCT@Mt Active Films

In Table 4, calculated EC_50_ and EC_60_ mean values for both Gel/Gl/xEG@Mt and Gel/Gl/xCT@Mt active films are listed for comparison. The data and the obtained curves used for the calculation of both EC_50_ and EC_60_ mean values are listed in Table S1 and Figure S1 in the Supplementary Materials. From the listed EC_50_ and EC_60_ values of active films in Table 4, it is concluded that: (i) EG-based active films exhibited much higher antioxidant activity than CT-based active films, and (ii) the antioxidant activity of both Gel/Gl/xEG@Mt and Gel/Gl/xCT@Mt active films increased by increasing the EG and CT wt.% content, correspondingly.

### 3.6. In Vitro Biocompatibility Assessment of Gel/Gl/xEG@Mt and Gel/Gl/xCT@Mt Films

**Figure 10:**
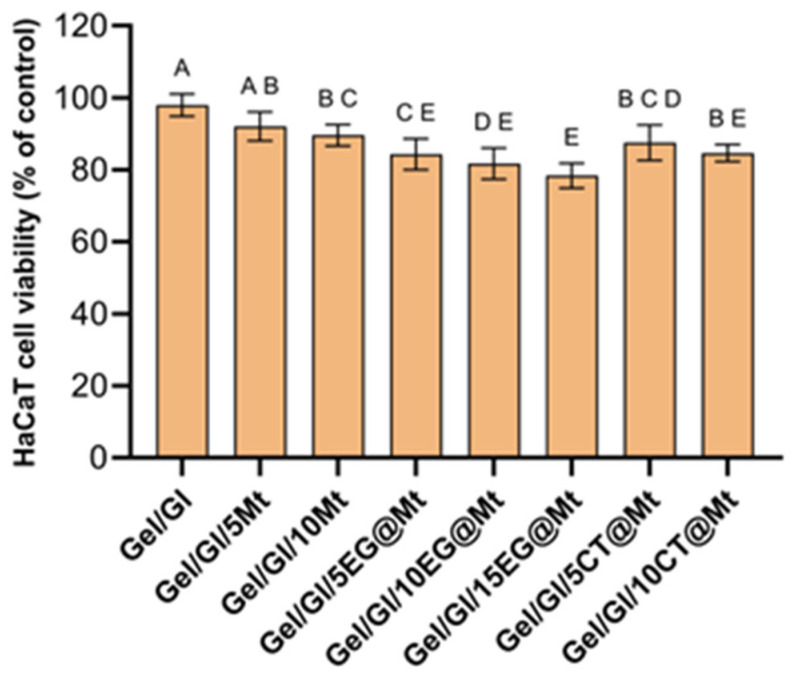
HaCaT skin cell viability in direct contact with films for 24 h. Different letters (A, B, C, D and E) indicate statistically significant differences between the different groups (*p* < 0.05). Error bars represent the standard deviation. See also Table S3 in the Supplementary Materials.

All produced films were tested for their biocompatibility with HaCaT skin cells (Figure 10). The results showed that Mt exhibited acceptable biocompatibility at both concentrations tested. Refined Mt has been shown to be well-tolerated up to 1000 µg/mL in HaCaT cells, which aligns with our findings [76]. The incorporation of EG and CT slightly reduced cell viability; however, in all cases, viability remained high (>80%), which is promising. Only Gel/GL15EG@Mt, containing the highest concentration of eugenol, showed a slightly lower viability at 78.39%. Moreover, no significant differences (*p* > 0.05) were observed between EG- and CT-containing films at the same concentrations, indicating that both antimicrobials are equally well tolerated. To the best of our knowledge, no previous studies have examined the biocompatibility of EG@Mt and CT@Mt nanohybrids with skin cells. While EO components are generally considered safe, their cytotoxicity can vary depending on concentration and formulation. Studies have shown that encapsulation strategies, such as using nanocarriers or polymeric matrices, can mitigate the potential cytotoxic effects of active compounds like EG, enhancing their compatibility with skin cells [77,78].

### 3.7. Antibacterial Activity of Gel/Gl/xEG@Mt and Gel/Gl/xCT@Mt Films

**Figure 11:**
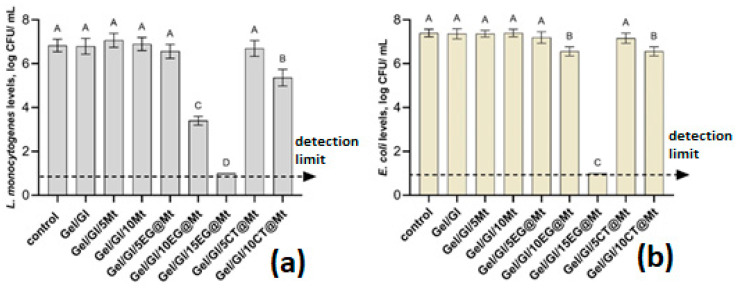
Mean populations of *Listeria monocytogenes* (**a**) and *Escherichia coli* (**b**) in Gel/Gl/xMt, Gel/Gl/xEG@Mt, and Gel/Gl/xCT@Mt films. Data are presented as log_10_ transformations. Different letters (A, B, C, and D) indicate statistically significant differences between the groups (*p* < 0.05). Error bars represent the standard deviation. The detection limit for each bacterial population was 1.0 log CFU/mL. See also Table S4 in the Supplementary Materials.

The antimicrobial efficacy of the films was assessed against *Listeria monocytogenes* (Figure 11a) and *Escherichia coli* (Figure 11b). The control (inoculated media without film) and the Gel/Gl film both supported an average *L. monocytogenes* population of 6.8 log CFU/mL, indicating that the base film had no antibacterial effect. Similarly, films containing only montmorillonite (Gel/Gl/5Mt and Gel/Gl/10Mt) showed no antimicrobial activity, confirming that Mt alone lacks antibacterial properties.

In contrast, EG@Mt-containing films exhibited strong antibacterial activity. Gel/Gl/10EG@Mt reduced *L. monocytogenes* counts by 3.5 log CFU/mL (*p* < 0.05), while Gel/Gl/15EG@Mt achieved the highest inhibition, reducing bacterial levels by at least 5.8 log CFU/mL, bringing them below the detection limit. A clear dose-dependent response was observed, as higher EG loadings (0.35 g, 0.73 g, and 1.16 g) resulted in greater microbial suppression.

CT@Mt-based films demonstrated weaker activity. Gel/Gl/5CT@Mt did not significantly affect *L. monocytogenes* populations, while Gel/Gl/10CT@Mt achieved a modest reduction of 1.4 log CFU/mL (*p* < 0.05). This suggests that increasing CT content from 0.35 g to 0.73 g notably enhances the film’s antibacterial performance.

Similarly to *L. monocytogenes*, no significant difference was observed between the control group (inoculum without any film) and the Gel/Gl film, with bacterial populations reaching 7.3–7.4 log CFU/mL (Figure 11b). In agreement with our study, Mokhtar et al. (2023) reported that montmorillonite facilitates the storage and release of active compounds, but has no antibacterial effect as a layered material [79].

EG@Mt-based films demonstrated a dose-dependent reduction, although the effect was less pronounced for *L. monocytogenes* compared to *E. coli*. Gel/Gl/10EG@Mt reduced *E. coli* populations by 0.8 log CFU/mL (*p* < 0.05), whereas Gel/Gl/15EG@Mt showed the highest antimicrobial activity, reducing bacterial counts by at least 6.4 log CFU/mL and bringing them below the detection limit. CT@Mt-based films were less effective than EG@Mt-functionalized films. Only Gel/Gl/10CT@Mt significantly reduced *E. coli* populations by 0.8 log CFU/mL (*p* < 0.05), while Gel/Gl/5CT@Mt showed no significant antimicrobial effect.

These results confirm that EG@Mt-based films exhibit strong antimicrobial activity in a concentration-dependent manner, with Gel/Gl/15EG@Mt reducing both *L. monocytogenes* and *E. coli levels* below the detection limit. This antimicrobial effect aligns with EG’s known mechanism of action, which involves disrupting membrane permeability, causing intracellular leakage, and inhibiting cell enzymes, leading to reactive oxygen species accumulation and oxidative stress-induced damage [80,81].

CT@Mt-based films demonstrated antimicrobial activity only at higher concentrations (0.73 g-wt.%) and remained less effective than EG-based formulations. The lower efficacy of CT can be attributed to its lower antibacterial activity, as evidenced by its higher minimal bactericidal concentration (MBC) and minimum inhibitory concentration (MIC) compared to eugenol against *E. coli* serotypes (0.71 and 1.26 mg/mL for *E. coli* STEC O26) and MICs against *A. niger* (0.06 and 0.17 mg/mL, respectively). CT’s antibacterial mechanism involves disrupting bacterial membranes and cell walls, altering mitochondrial morphology, causing adenosine triphosphate (ATP) leakage, and inhibiting metabolic pathways, including the tricarboxylic acid (TCA) cycle [82,83].

Overall, *L. monocytogenes* was more susceptible than *E. coli* to both EG- and CT-functionalized films (Figure 11a,b). While the most effective formulation (Gel/Gl/15EG@Mt) completely inhibited both pathogens, at lower concentrations (10EG@Mt), *L. monocytogenes* showed a 3.5 log CFU/mL reduction, whereas *E. coli* showed only a 0.8 log CFU/mL reduction. This suggests that *E. coli* demonstrates greater resistance to EG compared to *L. monocytogenes*. A similar trend was observed for CT-functionalized films, where the highest reduction in *L. monocytogenes* was 1.4 log CFU/mL, while *E. coli* was reduced by 0.8 log CFU/mL. Moreover, this difference in susceptibility aligns with Burt’s (2004) review, which indicates that essential oils (EOs) and their components are generally more effective against Gram-positive bacteria than Gram-negative bacteria [84]. Future studies could focus on combining CT and EG in a single formulation to investigate their potential antibacterial synergistic effects against pathogens.

### 3.8. Packaging Test in Preservation of Fresh Minced Pork

**Figure 12:**
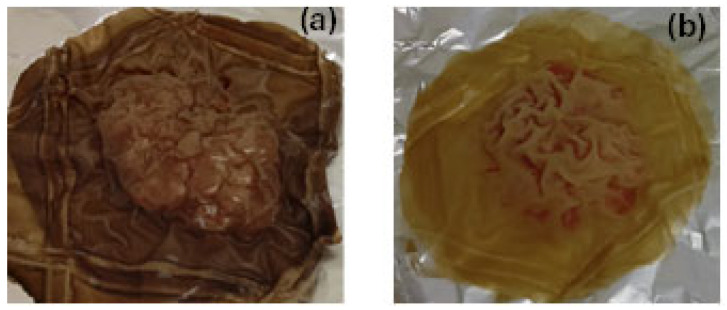
Minced pork wrapped in (**a**) Gel/Gl/15EG@Mt and (**b**) Gel/Gl/10CT@Mt active films after six days of storage at 4 ± 1 °C.

Portions of minced pork wrapped in Gel/Gl/15EG@Mt and Gel/Gl/10CT@Mt active films after six days of storage are shown in Figure 12.

The total viable count of bacteria (TVC), expressed as organisms/g on fresh meat or a meat product, is an important and characteristic factor in its shelf life [85,86,87,88]. The mean calculated TVC values for all packaged minced pork samples (control, Gel/Gl/10CT@Mt, and Gel/Gl/15EG@Mt) are listed in Table 5 for comparison. As it is observed in Table 5, minced pork wrapped with Gel/Gl/15EG@Mt exhibited the lowest TVC values during the six days of storage. Minced pork wrapped with Gel/Gl/10CT@Mt exhibited lower TVC values than the control sample too, but higher than the Gel/Gl/15EG@Mt sample. More specifically, recorded TVC values for minced pork wrapped with Gel/Gl/15EG@Mt are 1 CFU/g lower than the minced pork wrapped with the commercial packaging paper (control sample) during the six days of storage. Both control and Gel/Gl/10CT@Mt on the 4th day of storage exhibited TVC value higher than the 6 log CFU/g limit of acceptance [85,86,87]. To conclude, it could be stated that both Gel/Gl/15EG@Mt and Gel/Gl/10CT@Mt active films succeed to slow down the increase of TVC values of minced pork during the six days of storage, while Gel/Gl/15EG@Mt extends minced pork’s shelf life by approximately 2 days.

TVC results are in accordance with pH and colorimetry values presented in Table 6 and Table 7, respectively, for minced pork wrapped with commercial package (control sample), Gel/Gl/15EG@Mt, and Gel/Gl/10CT@Mt active films.

As shown in Table 6, both **Gel/Gl/15EG@Mt** and **Gel/Gl/10CT@Mt** active films effectively regulated the pH values of minced pork throughout the six-day storage period, in comparison to the control sample. Among the two, the **Gel/Gl/15EG@Mt** film demonstrated superior performance in maintaining lower and more stable pH values, indicating enhanced preservation capacity and reduced microbial activity over time.

The results of the Lab* colorimetry analysis demonstrated that both the type of film and the storage duration significantly influenced the color parameters ΔL, Δa, Δb, and the total color difference (ΔE). Tukey’s HSD post hoc tests confirmed that the lowest ΔE values across the storage days were statistically significant at the 5% level, indicating minimal discoloration. On day 6, the Gel/Gl/15EG@Mt film exhibited the lowest ΔE values (group A), followed by the Gel/Gl/10CT@Mt film (group B), and finally the control sample (group C). These results suggest that the incorporation of active nanohybrids reduced overall color changes in the packaged minced pork. Within each treatment group, the ΔE values increased significantly over time (subgroups a to d), with the control showing the most pronounced discoloration. Trends in Δa and Δb values further confirmed progressive changes in redness and yellowness during storage. Notably, the EG-based film was the most effective in preserving color stability, which is a critical factor for product quality and consumer acceptance.

Complementing these findings, sensory analysis results—covering color, odor, and texture—are summarized in Table 8 for all tested samples.

As shown in Table 8, both Gel/Gl/10CT@Mt and Gel/Gl/15EG@Mt active films effectively preserved the sensory characteristics of fresh minced pork over six days of refrigerated storage, outperforming the control sample. Notably, the Gel/Gl/15EG@Mt active film demonstrated the highest preservation of sensory attributes, which is consistent with the previously discussed results for total viable counts (TVC), pH, and colorimetric (L*a*b*) analyses.

## 4. Discussion

In this study, we successfully developed and characterized novel EG@Mt and CT@Mt nanohybrids and incorporated them into a Gel/Gl matrix via extrusion-compression molding to create innovative high-barrier active films, namely, Gel/Gl/xEG@Mt and Gel/Gl/xCT@Mt. Release kinetics showed that both EG and CT adsorb onto Mt at approximately 45% wt. Nearly 98% of the adsorbed EG was released from EG@Mt, whereas only about half of the adsorbed CT was released from CT@Mt. Calculated desorption energies suggest that both EG and CT are physisorbed onto Mt, with EG having a higher desorption energy, resulting in a slower release rate compared to CT.

XRD and SEM analyses confirmed the successful intercalation of both EG and CT molecules within the Mt interlayer spaces, while FTIR spectra confirmed their adsorption onto the Mt platelets. Although EG intercalation and release kinetics have been previously reported, this study presents, for the first time, similar detailed results for CT intercalation in Mt. Additionally, the development of Gel/Gl/xEG@Mt and Gel/Gl/xCT@Mt films via extrusion-compression molding is also newly reported.

Films containing 5, 10, and 15 wt.% EG@Mt and up to 10 wt.% CT@Mt were successfully fabricated. XRD confirmed the formation of intercalated nanocomposites across all Gel/Gl/xMt, Gel/Gl/xEG@Mt, and Gel/Gl/xCT@Mt films. FTIR spectra showed Mt presence, but no detectable EG or CT peaks in Gel/Gl/xEG@Mt and Gel/Gl/xCT@Mt films, indicating effective encapsulation. SEM images revealed a more compact morphology for films containing EG@Mt and CT@Mt, suggesting better integration when EOs were intercalated.

Tensile testing indicated that adding pure Mt, EG@Mt, and CT@Mt nanohybrids increased the ultimate tensile strength, but reduced ductility. Notably, Gel/Gl/xEG@Mt films exhibited higher strength and maintained greater ductility than Gel/Gl/xCT@Mt films. Impressively, all active films showed zero oxygen permeability, a first report for gelatin-based films, validating the chosen glycerol content, extrusion temperature, and molding process.

Antioxidant activity assessed by DPPH assay revealed that Gel/Gl/xEG@Mt films significantly outperformed Gel/Gl/xCT@Mt films, corroborated by EC50 and EC60 values. Similarly, Gel/Gl/xEG@Mt films exhibited stronger antibacterial activity against *Listeria monocytogenes* and *Escherichia coli*. These findings align with the higher EG release capacity compared to CT, validating the release kinetics results.

Both EG and CT slightly reduced cell viability in the films, but viability remained above 80%, indicating promising biocompatibility. To our knowledge, this is the first study examining the biocompatibility of such gelatin-based active films with skin cells.

Finally, Gel/Gl/15EG@Mt and Gel/Gl/10CT@Mt films were tested as active packaging for fresh minced pork. Total viable count (TVC) analysis showed that both films delayed microbial growth over six days compared to commercial packaging paper. These results were consistent with pH, color (Lab), and sensory analyses. Overall, the Gel/Gl/15EG@Mt film demonstrated potential as an active packaging material capable of extending the shelf life of minced pork by two days.

It is well established that Gel films, especially when incorporated with active ingredients like antioxidants and antimicrobials, can significantly extend the shelf life of pork meat by reducing microbial growth, preventing oxidation, and maintaining color and overall quality. This is achieved by creating a barrier that limits water loss, oxygen penetration, and the growth of spoilage microorganisms [88,89,90,91].

Furthermore, it is important to note that edible films used for food packaging must not be exposed to external contaminants, as this would compromise their edibility. Therefore, a promising future direction of this work involves the development of a bi-layer food packaging system. This system would consist of an inner layer made from the edible, active Gel/Gl/15EG@Mt film and an outer layer composed of LDPE. The combination of these materials would shield the gelatin-based film from external contamination while enhancing the overall barrier performance, combining the zero oxygen permeability attribute of the Gel-based film with the excellent water barrier properties of LDPE.

## 5. Conclusions

In conclusion, films containing EG@Mt nanohybrids demonstrated superior performance compared to those with CT@Mt or pure Mt. This advantage arises from several factors, notably, the controlled release of essential oils, where EG@Mt exhibited a slower yet higher overall release. This behavior translated into enhanced antioxidant and antibacterial activities while maintaining negligible toxicity. Characterization techniques such as XRD, FTIR, and HR-SEM confirmed the effective incorporation of EOs@Mt nanohybrids within the Gel/Glycerol matrix. Combined with the mechanical properties and zero oxygen permeability, these findings highlight Gel/Gl/xEG@Mt films as strong candidates for scale-up and development into commercial active food packaging materials. Among them, the Gel/Gl/15EG@Mt film stands out as the most promising for extending the shelf life of minced pork.

## Figures and Tables

**Table 1:** Samples’ coding, Gel, Gl, water, Mt, EG@Mt, and CT@Mt materials’ amounts, and extruding process conditions (i.e., temperature, rotation speed, and time) for the development of all LDPE/xEG@Lap and LDPE/xEG@Mt active films.

Sample Name	Gel (g)	Gl (g)	H_2_O (g)	Mt (g)	EG@Mt (g)	CT@Mt (g-wt.%)
Gel/Gl	4	1	1.6	-	-	-
Gel/Gl/5Mt	4	1	1.6	0.347	-	-
Gel/Gl/10Mt	4	1	1.6	0.733	-	-
Gel/Gl/5EG@Mt	4	1	1.6	-	0.347	-
Gel/Gl/10EG@Mt	4	1	1.6	-	0.733	-
Gel/Gl/15EG@Mt	4	1	1.6	-	1.160	-
Gel/Gl/5CT@Mt	4	1	1.6	-	-	0.347
Gel/Gl/10CT@Mt	4	1	1.6	-	-	0.733

**Table 2:** Calculated k_2_, q_e_, and mean values from EG and CT desorption kinetic plots for both EG@Mt and CT@Mt nanohybrids.

	EG@Mt			CT@Mt		
T (°C)	k_2_ (×10^−4^)	q_e_ (%)	R^2^	k_2_ (×10^−4^)	q_e_ (%)	R^2^
70 °C	3.61 ± 0.265	68.87 ± 5.174	0.948 ± 0.0001	40.87 ± 3.035	38.70 ± 2.907	0.802 ± 0.0257
90 °C	11.15 ± 2.420	77.16 ± 3.820	0.953 ± 0.0057	57.87 ± 18.516	40.72 ± 2.958	0.906 ± 0.0163
110 °C	19.80 ± 4.386	97.26 ± 7.350	0.982 ± 0.0031	52.50 ± 9.193	51.38 ± 2.602	0.949 ± 0.0189

**Table 3:** Calculated elastic modulus E (MPa), ultimate strength σ_uts_ (MPa), and % elongation at break mean values for all Gel/Gl/xMt, Gel/Gl/xEG@Mt, and Gel/Gl/xCT@Mt films.

Sample Code Name	Elastic Modulus	σ_uts_ (MPa)	Elongation (%ε)
Gel/Gl	417.15 ± 38.567 ^a^	14.73 ± 1.616 ^a^	70.94 ± 13.149 ^a^
Gel/Gl/5Mt	2161.43 ± 386.371 ^b^	36.17 ± 3.403 ^b^	2.48 ± 0.972 ^b,d^
Gel/Gl/10Mt	1897 ± 320.391 ^b^	31.50 ± 3.724 ^b,c^	2.24 ± 0.741 ^b,d^
Gel/Gl/5EG@Mt	968.57 ± 16.946 ^c,d^	18.36 ± 2.146 ^a^	2.38 ± 0.225 ^b,d^
Gel/Gl/10EG@Mt	1030.40 ± 113.342 ^d^	32.28 ± 2.936 ^b^	28.73 ± 12.14 ^c^
Gel/Gl/15EG@Mt	1065.00 ± 48.280 ^d^	25.90 ± 2.030 ^c,d^	31.70 ± 10.551 ^c^
Gel/Gl/5CT@Mt	690.13 ± 89.073 ^a,d^	20.69 ± 0.964 ^a,d^	3.60 ± 0.508 ^d^
Gel/Gl/10CT@Mt	870.77 ± 126.432 ^a,d^	20.45 ± 2.509 ^a,d^	2.75 ± 0.254 ^d^

Different letters in each column indicate statistically significant differences at the confidence level *p* < 0.05. See also Table S1 in the Supplementary Materials.

**Table 4:** Oxygen transmission rate (OTR) mean values of all tested films.

	Thickness (mm)	OTR (mL·m^−2^·day^−1^)	EC_60_ (mg/L)	EC_50_ (mg/L)
Gel/Gl	0.08 ± 0.01	0	-	-
Gel/Gl/5Mt	0.12 ± 0.04	0	-	-
Gel/Gl/10Mt	0.15 ± 0.01	0	-	-
Gel/Gl/5EG@Mt	0.13 ± 0.01	0	11.5 ± 0.60	8.37 ± 0.37
Gel/Gl/10EG@Mt	0.14 ± 0.02	0	9.86 ± 0.40	6.80 ± 0.82
Gel/Gl/15EG@Mt	0.11 ± 0.01	0	9.78 ± 0.47	1.67 ± 0.99
Gel/Gl/5CT@Mt	0.12 ± 0.01	0	406.46 ± 17.63	336.69 ± 14.74
Gel/Gl/10CT@Mt	0.13 ± 0.01	0	329.45 ± 42.63	289.52 ± 8.87

**Table 5:** TVC values of pork fillets wrapped with the control, Gel/Gl/10CT@Mt, and Gel/Gl/15EG@Mt films during six days of storage.

Sample Code	log CFU/g			
	Day 0	Day 2	Day 4	Day 6
Control	4.24 ± 0.20 ^a^	5.64 ± 0.11 ^a^	6.89 ± 0.07 ^a^	8.14 ± 0.13 ^a^
Gel/Gl/10CT@Mt	4.24 ± 0.20 ^a^	5.75 ± 0.12 ^a,b^	6.12 ± 0.08 ^c^	7.76 ± 0.07 ^b^
Gel/Gl/15EG@Mt	4.24 ± 0.20 ^a^	4.81 ± 0.04 ^b^	5.88 ± 0.06	7.25 ± 0.03 ^c^

Different letters in each column indicate statistically significant differences at the confidence level *p* < 0.05. See also Table S5 in the Supplementary Materials.

**Table 6:** Calculated mean pH values of minced pork wrapped with commercial package (control sample), Gel/Gl/15EG@Mt, and Gel/Gl/10CT@Mt active films.

pH Mean Values			
	Control	Gel/Gl/10CT@Mt	Gel/Gl/15EG@Mt
Day 0	5.65 ± 0.031 ^a,c^	5.65 ± 0.031 ^a,c^	5.65 ± 0.031 ^a,f,g^
Day 2	5.66 ± 0.021 ^a^	5.57 ± 0.006 ^c,d,f^	5.61 ± 0.040 ^a,c^
Day 4	5.67 ± 0.026 ^a^	5.52 ± 0.012 ^d,h^	5.58 ± 0.006 ^c,d,g^
Day 6	5.43 ± 0.04 ^b,c,d^	5.55 ± 0.02 ^e^	5.58 ± 0.010 ^c,h,g^

Different letters in each column indicate statistically significant differences at the confidence level *p* < 0.05. See also Table S6 in the Supplementary Materials.

**Table 7:** Variation in Lab* colorimetry parameters of coated minced meat during 6 days of storage.

Sample	Day	L (Mean ± SD)	a (Mean ± SD)	b (Mean ± SD)	ΔL (Mean ± SD)	Δa (Mean ± SD)	Δb (Mean ± SD)	ΔE (Mean ± SD)
**Control**	**0**	49.50 ± 1.20	21.30 ± 1.00	13.40 ± 0.90	0 ^a^	0 ^a^	0 ^a^	0 ^a^
	**2**	47.20 ± 1.30	18.50 ± 1.10	14.70 ± 1.00	–2.30 ± 0.50 ^Cb^	–2.80 ± 0.60 ^Cb^	1.30 ± 0.50 ^Cb^	3.85 ± 0.60 ^Cb^
	**4**	44.80 ± 1.40	15.20 ± 1.20	15.60 ± 1.10	–4.70 ± 0.60 ^Cc^	–6.10 ± 0.70 ^Cc^	2.20 ± 0.60 ^Cc^	8.01 ± 0.70 ^Cc^
	**6**	42.30 ± 1.50	12.00 ± 1.30	16.80 ± 1.30	–7.20 ± 0.70 ^Cd^	–9.30 ± 0.80 ^Cd^	3.30 ± 1.42 ^Cd^	6.75 ± 3.19 ^Cd^
**Gel/Gl/10CT@Mt**	**0**	—	—	—	—	—	—	—
	**2**	51.20 ± 1.10	18.80 ± 1.00	13.00 ± 0.90	–0.80 ± 0.40 ^Bb^	–1.40 ± 0.50 ^Bb^	0.30 ± 0.40 ^Bb^	1.77 ± 0.50 ^Bb^
	**4**	50.30 ± 1.20	17.10 ± 1.10	13.40 ± 0.90	–1.70 ± 0.50 ^Bc^	–3.10 ± 0.60 ^Bc^	0.70 ± 0.50 ^Bc^	2.99 ± 0.60 ^Bc^
	**6**	48.90 ± 1.30	15.50 ± 1.10	13.80 ± 1.00	–3.10 ± 0.60 ^Bd^	–5.55 ± 1.27 ^Bd^	0.73 ± 0.94 ^Bd^	3.69 ± 1.72 ^Bd^
**Gel/Gl/15EG@Mt**	**0**	—	—	—	—	—	—	—
	**2**	53.70 ± 1.00	19.90 ± 0.90	12.40 ± 0.80	–0.60 ± 0.30 ^Ab^	–0.90 ± 0.30 ^Ab^	0.30 ± 0.30 ^Ab^	1.16 ± 0.40 ^Ab^
	**4**	52.90 ± 1.10	18.60 ± 0.90	12.70 ± 0.80	–1.40 ± 0.40 ^Ac^	–2.20 ± 0.40 ^Ac^	0.60 ± 0.30 ^Ac^	2.41 ± 0.50 ^Ac^
	**6**	52.00 ± 1.20	17.80 ± 0.90	12.90 ± 0.90	–2.30 ± 0.50 ^Ad^	–4.48 ± 1.12Ad	0.55 ± 0.98 ^Ad^	2.64 ± 0.78 ^Ad^

Superscript letters indicate statistically significant differences (Tukey’s HSD, *p* < 0.05). Capital letters (A, B, C) denote differences between treatments (control, citral, eugenol) on the same day. Lowercase letters (a, b, c, d) indicate differences within the same treatment over storage time (day 0, 2, 4, 6). Means in the same column or row that do not share a common letter differ significantly.

**Table 8:** Sensory analysis (color, odor, taste) of coated minced meat during 6 days of storage.

Day		Color	Odor	Taste
**control**	0	5.00 ± 0.000 ^a^	5.00 ± 0.000 ^a^	5.00 ± 0.000 ^a^
	2	4.58 ± 0.330 ^a,d^	4.18 ± 0.538 ^a,b^	4.70 ± 0.183 ^a,b^
	4	3.90 ± 0.271 ^b,g^	3.50 ± 0.735 ^b,c^	3.98 ± 0.340 ^b,c^
	6	2.98 ± 0.330 ^c^	3.00 ± 0.812 ^c^	3.15 ± 0.520 ^c^
**Gel/Gl/10CT**	0	5.00 ± 0.000 ^a^	5.00 ± 0.000 ^a^	5.00 ± 0.000 ^a^
	2	4.73 ± 0.250 ^a,f^	4.63 ± 0.206 ^a,d^	4.75 ± 0.238 ^a,b^
	4	4.08 ± 0.222 ^b,d^	4.08 ± 0.150 ^a,c^	4.05 ± 0.265 ^b,c^
	6	3.10 ± 0.337 ^c^	3.55 ± 0.404 ^b,c,d^	3.43 ± 0.675 ^c,d^
**Gel/Gl/15EG@Mt**	0	5.00 ± 0.000 ^a^	5.00 ± 0.000 ^a^	5.00 ± 0.000 ^a^
	2	4.70 ± 0.216 ^a,f^	4.88 ± 0.096 ^a^	4.68 ± 0.150 ^a,b^
	4	4.18 ± 0.236 ^b,d,f^	4.45 ± 0.443 ^a,b^	4.23 ± 0.386 ^a,b,d^
	6	3.50 ± 0.000 ^c,g^	4.08 ± 0.690 ^a,c^	3.88 ± 0.660 ^b,c^

Different letters in each column indicate statistically significant differences at the confidence level *p* < 0.05. See also Table S8 in the Supplementary Materials.

## Data Availability

The datasets generated for this study are available on request to the corresponding author.

## Supplementary Materials

The following supporting information can be downloaded at: https://www.mdpi.com/article/10.3390/polym17111518/s1, Figure S1: Linear plots used for the calculation of average values of EC50 and EC60; Table S1: Tensile properties statistical analysis results; Table S2: Experimental data used for the calculation of obtained average EC50 and EC60 values; Table S3: Statistical analysis results of biocompatibility results; Table S4: Statistical analysis results of antibacterial activity results; Table S5: Statistical analysis results of TVC results; Table S6: Statistical analysis results of pH; Table S7: Statistical analysis results of Lab; Table S8: Statistical analysis results of sensory analysis.
