# Supplementary material for: Eugenol@Montmorillonite vs. Citral@Montmorillonite Nanohybrids for Gelatin-Based Extruded, Edible, High Oxygen Barrier, Active Packaging Films
Source: Polymers (Basel). 2025 May 29;17(11):1518. doi: 10.3390/polym17111518 (PMC12157017; doi:10.3390/polym17111518)
Supplement: Supplementary file 1 [file polymers-17-01518-s001.zip › polymers-3663379-supplementary.pdf]

Supplementary material for

Eugenol@Montmorillonite vs Citral@Montmorillonite nanohybrids for gelatin based extruded, edible, high oxygen barrier, active packaging films

Achilleas Kechagias[1], Areti A. Leontiou[1], Yelyzaveta K. Oliinychenko[2], Alexandros Ch. Stratakos[2], Konstatninos Zaharioudakis[1], Charalampos Proestos[3], Emmanuel P. Giannelis[4], Nikolaos Chalmpes[4]*, Constantinos E. Salmas[4,5]*, and Aris E. Giannakas[1]*

1 Department of Food Science and Technology, University of Patras, 30100 Agrinio, Greece; ch05654@uoi.gr (A.K.), aleontiu@upatras.gr (A.L.); zacharioudakis.k@upatras.gr (K.Z.);

2 School of Applied Sciences, College for Health, Science and Society, University of the West of England, Coldharbour Ln, Bristol, BS16 1QY, United Kingdom. Yelyzaveta2.Oliinychenko@live.uwe.ac.uk (Y.O.) Alexandros.stratakos@uwe.ac.uk (A.C.S)

3 Laboratory of Food Chemistry, Department of Chemistry, National and Kapodistrian University of Athens Zografou, 15771 Athens, Greece; harpro@chem.uoa.gr (C.P.)

4 Department of Materials Science and Engineering, Cornell University, Ithaca, New York 14850, United States; epg2@cornell.edu (E.P.G.)

5 Department of Material Science and Engineering, University of Ioannina, 45110 Ioannina, Greece;

*Correspondence: ksalmas@uoi.gr (C.E.S.), nc427@cornell.edu (N.C.), agiannakas@upatras.gr (A.E.G.);

X-Ray Diffraction (XRD) studies

Obtained EG@Mt and CT@Mt nanohybrids pure Mt as well as all obtained Gel/Gl/xMt, Gel/Gl/xEG@Mt and Gel/Gl/xCT@Mt films and pure Gel/Gl film were characterized with X-Ray Diffraction (XRD) analysis using a Brüker XRD D8 Advance diffractometer (Brüker, Analytical Instruments, S.A., Athens, Greece).

Fourier Transform Infrared Spectroscopy (FTIR) studies

FTIR spectra of pure EG, CT oils, EG@Mt and CT@Mt nanohybrids pure Mt as well as all obtained Gel/Gl/xMt, Gel/Gl/xEG@Mt and Gel/Gl/xCT@Mt films and pure Gel/Gl film were recorded using an FT/IR-6000 JASCO Fourier-transform spectrometer (JASCO, Interlab, S.A., Athens, Greece). Measurements were carried out using the KBr (0.5 %wt. to 1 %wt.) tablet technique. The spectra recorded over the wavenumber range from 4000 to 400 cm^{-1} at a resolution of 4 cm^{-1} and 64 scans were averaged to reduce noise.

Scanning Electron Microscopy (SEM) studies

High Resolution Scanning electron microscopy (HR-SEM) images of pure Mt, obtained EG@Mt and CT@Mt nanohybrids as well as were all obtained Gel/Gl/xMt, Gel/Gl/xEG@Mt and Gel/Gl/xCT@Mt films acquired using a Zeiss Gemini 500 SEM at a low accelerating voltage of 3 kV to reduce the excitation volume and enhance resolution.

EG release kinetic studies of EG@Lap and EG@Mt nanohybrids

Briefly, approximately 100 mg of each nanohybrid was placed in the moisture analyzer and its weight was recorded (in triplicates) as a function of time (m_t) at 70, 90, and 110 °C. From the obtained m_t vs t measurements, the normalized values of the fraction $q_t=(1-m_t/m_0)$ were calculated and plotted as a function of time. The plots were fitted using the well-known pseudo-second-order adsorption-desorption equation [1,2]. For process order, n=2 the overall normalized mass balance is given by:

$$\frac{dq_t}{dt} = k_2 * (q_e - q_t)^2 \quad (1)$$

where k_2 is the rate constant of the pseudo-second-order kinetic model (s^{-1}), qt is the desorbed fraction capacity at time t, $q_e=(1-m_e/m_0)$ is the maximum desorbed fraction capacity at equilibrium, m_0 is the initial EOs loading into the nanohybrid, and mt is the EOs amount remaining in the nanohybrid at time t. By integrating equation (1) we achieve the pseudo-second-order kinetic model:

$$q_t = (1 - \frac{m_t}{m_0}) = \frac{q_e^2 * k_2 * t}{q_e * k_2 * t + 1} \quad (2)$$

The initial release rate can be computed via the equation (1) and for t=0 (i.e., $q_t=0$). Thus:

$$r_i = \left.\frac{dq_t}{dt}\right|_{t=0} = k_2 * q_e^2 \quad (3)$$

From the best-fitted plots, the k_2 and q_e values were calculated. Using the estimated k_2 parameter the $\ln(k_2)$ term was calculated and plotted as a function of (1/T) to determine the desorption energy (E^0_{des}) according to the Arrhenius equation and the theory presented in detail in [3–5]:

$$k_2 = k_0 \cdot e^{-\frac{E^0_{des}}{R \cdot T}} \quad (4)$$

and its linear transformed type:

$$ln(k_2) = ln(k_0) - \frac{E^0_{des}}{R \cdot T} \quad (5)$$

where k_2 is the rate constant of the pseudo-second order kinetic model (s^{-1}), E^0_{des} is the desorption activation energy, and A is the Arrhenius constant.

Tensile properties of Gel/Gl/xMt, Gel/Gl/xEG@Mt and Gel/Gl/xCT@Mt films

Tensile properties of Gel/Gl/xMt, Gel/Gl/xEG@Mt and Gel/Gl/xCT@Mt films as well as pure Gel/Gl film were determined according to the American Society for Testing and Materials (ASTM) D638 method, by employing a Shimadzu AG-Xplus (5 kN), instrument (Shimadzü, Kyoto, Japan) and the methodology described in detail recently [6].

In Vitro Antioxidant Activity Determination of Gel/Gl/xMt, Gel/Gl/xEG@Mt and Gel/Gl/xCT@Mt films

For the CT based active films the concentration required to obtain a 50% antioxidant effect (EC_{50}) was calculated according to the methodology described recently [6] while for the EG based active films the concentration required to obtain a 60% antioxidant effect (EC_{60}) was calculated according to the methodology described recently by Karabagias et al. [8].

For the preparation of [DPPH•] free radical standard solutions, 0.0212 g of [DPPH•] free radical was dissolved in 250 mL of methanol to obtain a 2.16 mM (mmol/L) methanolic solution. Next, the flask was vortexed under dark conditions, and its pH (Milwaukee MW102-FOOD PRO+ 2-in-1 pH and Temperature Meter) was measured to ensure its neutrality (7.02 ± 0.01). Finally, the solution was placed in a refrigerator at 4 ± 1 °C under dark conditions for stabilization.

For the preparation of a [DPPH•] free radical calibration curve, 2.16 mM (mmol/L) methanolic solution of [DPPH•] free radical was diluted by adding appropriate volumes of methanol to obtain

concentrations of 10, 20, 30, 40, and 50 mg/L, and their absorbance was measured with a SHIMADZU UV-1280 UV/VIS Spectrometer at 517 nm. The calibration curve of absorbance (y) versus the concentration (x) of [DPPH•] free radical was expressed by the following equation:

$$y = 0.0388x + 0.015; R^2 = 0.9994 \quad (6)$$

For the determination of the concentration required to obtain a 50% or 60% antioxidant effect (EC_{50}) from all obtained films, 5 10, 20, 30, and 40 mg of granule film were placed in dark vials and three replicates were performed for each sample. Thereafter, 3 mL of [DPPH•] free radical methanolic solution and 2 mL of acetate buffer 100 mM (pH = 7.10) were added to each vial, and the absorbance of the reaction mixture was measured at 517 nm after 24 h. For a blank sample, we used a vial containing 3 mL of [DPPH•] free radical methanolic solution and 2 mL of acetate buffer without the addition of any granule film. The % inhibition of [DPPH•] was calculated using the following equation:

$$\% \text{ scavenged DPPH}^{\bullet} \text{ at steady state } = \frac{A_0^{517} - A_{sample}^{517}}{A_0^{517}} \times 100 \quad (7)$$

In vitro biocompatibility assessment of Gel/Gl/xEG@Mt and Gel/Gl/xCT@Mt films

To evaluate biocompatibility, epidermal human keratinocytes (HaCaT cells) were seeded at 2.5 × 10^5 cells/mL directly onto sterilized film samples or onto standard well surfaces (control). Cells were incubated for 24 hours at 37 °C with 5% CO_2 in Dulbecco's Modified Eagle Medium (DMEM) supplemented with 4.5 g/L glucose, 10% heat-inactivated fetal bovine serum (FBS), 100 U/mL penicillin, 100 mg/mL streptomycin, and 2 mM L-glutamine (all reagents from Merck, UK).

Film samples were prepared by cutting them into circular pieces, followed by UV sterilization for 1 hour per side and 24-hour immersion in culture medium at 37 °C prior to cell seeding. Cells were cultured in direct contact with the films according to Connolly et al. (2019) *[9]*. Cell viability was assessed using the alamarBlue® assay, a metabolic activity-based viability according to Stratakos et al., (2016) *[10]*. Following 1 h incubation at 37°C/5% CO_2 with 1.25% (v/v) alamarBlue® (Merck, UK), Absorption was measured at 570 and 600 nm using an automatic plate reader to determine cell viability. Results were expressed as a percentage relative to control cells.

Antibacterial activity of films

Frozen stock cultures of *Escherichia coli* ATCC 25922 and *Listeria monocytogenes* WDCM 00021 were streaked onto Tryptone Soya Agar (TSA; Oxoid, UK) and incubated at 37 °C for 24 hours. Single colonies were transferred to Brain Heart Infusion Broth (BHI; Oxoid, UK) and incubated under the same conditions. Cultures were then centrifuged (6,500 × g, 10 min), resuspended in Davis Minimal Broth (DMB; Sigma, UK), and adjusted to OD_{600} = 1.0 before use.

The method was adapted from Turalija et al. (2016) and Ardjoum et al. (2021) *[11,12]*. Briefly, each film (0.25 g) was cut into small pieces and placed in individual sterile falcon tubes containing 5 mL of bacterial suspension (10^5 CFU/mL) in DMB. The tubes were incubated at 30°C with gentle agitation (120 rpm) for 18 hours. After incubation, bacterial suspensions were serially diluted in 0.8% saline and spread-plated on Nutrient Agar or TSA (Oxoid, UK). Plates were incubated at 37°C for 24 hours before enumeration, with microbial counts expressed as log CFU/mL using equation 1:

$$log\ CFU\ /\ mL\ = \frac{log10\ (number\ of\ colonies\ counted}{(dilution\ factor \times volume\ plated\ (mL))} \quad (8)$$

Total Viable Count (TVC) of pork minced

TVC was monitored with respect to storage time at 4 ± 1 °C (0, 2, 4, and 6 days). Ten grams of pork minced were removed aseptically from each packaging system and transferred to glass beaker, containing 90 mL of sterile buffered peptone water (BPW, NCM0015A, Heywood, UK; 0.1 g/100 mL of distilled water) and vigorous stirred to homogenized for 90 s at room temperature. For the microbial enumeration, 0.1 mL of serial dilutions (1:10 diluents, buffered peptone water) of pork meat homogenates was spread on the surface of plate count agar (PCA, NCM0010A, Heywood, UK). TVC was determined after incubation for 2 days at 30 °C.

pH Analysis of pork minced

The pH values of the minced pork meat were measured using a portable pH meter fitted with a penetration electrode and a temperature sensor (pH-Star, Matthäus GmbH, Poettmes, Germany) by following the procedure described in detail recently [13]. To ensure accuracy and reliability, the procedure was conducted in triplicate, and for each treatment group, ten separate pH readings were taken.

Lab* Analysis

The alterations in the CIELAB color parameters (L*, a*, and b*) of the minced pork meat over a period of 10 days of storage were assessed using a LS171 colorimeter from the Linshang Company. Color evaluations were conducted directly on the surface of the minced pork meat, with each treatment group comprising three separate portions. For each of these portions, five discrete readings were taken to capture a robust assessment of the color. The total color differences (ΔE) were calculated using the following equation:

$$\Delta E = \sqrt{(L^* - L_0^*)^2 + (a^* - a_0^*)^2 + (b^* - b_0^*)^2} \quad (11)$$

In this equation, L*0, a*0, b*0 denote the initial color parameters of the minced pork meat at Day 0 post-treatment. L*, a*, b* represent the respective color parameters at different time points during the 10 days of storage at 4 °C.

Sensory Analysis of Pork Fillets

The sensory properties of pork meat were scaled from 0 (for the least liked sample) to 5 (most liked sample) points by seven experienced members of the Food Science and Technology Department. On each sampling day, color, odor, and cohesion were evaluated.

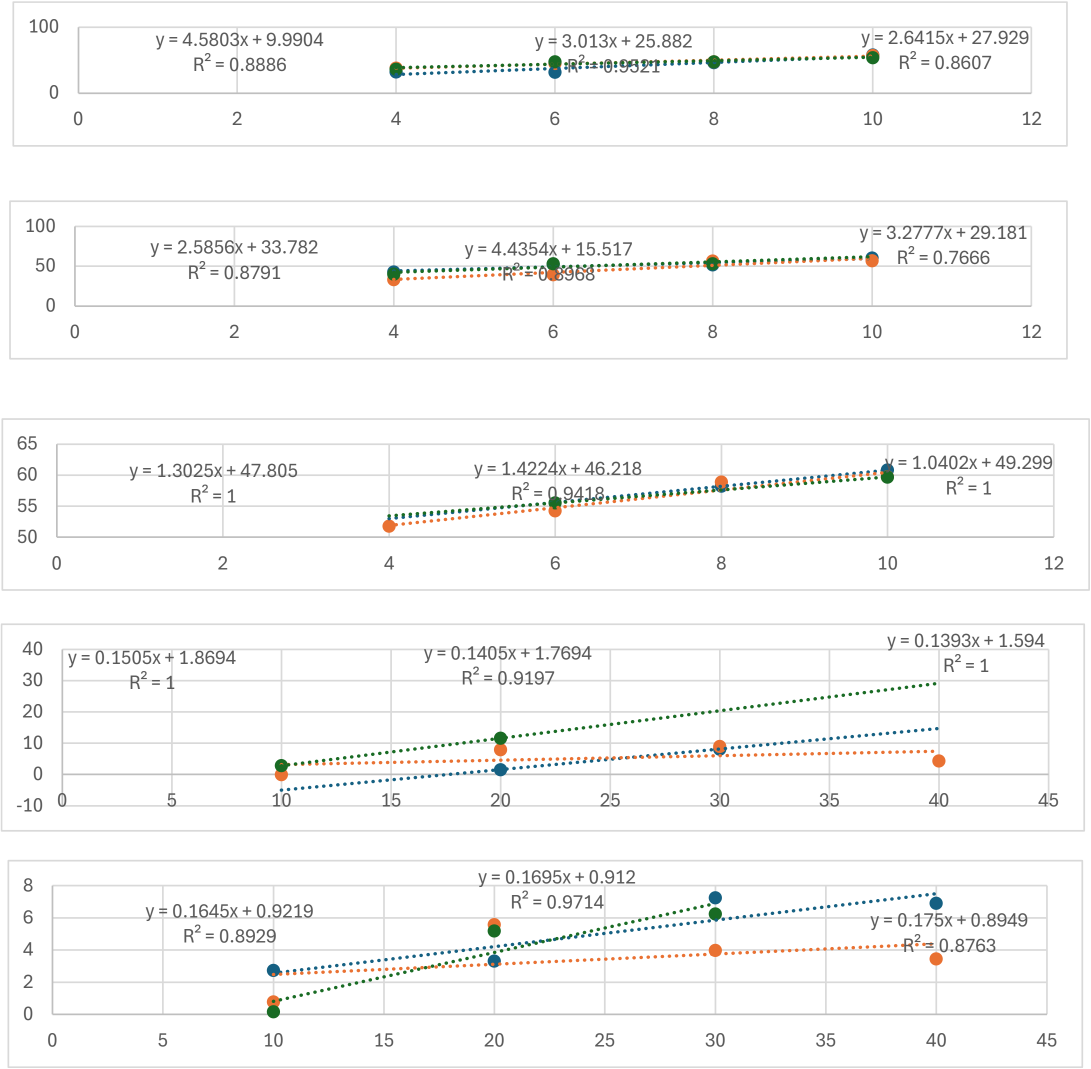

Figure S1. Linear plots used for the calculation of average values of EC_{50} and EC_{60}.

Table S1. Tensile properties statistical analysis results

Descriptives

		N	Mean	Std. Deviation	Std. Error	95% Confidence Interval for Mean		Minimum	Maximum
						Lower Bound	Upper Bound		
E	Gel_G	3	417,1533	38,56693	22,26663	321,3478	512,9589	376,71	453,52

	Gel_G_5MT	3	2161,4333	386,37125	223,07155	1201,6339	3121,2327	1764,00	2535,70
	Gel_G_10MT	3	1897,7667	320,39066	184,97763	1101,8722	2693,6612	1651,90	2260,10
	Gel_G_5EG@MT	3	968,5667	16,94589	9,78372	926,4707	1010,6626	950,00	983,20
	Gel_G_10EG@MT	4	1030,4000	113,34181	56,67091	850,0479	1210,7521	901,30	1167,20
	Gel_G_15EG@MT	4	1065,0000	48,28002	24,14001	988,1757	1141,8243	995,80	1103,20
	Gel_G_5CT@MT	3	690,1333	89,07336	51,42653	468,8628	911,4038	621,90	790,90
	Gel_G_10CT@MT	3	870,7667	126,43181	72,99544	556,6926	1184,8407	725,80	958,20
	Total	26	1130,7331	565,75613	110,95391	902,2192	1359,2469	376,71	2535,70
σuts	Gel_G	3	14,7267	1,61593	,93296	10,7125	18,7409	12,89	15,93
	Gel_G_5MT	3	36,1667	3,40343	1,96497	27,7121	44,6213	33,50	40,00
	Gel_G_10MT	3	31,5000	3,72424	2,15019	22,2485	40,7515	27,20	33,70
	Gel_G_5EG@MT	3	18,3567	2,14621	1,23912	13,0252	23,6882	16,02	20,24
	Gel_G_10EG@MT	4	32,2750	2,93641	1,46820	27,6025	36,9475	29,60	35,90
	Gel_G_15EG@MT	4	25,9000	2,02978	1,01489	22,6702	29,1298	24,00	28,20
	Gel_G_5CT@MT	3	20,6867	,96397	,55655	18,2920	23,0813	19,59	21,40
	Gel_G_10CT@MT	3	20,4467	2,50899	1,44857	14,2140	26,6793	17,65	22,50
	Total	26	25,3212	7,47837	1,46663	22,3006	28,3417	12,89	40,00
elongation	Gel_G	3	70,9433	13,14943	7,59183	38,2783	103,6083	60,32	85,65
	Gel_G_5MT	3	2,4767	,97208	,56123	,0619	4,8914	1,71	3,57
	Gel_G_10MT	3	2,2433	,74144	,42807	,4015	4,0852	1,71	3,09
	Gel_G_5EG@MT	3	2,3767	,22502	,12991	1,8177	2,9356	2,15	2,60
	Gel_G_10EG@MT	4	28,7250	12,14039	6,07020	9,4069	48,0431	19,90	45,90
	Gel_G_15EG@MT	4	31,7000	10,55115	5,27557	14,9108	48,4892	25,00	47,30
	Gel_G_5CT@MT	3	3,5967	,50846	,29356	2,3336	4,8598	3,15	4,15
	Gel_G_10CT@MT	3	2,7467	,25403	,14667	2,1156	3,3777	2,60	3,04
	Total	26	19,0327	23,86478	4,68027	9,3935	28,6719	1,71	85,65

Multiple Comparisons

Tukey HSD

Dependent Variable	(I) Film_Code	(J) Film_Code	Std. Error	Sig.	95% Confidence Interval

			Mean Difference (I-J)			Lower Bound	Upper Bound
E	Gel_G	Gel_G_5MT	-1744,28000*	149,16870	<,001	-2253,1408	-1235,4192
		Gel_G_10MT	-1480,61333*	149,16870	<,001	-1989,4741	-971,7526
		Gel_G_5EG@MT	-551,41333*	149,16870	,028	-1060,2741	-42,5526
		Gel_G_10EG@MT	-613,24667*	139,53454	,007	-1089,2423	-137,2510
		Gel_G_15EG@MT	-647,84667*	139,53454	,004	-1123,8423	-171,8510
		Gel_G_5CT@MT	-272,98000	149,16870	,610	-781,8408	235,8808
		Gel_G_10CT@MT	-453,61333	149,16870	,101	-962,4741	55,2474
	Gel_G_5MT	Gel_G	1744,28000*	149,16870	<,001	1235,4192	2253,1408
		Gel_G_10MT	263,66667	149,16870	,647	-245,1941	772,5274
		Gel_G_5EG@MT	1192,86667*	149,16870	<,001	684,0059	1701,7274
		Gel_G_10EG@MT	1131,03333*	139,53454	<,001	655,0377	1607,0290
		Gel_G_15EG@MT	1096,43333*	139,53454	<,001	620,4377	1572,4290
		Gel_G_5CT@MT	1471,30000*	149,16870	<,001	962,4392	1980,1608
		Gel_G_10CT@MT	1290,66667*	149,16870	<,001	781,8059	1799,5274
	Gel_G_10MT	Gel_G	1480,61333*	149,16870	<,001	971,7526	1989,4741
		Gel_G_5MT	-263,66667	149,16870	,647	-772,5274	245,1941
		Gel_G_5EG@MT	929,20000*	149,16870	<,001	420,3392	1438,0608
		Gel_G_10EG@MT	867,36667*	139,53454	<,001	391,3710	1343,3623
		Gel_G_15EG@MT	832,76667*	139,53454	<,001	356,7710	1308,7623
		Gel_G_5CT@MT	1207,63333*	149,16870	<,001	698,7726	1716,4941
		Gel_G_10CT@MT	1027,00000*	149,16870	<,001	518,1392	1535,8608
	Gel_G_5EG@MT	Gel_G	551,41333*	149,16870	,028	42,5526	1060,2741
		Gel_G_5MT	-1192,86667*	149,16870	<,001	-1701,7274	-684,0059
		Gel_G_10MT	-929,20000*	149,16870	<,001	-1438,0608	-420,3392
		Gel_G_10EG@MT	-61,83333	139,53454	1,000	-537,8290	414,1623
		Gel_G_15EG@MT	-96,43333	139,53454	,996	-572,4290	379,5623
		Gel_G_5CT@MT	278,43333	149,16870	,588	-230,4274	787,2941
		Gel_G_10CT@MT	97,80000	149,16870	,997	-411,0608	606,6608
	Gel_G_10EG@MT	Gel_G	613,24667*	139,53454	,007	137,2510	1089,2423
		Gel_G_5MT	-1131,03333*	139,53454	<,001	-1607,0290	-655,0377
		Gel_G_10MT	-867,36667*	139,53454	<,001	-1343,3623	-391,3710

		Gel_G_5EG@MT	61,83333	139,53454	1,000	-414,1623	537,8290
		Gel_G_15EG@MT	-34,60000	129,18388	1,000	-475,2864	406,0864
		Gel_G_5CT@MT	340,26667	139,53454	,281	-135,7290	816,2623
		Gel_G_10CT@MT	159,63333	139,53454	,938	-316,3623	635,6290
	Gel_G_15EG@MT	Gel_G	647,84667*	139,53454	,004	171,8510	1123,8423
		Gel_G_5MT	-1096,43333*	139,53454	<,001	-1572,4290	-620,4377
		Gel_G_10MT	-832,76667*	139,53454	<,001	-1308,7623	-356,7710
		Gel_G_5EG@MT	96,43333	139,53454	,996	-379,5623	572,4290
		Gel_G_10EG@MT	34,60000	129,18388	1,000	-406,0864	475,2864
		Gel_G_5CT@MT	374,86667	139,53454	,189	-101,1290	850,8623
		Gel_G_10CT@MT	194,23333	139,53454	,849	-281,7623	670,2290
	Gel_G_5CT@MT	Gel_G	272,98000	149,16870	,610	-235,8808	781,8408
		Gel_G_5MT	-1471,30000*	149,16870	<,001	-1980,1608	-962,4392
		Gel_G_10MT	-1207,63333*	149,16870	<,001	-1716,4941	-698,7726
		Gel_G_5EG@MT	-278,43333	149,16870	,588	-787,2941	230,4274
		Gel_G_10EG@MT	-340,26667	139,53454	,281	-816,2623	135,7290
		Gel_G_15EG@MT	-374,86667	139,53454	,189	-850,8623	101,1290
		Gel_G_10CT@MT	-180,63333	149,16870	,918	-689,4941	328,2274
	Gel_G_10CT@MT	Gel_G	453,61333	149,16870	,101	-55,2474	962,4741
		Gel_G_5MT	-1290,66667*	149,16870	<,001	-1799,5274	-781,8059
		Gel_G_10MT	-1027,00000*	149,16870	<,001	-1535,8608	-518,1392
		Gel_G_5EG@MT	-97,80000	149,16870	,997	-606,6608	411,0608
		Gel_G_10EG@MT	-159,63333	139,53454	,938	-635,6290	316,3623
		Gel_G_15EG@MT	-194,23333	139,53454	,849	-670,2290	281,7623
		Gel_G_5CT@MT	180,63333	149,16870	,918	-328,2274	689,4941
σuts	Gel_G	Gel_G_5MT	-21,44000*	2,09070	<,001	-28,5720	-14,3080
		Gel_G_10MT	-16,77333*	2,09070	<,001	-23,9054	-9,6413
		Gel_G_5EG@MT	-3,63000	2,09070	,666	-10,7620	3,5020
		Gel_G_10EG@MT	-17,54833*	1,95567	<,001	-24,2197	-10,8769
		Gel_G_15EG@MT	-11,17333*	1,95567	<,001	-17,8447	-4,5019
		Gel_G_5CT@MT	-5,96000	2,09070	,143	-13,0920	1,1720
		Gel_G_10CT@MT	-5,72000	2,09070	,174	-12,8520	1,4120

	Gel_G_5MT	Gel_G	21,44000*	2,09070	<,001	14,3080	28,5720
		Gel_G_10MT	4,66667	2,09070	,379	-2,4654	11,7987
		Gel_G_5EG@MT	17,81000*	2,09070	<,001	10,6780	24,9420
		Gel_G_10EG@MT	3,89167	1,95567	,514	-2,7797	10,5631
		Gel_G_15EG@MT	10,26667*	1,95567	,001	3,5953	16,9381
		Gel_G_5CT@MT	15,48000*	2,09070	<,001	8,3480	22,6120
		Gel_G_10CT@MT	15,72000*	2,09070	<,001	8,5880	22,8520
	Gel_G_10MT	Gel_G	16,77333*	2,09070	<,001	9,6413	23,9054
		Gel_G_5MT	-4,66667	2,09070	,379	-11,7987	2,4654
		Gel_G_5EG@MT	13,14333*	2,09070	<,001	6,0113	20,2754
		Gel_G_10EG@MT	-,77500	1,95567	1,000	-7,4464	5,8964
		Gel_G_15EG@MT	5,60000	1,95567	,140	-1,0714	12,2714
		Gel_G_5CT@MT	10,81333*	2,09070	,001	3,6813	17,9454
		Gel_G_10CT@MT	11,05333*	2,09070	,001	3,9213	18,1854
	Gel_G_5EG@MT	Gel_G	3,63000	2,09070	,666	-3,5020	10,7620
		Gel_G_5MT	-17,81000*	2,09070	<,001	-24,9420	-10,6780
		Gel_G_10MT	-13,14333*	2,09070	<,001	-20,2754	-6,0113
		Gel_G_10EG@MT	-13,91833*	1,95567	<,001	-20,5897	-7,2469
		Gel_G_15EG@MT	-7,54333*	1,95567	,020	-14,2147	-,8719
		Gel_G_5CT@MT	-2,33000	2,09070	,945	-9,4620	4,8020
		Gel_G_10CT@MT	-2,09000	2,09070	,969	-9,2220	5,0420
	Gel_G_10EG@MT	Gel_G	17,54833*	1,95567	<,001	10,8769	24,2197
		Gel_G_5MT	-3,89167	1,95567	,514	-10,5631	2,7797
		Gel_G_10MT	,77500	1,95567	1,000	-5,8964	7,4464
		Gel_G_5EG@MT	13,91833*	1,95567	<,001	7,2469	20,5897
		Gel_G_15EG@MT	6,37500*	1,81060	,040	,1985	12,5515
		Gel_G_5CT@MT	11,58833*	1,95567	<,001	4,9169	18,2597
		Gel_G_10CT@MT	11,82833*	1,95567	<,001	5,1569	18,4997
	Gel_G_15EG@MT	Gel_G	11,17333*	1,95567	<,001	4,5019	17,8447
		Gel_G_5MT	-10,26667*	1,95567	,001	-16,9381	-3,5953
		Gel_G_10MT	-5,60000	1,95567	,140	-12,2714	1,0714
		Gel_G_5EG@MT	7,54333*	1,95567	,020	,8719	14,2147

		Gel_G_10EG@MT	-6,37500*	1,81060	,040	-12,5515	-,1985
		Gel_G_5CT@MT	5,21333	1,95567	,196	-1,4581	11,8847
		Gel_G_10CT@MT	5,45333	1,95567	,159	-1,2181	12,1247
	Gel_G_5CT@MT	Gel_G	5,96000	2,09070	,143	-1,1720	13,0920
		Gel_G_5MT	-15,48000*	2,09070	<,001	-22,6120	-8,3480
		Gel_G_10MT	-10,81333*	2,09070	,001	-17,9454	-3,6813
		Gel_G_5EG@MT	2,33000	2,09070	,945	-4,8020	9,4620
		Gel_G_10EG@MT	-11,58833*	1,95567	<,001	-18,2597	-4,9169
		Gel_G_15EG@MT	-5,21333	1,95567	,196	-11,8847	1,4581
		Gel_G_10CT@MT	,24000	2,09070	1,000	-6,8920	7,3720
	Gel_G_10CT@MT	Gel_G	5,72000	2,09070	,174	-1,4120	12,8520
		Gel_G_5MT	-15,72000*	2,09070	<,001	-22,8520	-8,5880
		Gel_G_10MT	-11,05333*	2,09070	,001	-18,1854	-3,9213
		Gel_G_5EG@MT	2,09000	2,09070	,969	-5,0420	9,2220
		Gel_G_10EG@MT	-11,82833*	1,95567	<,001	-18,4997	-5,1569
		Gel_G_15EG@MT	-5,45333	1,95567	,159	-12,1247	1,2181
		Gel_G_5CT@MT	-,24000	2,09070	1,000	-7,3720	6,8920
elongation	Gel_G	Gel_G_5MT	68,46667*	6,45698	<,001	46,4399	90,4934
		Gel_G_10MT	68,70000*	6,45698	<,001	46,6732	90,7268
		Gel_G_5EG@MT	68,56667*	6,45698	<,001	46,5399	90,5934
		Gel_G_10EG@MT	42,21833*	6,03995	<,001	21,6142	62,8225
		Gel_G_15EG@MT	39,24333*	6,03995	<,001	18,6392	59,8475
		Gel_G_5CT@MT	67,34667*	6,45698	<,001	45,3199	89,3734
		Gel_G_10CT@MT	68,19667*	6,45698	<,001	46,1699	90,2234
	Gel_G_5MT	Gel_G	-68,46667*	6,45698	<,001	-90,4934	-46,4399
		Gel_G_10MT	,23333	6,45698	1,000	-21,7934	22,2601
		Gel_G_5EG@MT	,10000	6,45698	1,000	-21,9268	22,1268
		Gel_G_10EG@MT	-26,24833*	6,03995	,007	-46,8525	-5,6442
		Gel_G_15EG@MT	-29,22333*	6,03995	,003	-49,8275	-8,6192
		Gel_G_5CT@MT	-1,12000	6,45698	1,000	-23,1468	20,9068
		Gel_G_10CT@MT	-,27000	6,45698	1,000	-22,2968	21,7568
	Gel_G_10MT	Gel_G	-68,70000*	6,45698	<,001	-90,7268	-46,6732

		Gel_G_5MT	-,23333	6,45698	1,000	-22,2601	21,7934
		Gel_G_5EG@MT	-,13333	6,45698	1,000	-22,1601	21,8934
		Gel_G_10EG@MT	-26,48167*	6,03995	,007	-47,0858	-5,8775
		Gel_G_15EG@MT	-29,45667*	6,03995	,002	-50,0608	-8,8525
		Gel_G_5CT@MT	-1,35333	6,45698	1,000	-23,3801	20,6734
		Gel_G_10CT@MT	-,50333	6,45698	1,000	-22,5301	21,5234
	Gel_G_5EG@MT	Gel_G	-68,56667*	6,45698	<,001	-90,5934	-46,5399
		Gel_G_5MT	-,10000	6,45698	1,000	-22,1268	21,9268
		Gel_G_10MT	,13333	6,45698	1,000	-21,8934	22,1601
		Gel_G_10EG@MT	-26,34833*	6,03995	,007	-46,9525	-5,7442
		Gel_G_15EG@MT	-29,32333*	6,03995	,003	-49,9275	-8,7192
		Gel_G_5CT@MT	-1,22000	6,45698	1,000	-23,2468	20,8068
		Gel_G_10CT@MT	-,37000	6,45698	1,000	-22,3968	21,6568
	Gel_G_10EG@MT	Gel_G	-42,21833*	6,03995	<,001	-62,8225	-21,6142
		Gel_G_5MT	26,24833*	6,03995	,007	5,6442	46,8525
		Gel_G_10MT	26,48167*	6,03995	,007	5,8775	47,0858
		Gel_G_5EG@MT	26,34833*	6,03995	,007	5,7442	46,9525
		Gel_G_15EG@MT	-2,97500	5,59191	,999	-22,0507	16,1007
		Gel_G_5CT@MT	25,12833*	6,03995	,011	4,5242	45,7325
		Gel_G_10CT@MT	25,97833*	6,03995	,008	5,3742	46,5825
	Gel_G_15EG@MT	Gel_G	-39,24333*	6,03995	<,001	-59,8475	-18,6392
		Gel_G_5MT	29,22333*	6,03995	,003	8,6192	49,8275
		Gel_G_10MT	29,45667*	6,03995	,002	8,8525	50,0608
		Gel_G_5EG@MT	29,32333*	6,03995	,003	8,7192	49,9275
		Gel_G_10EG@MT	2,97500	5,59191	,999	-16,1007	22,0507
		Gel_G_5CT@MT	28,10333*	6,03995	,004	7,4992	48,7075
		Gel_G_10CT@MT	28,95333*	6,03995	,003	8,3492	49,5575
	Gel_G_5CT@MT	Gel_G	-67,34667*	6,45698	<,001	-89,3734	-45,3199
		Gel_G_5MT	1,12000	6,45698	1,000	-20,9068	23,1468
		Gel_G_10MT	1,35333	6,45698	1,000	-20,6734	23,3801
		Gel_G_5EG@MT	1,22000	6,45698	1,000	-20,8068	23,2468
		Gel_G_10EG@MT	-25,12833*	6,03995	,011	-45,7325	-4,5242

		Gel_G_15EG@MT	-28,10333*	6,03995	,004	-48,7075	-7,4992
		Gel_G_10CT@MT	,85000	6,45698	1,000	-21,1768	22,8768
	Gel_G_10CT@MT	Gel_G	-68,19667*	6,45698	<,001	-90,2234	-46,1699
		Gel_G_5MT	,27000	6,45698	1,000	-21,7568	22,2968
		Gel_G_10MT	,50333	6,45698	1,000	-21,5234	22,5301
		Gel_G_5EG@MT	,37000	6,45698	1,000	-21,6568	22,3968
		Gel_G_10EG@MT	-25,97833*	6,03995	,008	-46,5825	-5,3742
		Gel_G_15EG@MT	-28,95333*	6,03995	,003	-49,5575	-8,3492
		Gel_G_5CT@MT	-,85000	6,45698	1,000	-22,8768	21,1768

*. The mean difference is significant at the 0.05 level.

Table S2. Experimental data used for the calculation of obtained average EC50 and EC60 values

			Απορρόφηση							
	mg Δείγματος	1		2		3				
DPPH		3,39 (3,02)								
A0		2.073		2.081		2.067				
		1	% AA	2	% AA	3	% AA		EC50	EC60
G-Gl_EG&MT5	4	1.405	32.22383	1.292	37.91446	1.317	36.28447	1	8.735148353	10.9184115
	6	1.415	31.74144	1.14	45.21864	1.08	47.75036	2	8.004646532	11.3235977
	8	1.112	46.35794	1.092	47.52523	1.08	47.75036	3	8.355479841	12.1412076
	10	0.873	57.88712	0.89	57.2321	0.953	53.89453		8.365091575	11.4610723
									0.36534575	0.62288207
		1	% AA	2	% AA	3	% AA		EC50	EC60
G-Gl_EG&MT10	4	1.184	42.88471	1.39	33.20519	1.236	40.20319	1	6.272431931	10.1400062
	6	0.986	52.43608	1.26	39.45219	0.971	53.02371	2	7.7744961	10.0290842
	8	0.997	51.90545	0.905	56.51129	0.965	53.31398	3	6.351710041	9.40262989
	10	0.823	60.29908	0.893	57.08794	1.077			6.799546024	9.85724009
									0.845261494	0.39759118
		1	% AA	2	% AA	3	% AA		EC50	EC60
G-Gl_EG&MT15	4	0.93		1.003	51.80202	0.856		1	1.685220729	9.36276392
	6	0.881		0.952	54.25276	0.919	55.53943	2	2.658886389	9.68925759
	8	0.866	58.22479	0.855	58.91398	0.817		3	0.673908864	10.2874447
	10	0.812	60.82972	0.838	59.7309	0.833	59.70005		1.672671994	9.77982208
									0.992548259	0.46894572
		1	% AA	2	% AA	3	% AA		EC50	EC60
A0		1.872		1.865		1.878		1	319.8046512	386.249834

G-Gl_CT&MT5	10	1.786		1.866	-0.05362	1.825	2.822151	2	343.2782918	414.452669
	20	1.843	1.549145	1.717	7.935657	1.66	11.60809	3	346.9964158	418.681004
	30	1.72	8.119658	1.698	8.954424	1.83			336.6931196	406.461169
	40	1.736		1.785	4.289544	1.789			14.74352012	17.6307473
		1	% AA	2	% AA	3	% AA		EC50	EC60
A0		2.061		2.067		2.069		1	298.3471125	359.137386
G-Gl_CT&MT10	10	2.005	2.717128	2.051	0.774069	2.066	0.144998	2	289.6047198	348.60177
	20	1.993	3.299369	1.952	5.563619	1.962	5.17158	3	280.6005714	280.600571
	30	1.912	7.2295	1.985	3.967102	1.94	6.234896		289.5174679	329.446576
	40	1.919	6.889859	1.996	3.43493				8.873592245	42.6286161

Table S3. Statistical analysis results of biocompatibility results

Tukey's multiple comparisons test	Mean Diff.	95.00% CI of diff.	Below threshold?	Summary	Adjusted P Value
Gel/Gl vs. Gel/Gl/5Mt	5.983	-1.468 to 13.43	No	ns	0.194982
Gel/Gl vs. Gel/Gl/10Mt	8.417	0.9655 to 15.87	Yes	*	0.017741
Gel/Gl vs. Gel/Gl/5EG@Mt	13.67	6.215 to 21.12	Yes	****	0.000026
Gel/Gl vs. Gel/Gl/10EG@Mt	16.32	8.865 to 23.77	Yes	****	<0.000001
Gel/Gl vs. Gel/Gl/15EG@Mt	19.65	12.20 to 27.10	Yes	****	<0.000001
Gel/Gl vs. Gel/Gl/5CT@Mt	10.52	3.065 to 17.97	Yes	**	0.001464
Gel/Gl vs. Gel/Gl/10CT@Mt	13.35	5.899 to 20.80	Yes	****	0.000039
Gel/Gl/5Mt vs. Gel/Gl/10Mt	2.433	-5.018 to 9.885	No	ns	0.962458
Gel/Gl/5Mt vs. Gel/Gl/5EG@Mt	7.683	0.2321 to 15.13	Yes	*	0.039309
Gel/Gl/5Mt vs. Gel/Gl/10EG@Mt	10.33	2.882 to 17.78	Yes	**	0.001837
Gel/Gl/5Mt vs. Gel/Gl/15EG@Mt	13.67	6.215 to 21.12	Yes	****	0.000026
Gel/Gl/5Mt vs. Gel/Gl/5CT@Mt	4.533	-2.918 to 11.98	No	ns	0.521836
Gel/Gl/5Mt vs. Gel/Gl/10CT@Mt	7.367	-0.08454 to 14.82	No	ns	0.054496
Gel/Gl/10Mt vs. Gel/Gl/5EG@Mt	5.250	-2.201 to 12.70	No	ns	0.338120
Gel/Gl/10Mt vs. Gel/Gl/10EG@Mt	7.900	0.4488 to 15.35	Yes	*	0.031242
Gel/Gl/10Mt vs. Gel/Gl/15EG@Mt	11.23	3.782 to 18.68	Yes	***	0.000596
Gel/Gl/10Mt vs. Gel/Gl/5CT@Mt	2.100	-5.351 to 9.551	No	ns	0.983320
Gel/Gl/10Mt vs. Gel/Gl/10CT@Mt	4.933	-2.518 to 12.38	No	ns	0.415170

Gel/Gl/5EG@Mt vs. Gel/Gl/10EG@Mt	2.650	-4.801 to 10.10	No	ns	0.941642
Gel/Gl/5EG@Mt vs. Gel/Gl/15EG@Mt	5.983	-1.468 to 13.43	No	ns	0.194982
Gel/Gl/5EG@Mt vs. Gel/Gl/5CT@Mt	-3.150	-10.60 to 4.301	No	ns	0.868002
Gel/Gl/5EG@Mt vs. Gel/Gl/10CT@Mt	-0.3167	-7.768 to 7.135	No	ns	>0.999999
Gel/Gl/10EG@Mt vs. Gel/Gl/15EG@Mt	3.333	-4.118 to 10.78	No	ns	0.831973
Gel/Gl/10EG@Mt vs. Gel/Gl/5CT@Mt	-5.800	-13.25 to 1.651	No	ns	0.225825
Gel/Gl/10EG@Mt vs. Gel/Gl/10CT@Mt	-2.967	-10.42 to 4.485	No	ns	0.899268
Gel/Gl/15EG@Mt vs. Gel/Gl/5CT@Mt	-9.133	-16.58 to -1.682	Yes	**	0.007804
Gel/Gl/15EG@Mt vs. Gel/Gl/10CT@Mt	-6.300	-13.75 to 1.151	No	ns	0.149296
Gel/Gl/5CT@Mt vs. Gel/Gl/10CT@Mt	2.833	-4.618 to 10.28	No	ns	0.918902

Table S4. Statistical analysis results of antibacterial activity results

Tukey's multiple comparisons test for ***L. monocytogenes***	Mean Diff.	95.00% CI of diff.	Below threshold?	Summary	Adjusted P Value
control vs. Gel/Gl	0.03333	-0.7844 to 0.8511	No	ns	>0.999999
control vs. Gel/Gl/5Mt	-0.2333	-1.051 to 0.5844	No	ns	0.978566
control vs. Gel/Gl/10Mt	-0.06667	-0.8844 to 0.7511	No	ns	0.999997
control vs. Gel/Gl/5EG@Mt	0.2667	-0.5511 to 1.084	No	ns	0.954224
control vs. Gel/Gl/10EG@Mt	3.433	2.616 to 4.251	Yes	****	<0.000001
control vs. Gel/Gl/15EG@Mt	5.833	5.016 to 6.651	Yes	****	<0.000001
control vs. Gel/Gl/5CT@Mt	0.1333	-0.6844 to 0.9511	No	ns	0.999487
control vs. Gel/Gl/10CT@Mt	1.467	0.6489 to 2.284	Yes	***	0.000240
Gel/Gl vs. Gel/Gl/5Mt	-0.2667	-1.084 to 0.5511	No	ns	0.954224
Gel/Gl vs. Gel/Gl/10Mt	-0.1000	-0.9177 to 0.7177	No	ns	0.999939
Gel/Gl vs. Gel/Gl/5EG@Mt	0.2333	-0.5844 to 1.051	No	ns	0.978566
Gel/Gl vs. Gel/Gl/10EG@Mt	3.400	2.582 to 4.218	Yes	****	<0.000001

Gel/Gl vs. Gel/Gl/15EG@Mt	5.800	4.982 to 6.618	Yes	****	<0.000001
Gel/Gl vs. Gel/Gl/5CT@Mt	0.1000	-0.7177 to 0.9177	No	ns	0.999939
Gel/Gl vs. Gel/Gl/10CT@Mt	1.433	0.6156 to 2.251	Yes	***	0.000312
Gel/Gl/5Mt vs. Gel/Gl/10Mt	0.1667	-0.6511 to 0.9844	No	ns	0.997528
Gel/Gl/5Mt vs. Gel/Gl/5EG@Mt	0.5000	-0.3177 to 1.318	No	ns	0.462614
Gel/Gl/5Mt vs. Gel/Gl/10EG@Mt	3.667	2.849 to 4.484	Yes	****	<0.000001
Gel/Gl/5Mt vs. Gel/Gl/15EG@Mt	6.067	5.249 to 6.884	Yes	****	<0.000001
Gel/Gl/5Mt vs. Gel/Gl/5CT@Mt	0.3667	-0.4511 to 1.184	No	ns	0.794506
Gel/Gl/5Mt vs. Gel/Gl/10CT@Mt	1.700	0.8823 to 2.518	Yes	****	0.000041
Gel/Gl/10Mt vs. Gel/Gl/5EG@Mt	0.3333	-0.4844 to 1.151	No	ns	0.862073
Gel/Gl/10Mt vs. Gel/Gl/10EG@Mt	3.500	2.682 to 4.318	Yes	****	<0.000001
Gel/Gl/10Mt vs. Gel/Gl/15EG@Mt	5.900	5.082 to 6.718	Yes	****	<0.000001
Gel/Gl/10Mt vs. Gel/Gl/5CT@Mt	0.2000	-0.6177 to 1.018	No	ns	0.991720
Gel/Gl/10Mt vs. Gel/Gl/10CT@Mt	1.533	0.7156 to 2.351	Yes	***	0.000143
Gel/Gl/5EG@Mt vs. Gel/Gl/10EG@Mt	3.167	2.349 to 3.984	Yes	****	<0.000001
Gel/Gl/5EG@Mt vs. Gel/Gl/15EG@Mt	5.567	4.749 to 6.384	Yes	****	<0.000001
Gel/Gl/5EG@Mt vs. Gel/Gl/5CT@Mt	-0.1333	-0.9511 to 0.6844	No	ns	0.999487
Gel/Gl/5EG@Mt vs. Gel/Gl/10CT@Mt	1.200	0.3823 to 2.018	Yes	**	0.002058
Gel/Gl/10EG@Mt vs. Gel/Gl/15EG@Mt	2.400	1.582 to 3.218	Yes	****	<0.000001
Gel/Gl/10EG@Mt vs. Gel/Gl/5CT@Mt	-3.300	-4.118 to -2.482	Yes	****	<0.000001
Gel/Gl/10EG@Mt vs. Gel/Gl/10CT@Mt	-1.967	-2.784 to -1.149	Yes	****	0.000006
Gel/Gl/15EG@Mt vs. Gel/Gl/5CT@Mt	-5.700	-6.518 to -4.882	Yes	****	<0.000001
Gel/Gl/15EG@Mt vs. Gel/Gl/10CT@Mt	-4.367	-5.184 to -3.549	Yes	****	<0.000001
Gel/Gl/5CT@Mt vs. Gel/Gl/10CT@Mt	1.333	0.5156 to 2.151	Yes	***	0.000691

Tukey's multiple comparisons test for ***E. coli***	Mean Diff.	95.00% CI of diff.	Below threshold?	Summary	Adjusted P Value
control vs. Gel/Gl	0.03333	-0.4854 to 0.5521	No	ns	>0.999999
control vs. Gel/Gl/5Mt	0.03333	-0.4854 to 0.5521	No	ns	>0.999999
control vs. Gel/Gl/10Mt	0.000	-0.5188 to 0.5188	No	ns	>0.999999
control vs. Gel/Gl/5EG@Mt	0.2000	-0.3188 to 0.7188	No	ns	0.893027
control vs. Gel/Gl/10EG@Mt	0.8333	0.3146 to 1.352	Yes	***	0.000811
control vs. Gel/Gl/15EG@Mt	6.400	5.881 to 6.919	Yes	****	<0.000001
control vs. Gel/Gl/5CT@Mt	0.2333	-0.2854 to 0.7521	No	ns	0.791985
control vs. Gel/Gl/10CT@Mt	0.8333	0.3146 to 1.352	Yes	***	0.000811
Gel/Gl vs. Gel/Gl/5Mt	0.000	-0.5188 to 0.5188	No	ns	>0.999999

Gel/Gl vs. Gel/Gl/10Mt	-0.03333	-0.5521 to 0.4854	No	ns	>0.999999
Gel/Gl vs. Gel/Gl/5EG@Mt	0.1667	-0.3521 to 0.6854	No	ns	0.957803
Gel/Gl vs. Gel/Gl/10EG@Mt	0.8000	0.2812 to 1.319	Yes	**	0.001244
Gel/Gl vs. Gel/Gl/15EG@Mt	6.367	5.848 to 6.885	Yes	****	<0.000001
Gel/Gl vs. Gel/Gl/5CT@Mt	0.2000	-0.3188 to 0.7188	No	ns	0.893027
Gel/Gl vs. Gel/Gl/10CT@Mt	0.8000	0.2812 to 1.319	Yes	**	0.001244
Gel/Gl/5Mt vs. Gel/Gl/10Mt	-0.03333	-0.5521 to 0.4854	No	ns	>0.999999
Gel/Gl/5Mt vs. Gel/Gl/5EG@Mt	0.1667	-0.3521 to 0.6854	No	ns	0.957803
Gel/Gl/5Mt vs. Gel/Gl/10EG@Mt	0.8000	0.2812 to 1.319	Yes	**	0.001244
Gel/Gl/5Mt vs. Gel/Gl/15EG@Mt	6.367	5.848 to 6.885	Yes	****	<0.000001
Gel/Gl/5Mt vs. Gel/Gl/5CT@Mt	0.2000	-0.3188 to 0.7188	No	ns	0.893027
Gel/Gl/5Mt vs. Gel/Gl/10CT@Mt	0.8000	0.2812 to 1.319	Yes	**	0.001244
Gel/Gl/10Mt vs. Gel/Gl/5EG@Mt	0.2000	-0.3188 to 0.7188	No	ns	0.893027
Gel/Gl/10Mt vs. Gel/Gl/10EG@Mt	0.8333	0.3146 to 1.352	Yes	***	0.000811
Gel/Gl/10Mt vs. Gel/Gl/15EG@Mt	6.400	5.881 to 6.919	Yes	****	<0.000001
Gel/Gl/10Mt vs. Gel/Gl/5CT@Mt	0.2333	-0.2854 to 0.7521	No	ns	0.791985
Gel/Gl/10Mt vs. Gel/Gl/10CT@Mt	0.8333	0.3146 to 1.352	Yes	***	0.000811
Gel/Gl/5EG@Mt vs. Gel/Gl/10EG@Mt	0.6333	0.1146 to 1.152	Yes	*	0.011158
Gel/Gl/5EG@Mt vs. Gel/Gl/15EG@Mt	6.200	5.681 to 6.719	Yes	****	<0.000001
Gel/Gl/5EG@Mt vs. Gel/Gl/5CT@Mt	0.03333	-0.4854 to 0.5521	No	ns	>0.999999
Gel/Gl/5EG@Mt vs. Gel/Gl/10CT@Mt	0.6333	0.1146 to 1.152	Yes	*	0.011158
Gel/Gl/10EG@Mt vs. Gel/Gl/15EG@Mt	5.567	5.048 to 6.085	Yes	****	<0.000001
Gel/Gl/10EG@Mt vs. Gel/Gl/5CT@Mt	-0.6000	-1.119 to -0.08123	Yes	*	0.017351
Gel/Gl/10EG@Mt vs. Gel/Gl/10CT@Mt	0.000	-0.5188 to 0.5188	No	ns	>0.999999
Gel/Gl/15EG@Mt vs. Gel/Gl/5CT@Mt	-6.167	-6.685 to -5.648	Yes	****	<0.000001
Gel/Gl/15EG@Mt vs. Gel/Gl/10CT@Mt	-5.567	-6.085 to -5.048	Yes	****	<0.000001
Gel/Gl/5CT@Mt vs. Gel/Gl/10CT@Mt	0.6000	0.08123 to 1.119	Yes	*	0.017351

Table S5. Statistical analysis results of TVC results

Descriptives

TVC

	N	Mean	Std. Deviation	Std. Error	95% Confidence Interval for Mean		Minimum	Maximum
					Lower Bound	Upper Bound		
CONTROL_Day0	2	4,2400	,19799	,14000	2,4611	6,0189	4,10	4,38
CONTROL_Day2	2	5,6400	,11314	,08000	4,6235	6,6565	5,56	5,72
CONTROL_Day4	2	6,8900	,07071	,05000	6,2547	7,5253	6,84	6,94
CONTROL_Day6	2	8,1350	,13435	,09500	6,9279	9,3421	8,04	8,23
G_GI_CT@MT10_Day0	2	4,2400	,19799	,14000	2,4611	6,0189	4,10	4,38
G_GI_CT@MT10_Day2	2	5,7450	,12021	,08500	4,6650	6,8250	5,66	5,83
G_GI_CT@MT10_Day4	2	5,8800	,04243	,03000	5,4988	6,2612	5,85	5,91
G_GI_CT@MT10_Day6	2	7,7600	,07071	,05000	7,1247	8,3953	7,71	7,81
G_GI_EG@MT15_Day0	2	4,2400	,19799	,14000	2,4611	6,0189	4,10	4,38
G_GI_EG@MT15_Day2	2	4,8050	,03536	,02500	4,4873	5,1227	4,78	4,83
G_GI_EG@MT15_Day4	2	6,1250	,07778	,05500	5,4262	6,8238	6,07	6,18
G_GI_EG@MT15_Day6	2	7,2500	,02828	,02000	6,9959	7,5041	7,23	7,27
Total	24	5,9125	1,34989	,27555	5,3425	6,4825	4,10	8,23

Multiple Comparisons

Dependent Variable: TVC

Tukey HSD

(I) Film_Code	(J) Film_Code	Mean Difference (I-J)	Std. Error	Sig.	95% Confidence Interval Lower Bound	Upper Bound
CONTROL_Day0	CONTROL_Day2	-1,40000*	,12342	<,001	-1,8900	-,9100
	CONTROL_Day4	-2,65000*	,12342	<,001	-3,1400	-2,1600
	CONTROL_Day6	-3,89500*	,12342	<,001	-4,3850	-3,4050
	G_GI_CT@MT10_Day0	,00000	,12342	1,000	-,4900	,4900
	G_GI_CT@MT10_Day2	-1,50500*	,12342	<,001	-1,9950	-1,0150
	G_GI_CT@MT10_Day4	-1,64000*	,12342	<,001	-2,1300	-1,1500
	G_GI_CT@MT10_Day6	-3,52000*	,12342	<,001	-4,0100	-3,0300
	G_GI_EG@MT15_Day0	,00000	,12342	1,000	-,4900	,4900
	G_GI_EG@MT15_Day2	-,56500*	,12342	,019	-1,0550	-,0750
	G_GI_EG@MT15_Day4	-1,88500*	,12342	<,001	-2,3750	-1,3950
	G_GI_EG@MT15_Day6	-3,01000*	,12342	<,001	-3,5000	-2,5200
CONTROL_Day2	CONTROL_Day0	1,40000*	,12342	<,001	,9100	1,8900
	CONTROL_Day4	-1,25000*	,12342	<,001	-1,7400	-,7600
	CONTROL_Day6	-2,49500*	,12342	<,001	-2,9850	-2,0050
	G_GI_CT@MT10_Day0	1,40000*	,12342	<,001	,9100	1,8900
	G_GI_CT@MT10_Day2	-,10500	,12342	,999	-,5950	,3850
	G_GI_CT@MT10_Day4	-,24000	,12342	,718	-,7300	,2500
	G_GI_CT@MT10_Day6	-2,12000*	,12342	<,001	-2,6100	-1,6300
	G_GI_EG@MT15_Day0	1,40000*	,12342	<,001	,9100	1,8900
	G_GI_EG@MT15_Day2	,83500*	,12342	<,001	,3450	1,3250
	G_GI_EG@MT15_Day4	-,48500	,12342	,053	-,9750	,0050
	G_GI_EG@MT15_Day6	-1,61000*	,12342	<,001	-2,1000	-1,1200
CONTROL_Day4	CONTROL_Day0	2,65000*	,12342	<,001	2,1600	3,1400
	CONTROL_Day2	1,25000*	,12342	<,001	,7600	1,7400
	CONTROL_Day6	-1,24500*	,12342	<,001	-1,7350	-,7550
	G_GI_CT@MT10_Day0	2,65000*	,12342	<,001	2,1600	3,1400
	G_GI_CT@MT10_Day2	1,14500*	,12342	<,001	,6550	1,6350
	G_GI_CT@MT10_Day4	1,01000*	,12342	<,001	,5200	1,5000
	G_GI_CT@MT10_Day6	-,87000*	,12342	<,001	-1,3600	-,3800
	G_GI_EG@MT15_Day0	2,65000*	,12342	<,001	2,1600	3,1400
	G_GI_EG@MT15_Day2	2,08500*	,12342	<,001	1,5950	2,5750

	G_GI_EG@MT15_Day4	,76500*	,12342	,002	,2750	1,2550
	G_GI_EG@MT15_Day6	-,36000	,12342	,244	-,8500	,1300
CONTROL_Day6	CONTROL_Day0	3,89500*	,12342	<,001	3,4050	4,3850
	CONTROL_Day2	2,49500*	,12342	<,001	2,0050	2,9850
	CONTROL_Day4	1,24500*	,12342	<,001	,7550	1,7350
	G_GI_CT@MT10_Day0	3,89500*	,12342	<,001	3,4050	4,3850
	G_GI_CT@MT10_Day2	2,39000*	,12342	<,001	1,9000	2,8800
	G_GI_CT@MT10_Day4	2,25500*	,12342	<,001	1,7650	2,7450
	G_GI_CT@MT10_Day6	,37500	,12342	,206	-,1150	,8650
	G_GI_EG@MT15_Day0	3,89500*	,12342	<,001	3,4050	4,3850
	G_GI_EG@MT15_Day2	3,33000*	,12342	<,001	2,8400	3,8200
	G_GI_EG@MT15_Day4	2,01000*	,12342	<,001	1,5200	2,5000
	G_GI_EG@MT15_Day6	,88500*	,12342	<,001	,3950	1,3750
G_GI_CT@MT10_Day0	CONTROL_Day0	,00000	,12342	1,000	-,4900	,4900
	CONTROL_Day2	-1,40000*	,12342	<,001	-1,8900	-,9100
	CONTROL_Day4	-2,65000*	,12342	<,001	-3,1400	-2,1600
	CONTROL_Day6	-3,89500*	,12342	<,001	-4,3850	-3,4050
	G_GI_CT@MT10_Day2	-1,50500*	,12342	<,001	-1,9950	-1,0150
	G_GI_CT@MT10_Day4	-1,64000*	,12342	<,001	-2,1300	-1,1500
	G_GI_CT@MT10_Day6	-3,52000*	,12342	<,001	-4,0100	-3,0300
	G_GI_EG@MT15_Day0	,00000	,12342	1,000	-,4900	,4900
	G_GI_EG@MT15_Day2	-,56500*	,12342	,019	-1,0550	-,0750
	G_GI_EG@MT15_Day4	-1,88500*	,12342	<,001	-2,3750	-1,3950
	G_GI_EG@MT15_Day6	-3,01000*	,12342	<,001	-3,5000	-2,5200
G_GI_CT@MT10_Day2	CONTROL_Day0	1,50500*	,12342	<,001	1,0150	1,9950
	CONTROL_Day2	,10500	,12342	,999	-,3850	,5950
	CONTROL_Day4	-1,14500*	,12342	<,001	-1,6350	-,6550
	CONTROL_Day6	-2,39000*	,12342	<,001	-2,8800	-1,9000
	G_GI_CT@MT10_Day0	1,50500*	,12342	<,001	1,0150	1,9950
	G_GI_CT@MT10_Day4	-,13500	,12342	,989	-,6250	,3550
	G_GI_CT@MT10_Day6	-2,01500*	,12342	<,001	-2,5050	-1,5250
	G_GI_EG@MT15_Day0	1,50500*	,12342	<,001	1,0150	1,9950

	G_GI_EG@MT15_Day2	,94000*	,12342	<,001	,4500	1,4300
	G_GI_EG@MT15_Day4	-,38000	,12342	,194	-,8700	,1100
	G_GI_EG@MT15_Day6	-1,50500*	,12342	<,001	-1,9950	-1,0150
G_GI_CT@MT10_Day4	CONTROL_Day0	1,64000*	,12342	<,001	1,1500	2,1300
	CONTROL_Day2	,24000	,12342	,718	-,2500	,7300
	CONTROL_Day4	-1,01000*	,12342	<,001	-1,5000	-,5200
	CONTROL_Day6	-2,25500*	,12342	<,001	-2,7450	-1,7650
	G_GI_CT@MT10_Day0	1,64000*	,12342	<,001	1,1500	2,1300
	G_GI_CT@MT10_Day2	,13500	,12342	,989	-,3550	,6250
	G_GI_CT@MT10_Day6	-1,88000*	,12342	<,001	-2,3700	-1,3900
	G_GI_EG@MT15_Day0	1,64000*	,12342	<,001	1,1500	2,1300
	G_GI_EG@MT15_Day2	1,07500*	,12342	<,001	,5850	1,5650
	G_GI_EG@MT15_Day4	-,24500	,12342	,696	-,7350	,2450
	G_GI_EG@MT15_Day6	-1,37000*	,12342	<,001	-1,8600	-,8800
G_GI_CT@MT10_Day6	CONTROL_Day0	3,52000*	,12342	<,001	3,0300	4,0100
	CONTROL_Day2	2,12000*	,12342	<,001	1,6300	2,6100
	CONTROL_Day4	,87000*	,12342	<,001	,3800	1,3600
	CONTROL_Day6	-,37500	,12342	,206	-,8650	,1150
	G_GI_CT@MT10_Day0	3,52000*	,12342	<,001	3,0300	4,0100
	G_GI_CT@MT10_Day2	2,01500*	,12342	<,001	1,5250	2,5050
	G_GI_CT@MT10_Day4	1,88000*	,12342	<,001	1,3900	2,3700
	G_GI_EG@MT15_Day0	3,52000*	,12342	<,001	3,0300	4,0100
	G_GI_EG@MT15_Day2	2,95500*	,12342	<,001	2,4650	3,4450
	G_GI_EG@MT15_Day4	1,63500*	,12342	<,001	1,1450	2,1250
	G_GI_EG@MT15_Day6	,51000*	,12342	,039	,0200	1,0000
G_GI_EG@MT15_Day0	CONTROL_Day0	,00000	,12342	1,000	-,4900	,4900
	CONTROL_Day2	-1,40000*	,12342	<,001	-1,8900	-,9100
	CONTROL_Day4	-2,65000*	,12342	<,001	-3,1400	-2,1600
	CONTROL_Day6	-3,89500*	,12342	<,001	-4,3850	-3,4050
	G_GI_CT@MT10_Day0	,00000	,12342	1,000	-,4900	,4900
	G_GI_CT@MT10_Day2	-1,50500*	,12342	<,001	-1,9950	-1,0150
	G_GI_CT@MT10_Day4	-1,64000*	,12342	<,001	-2,1300	-1,1500

	G_GI_CT@MT10_Day6	-3,52000*	,12342	<,001	-4,0100	-3,0300
	G_GI_EG@MT15_Day2	-,56500*	,12342	,019	-1,0550	-,0750
	G_GI_EG@MT15_Day4	-1,88500*	,12342	<,001	-2,3750	-1,3950
	G_GI_EG@MT15_Day6	-3,01000*	,12342	<,001	-3,5000	-2,5200
G_GI_EG@MT15_Day2	CONTROL_Day0	,56500*	,12342	,019	,0750	1,0550
	CONTROL_Day2	-,83500*	,12342	<,001	-1,3250	-,3450
	CONTROL_Day4	-2,08500*	,12342	<,001	-2,5750	-1,5950
	CONTROL_Day6	-3,33000*	,12342	<,001	-3,8200	-2,8400
	G_GI_CT@MT10_Day0	,56500*	,12342	,019	,0750	1,0550
	G_GI_CT@MT10_Day2	-,94000*	,12342	<,001	-1,4300	-,4500
	G_GI_CT@MT10_Day4	-1,07500*	,12342	<,001	-1,5650	-,5850
	G_GI_CT@MT10_Day6	-2,95500*	,12342	<,001	-3,4450	-2,4650
	G_GI_EG@MT15_Day0	,56500*	,12342	,019	,0750	1,0550
	G_GI_EG@MT15_Day4	-1,32000*	,12342	<,001	-1,8100	-,8300
	G_GI_EG@MT15_Day6	-2,44500*	,12342	<,001	-2,9350	-1,9550
G_GI_EG@MT15_Day4	CONTROL_Day0	1,88500*	,12342	<,001	1,3950	2,3750
	CONTROL_Day2	,48500	,12342	,053	-,0050	,9750
	CONTROL_Day4	-,76500*	,12342	,002	-1,2550	-,2750
	CONTROL_Day6	-2,01000*	,12342	<,001	-2,5000	-1,5200
	G_GI_CT@MT10_Day0	1,88500*	,12342	<,001	1,3950	2,3750
	G_GI_CT@MT10_Day2	,38000	,12342	,194	-,1100	,8700
	G_GI_CT@MT10_Day4	,24500	,12342	,696	-,2450	,7350
	G_GI_CT@MT10_Day6	-1,63500*	,12342	<,001	-2,1250	-1,1450
	G_GI_EG@MT15_Day0	1,88500*	,12342	<,001	1,3950	2,3750
	G_GI_EG@MT15_Day2	1,32000*	,12342	<,001	,8300	1,8100
	G_GI_EG@MT15_Day6	-1,12500*	,12342	<,001	-1,6150	-,6350
G_GI_EG@MT15_Day6	CONTROL_Day0	3,01000*	,12342	<,001	2,5200	3,5000
	CONTROL_Day2	1,61000*	,12342	<,001	1,1200	2,1000
	CONTROL_Day4	,36000	,12342	,244	-,1300	,8500
	CONTROL_Day6	-,88500*	,12342	<,001	-1,3750	-,3950
	G_GI_CT@MT10_Day0	3,01000*	,12342	<,001	2,5200	3,5000
	G_GI_CT@MT10_Day2	1,50500*	,12342	<,001	1,0150	1,9950

	G_GI_CT@MT10_Day4	1,37000*	,12342	<,001	,8800	1,8600
	G_GI_CT@MT10_Day6	-,51000*	,12342	,039	-1,0000	-,0200
	G_GI_EG@MT15_Day0	3,01000*	,12342	<,001	2,5200	3,5000
	G_GI_EG@MT15_Day2	2,44500*	,12342	<,001	1,9550	2,9350
	G_GI_EG@MT15_Day4	1,12500*	,12342	<,001	,6350	1,6150

*. The mean difference is significant at the 0.05 level.

Descriptives

TVC

					95% Confidence Interval for Mean		Minimum	Maximum
	N	Mean	Std. Deviation	Std. Error	Lower Bound	Upper Bound		
CONTROL_Day0	2	4,2400	,19799	,14000	2,4611	6,0189	4,10	4,38
CONTROL_Day2	2	5,6400	,11314	,08000	4,6235	6,6565	5,56	5,72
CONTROL_Day4	2	6,8900	,07071	,05000	6,2547	7,5253	6,84	6,94
CONTROL_Day6	2	8,1350	,13435	,09500	6,9279	9,3421	8,04	8,23
G_GI_CT@MT10_Day0	2	4,2400	,19799	,14000	2,4611	6,0189	4,10	4,38
G_GI_CT@MT10_Day2	2	5,7450	,12021	,08500	4,6650	6,8250	5,66	5,83
G_GI_CT@MT10_Day4	2	5,8800	,04243	,03000	5,4988	6,2612	5,85	5,91
G_GI_CT@MT10_Day6	2	7,7600	,07071	,05000	7,1247	8,3953	7,71	7,81
G_GI_EG@MT15_Day0	2	4,2400	,19799	,14000	2,4611	6,0189	4,10	4,38
G_GI_EG@MT15_Day2	2	4,8050	,03536	,02500	4,4873	5,1227	4,78	4,83
G_GI_EG@MT15_Day4	2	6,1250	,07778	,05500	5,4262	6,8238	6,07	6,18
G_GI_EG@MT15_Day6	2	7,2500	,02828	,02000	6,9959	7,5041	7,23	7,27
Total	24	5,9125	1,34989	,27555	5,3425	6,4825	4,10	8,23

Multiple Comparisons

Dependent Variable: TVC

Tukey HSD

(I) Film_Code	(J) Film_Code	Mean Difference (I-J)	Std. Error	Sig.	95% [illegible]
					Lowe[illegible]
CONTROL_Day0	CONTROL_Day2	-1,40000*	,12342	<,001	-1,89[illegible]
	CONTROL_Day4	-2,65000*	,12342	<,001	-3,14[illegible]
	CONTROL_Day6	-3,89500*	,12342	<,001	-4,38[illegible]
	G_GI_CT@MT10_Day0	,00000	,12342	1,000	-,490[illegible]
	G_GI_CT@MT10_Day2	-1,50500*	,12342	<,001	-1,99[illegible]
	G_GI_CT@MT10_Day4	-1,64000*	,12342	<,001	-2,13[illegible]
	G_GI_CT@MT10_Day6	-3,52000*	,12342	<,001	-4,01[illegible]
	G_GI_EG@MT15_Day0	,00000	,12342	1,000	-,490[illegible]
	G_GI_EG@MT15_Day2	-,56500*	,12342	,019	-1,05[illegible]
	G_GI_EG@MT15_Day4	-1,88500*	,12342	<,001	-2,37[illegible]
	G_GI_EG@MT15_Day6	-3,01000*	,12342	<,001	-3,50[illegible]
CONTROL_Day2	CONTROL_Day0	1,40000*	,12342	<,001	,9100[illegible]
	CONTROL_Day4	-1,25000*	,12342	<,001	-1,74[illegible]
	CONTROL_Day6	-2,49500*	,12342	<,001	-2,98[illegible]
	G_GI_CT@MT10_Day0	1,40000*	,12342	<,001	,9100[illegible]
	G_GI_CT@MT10_Day2	-,10500	,12342	,999	-,595[illegible]
	G_GI_CT@MT10_Day4	-,24000	,12342	,718	-,730[illegible]
	G_GI_CT@MT10_Day6	-2,12000*	,12342	<,001	-2,61[illegible]
	G_GI_EG@MT15_Day0	1,40000*	,12342	<,001	,9100[illegible]
	G_GI_EG@MT15_Day2	,83500*	,12342	<,001	,3450[illegible]
	G_GI_EG@MT15_Day4	-,48500	,12342	,053	-,975[illegible]
	G_GI_EG@MT15_Day6	-1,61000*	,12342	<,001	-2,10[illegible]
CONTROL_Day4	CONTROL_Day0	2,65000*	,12342	<,001	2,160[illegible]
	CONTROL_Day2	1,25000*	,12342	<,001	,7600[illegible]
	CONTROL_Day6	-1,24500*	,12342	<,001	-1,73[illegible]

	G_GI_CT@MT10_Day0	2,65000*	,12342	<,001	2,1600
	G_GI_CT@MT10_Day2	1,14500*	,12342	<,001	,6550
	G_GI_CT@MT10_Day4	1,01000*	,12342	<,001	,5200
	G_GI_CT@MT10_Day6	-,87000*	,12342	<,001	-1,3600
	G_GI_EG@MT15_Day0	2,65000*	,12342	<,001	2,1600
	G_GI_EG@MT15_Day2	2,08500*	,12342	<,001	1,5950
	G_GI_EG@MT15_Day4	,76500*	,12342	,002	,2750
	G_GI_EG@MT15_Day6	-,36000	,12342	,244	-,8500
CONTROL_Day6	CONTROL_Day0	3,89500*	,12342	<,001	3,4050
	CONTROL_Day2	2,49500*	,12342	<,001	2,0050
	CONTROL_Day4	1,24500*	,12342	<,001	,7550
	G_GI_CT@MT10_Day0	3,89500*	,12342	<,001	3,4050
	G_GI_CT@MT10_Day2	2,39000*	,12342	<,001	1,9000
	G_GI_CT@MT10_Day4	2,25500*	,12342	<,001	1,7650
	G_GI_CT@MT10_Day6	,37500	,12342	,206	-,1150
	G_GI_EG@MT15_Day0	3,89500*	,12342	<,001	3,4050
	G_GI_EG@MT15_Day2	3,33000*	,12342	<,001	2,8400
	G_GI_EG@MT15_Day4	2,01000*	,12342	<,001	1,5200
	G_GI_EG@MT15_Day6	,88500*	,12342	<,001	,3950
G_GI_CT@MT10_Day0	CONTROL_Day0	,00000	,12342	1,000	-,4900
	CONTROL_Day2	-1,40000*	,12342	<,001	-1,8900
	CONTROL_Day4	-2,65000*	,12342	<,001	-3,1400
	CONTROL_Day6	-3,89500*	,12342	<,001	-4,3850
	G_GI_CT@MT10_Day2	-1,50500*	,12342	<,001	-1,9950
	G_GI_CT@MT10_Day4	-1,64000*	,12342	<,001	-2,1300
	G_GI_CT@MT10_Day6	-3,52000*	,12342	<,001	-4,0100
	G_GI_EG@MT15_Day0	,00000	,12342	1,000	-,4900
	G_GI_EG@MT15_Day2	-,56500*	,12342	,019	-1,0550
	G_GI_EG@MT15_Day4	-1,88500*	,12342	<,001	-2,3750
	G_GI_EG@MT15_Day6	-3,01000*	,12342	<,001	-3,5000
G_GI_CT@MT10_Day2	CONTROL_Day0	1,50500*	,12342	<,001	1,0150
	CONTROL_Day2	,10500	,12342	,999	-,3850

	CONTROL_Day4	-1,14500*	,12342	<,001	-1,6350
	CONTROL_Day6	-2,39000*	,12342	<,001	-2,8800
	G_GI_CT@MT10_Day0	1,50500*	,12342	<,001	1,0150
	G_GI_CT@MT10_Day4	-,13500	,12342	,989	-,6250
	G_GI_CT@MT10_Day6	-2,01500*	,12342	<,001	-2,5050
	G_GI_EG@MT15_Day0	1,50500*	,12342	<,001	1,0150
	G_GI_EG@MT15_Day2	,94000*	,12342	<,001	,4500
	G_GI_EG@MT15_Day4	-,38000	,12342	,194	-,8700
	G_GI_EG@MT15_Day6	-1,50500*	,12342	<,001	-1,9950
G_GI_CT@MT10_Day4	CONTROL_Day0	1,64000*	,12342	<,001	1,1500
	CONTROL_Day2	,24000	,12342	,718	-,2500
	CONTROL_Day4	-1,01000*	,12342	<,001	-1,5000
	CONTROL_Day6	-2,25500*	,12342	<,001	-2,7450
	G_GI_CT@MT10_Day0	1,64000*	,12342	<,001	1,1500
	G_GI_CT@MT10_Day2	,13500	,12342	,989	-,3550
	G_GI_CT@MT10_Day6	-1,88000*	,12342	<,001	-2,3700
	G_GI_EG@MT15_Day0	1,64000*	,12342	<,001	1,1500
	G_GI_EG@MT15_Day2	1,07500*	,12342	<,001	,5850
	G_GI_EG@MT15_Day4	-,24500	,12342	,696	-,7350
	G_GI_EG@MT15_Day6	-1,37000*	,12342	<,001	-1,8600
G_GI_CT@MT10_Day6	CONTROL_Day0	3,52000*	,12342	<,001	3,0300
	CONTROL_Day2	2,12000*	,12342	<,001	1,6300
	CONTROL_Day4	,87000*	,12342	<,001	,3800
	CONTROL_Day6	-,37500	,12342	,206	-,8650
	G_GI_CT@MT10_Day0	3,52000*	,12342	<,001	3,0300
	G_GI_CT@MT10_Day2	2,01500*	,12342	<,001	1,5250
	G_GI_CT@MT10_Day4	1,88000*	,12342	<,001	1,3900
	G_GI_EG@MT15_Day0	3,52000*	,12342	<,001	3,0300
	G_GI_EG@MT15_Day2	2,95500*	,12342	<,001	2,4650
	G_GI_EG@MT15_Day4	1,63500*	,12342	<,001	1,1450
	G_GI_EG@MT15_Day6	,51000*	,12342	,039	,0200
G_GI_EG@MT15_Day0	CONTROL_Day0	,00000	,12342	1,000	-,4900

	CONTROL_Day2	-1,40000*	,12342	<,001	-1,8900
	CONTROL_Day4	-2,65000*	,12342	<,001	-3,1400
	CONTROL_Day6	-3,89500*	,12342	<,001	-4,3850
	G_GI_CT@MT10_Day0	,00000	,12342	1,000	-,4900
	G_GI_CT@MT10_Day2	-1,50500*	,12342	<,001	-1,9950
	G_GI_CT@MT10_Day4	-1,64000*	,12342	<,001	-2,1300
	G_GI_CT@MT10_Day6	-3,52000*	,12342	<,001	-4,0100
	G_GI_EG@MT15_Day2	-,56500*	,12342	,019	-1,0550
	G_GI_EG@MT15_Day4	-1,88500*	,12342	<,001	-2,3750
	G_GI_EG@MT15_Day6	-3,01000*	,12342	<,001	-3,5000
G_GI_EG@MT15_Day2	CONTROL_Day0	,56500*	,12342	,019	,0750
	CONTROL_Day2	-,83500*	,12342	<,001	-1,3250
	CONTROL_Day4	-2,08500*	,12342	<,001	-2,5750
	CONTROL_Day6	-3,33000*	,12342	<,001	-3,8200
	G_GI_CT@MT10_Day0	,56500*	,12342	,019	,0750
	G_GI_CT@MT10_Day2	-,94000*	,12342	<,001	-1,4300
	G_GI_CT@MT10_Day4	-1,07500*	,12342	<,001	-1,5650
	G_GI_CT@MT10_Day6	-2,95500*	,12342	<,001	-3,4450
	G_GI_EG@MT15_Day0	,56500*	,12342	,019	,0750
	G_GI_EG@MT15_Day4	-1,32000*	,12342	<,001	-1,8100
	G_GI_EG@MT15_Day6	-2,44500*	,12342	<,001	-2,9350
G_GI_EG@MT15_Day4	CONTROL_Day0	1,88500*	,12342	<,001	1,3950
	CONTROL_Day2	,48500	,12342	,053	-,0050
	CONTROL_Day4	-,76500*	,12342	,002	-1,2550
	CONTROL_Day6	-2,01000*	,12342	<,001	-2,5000
	G_GI_CT@MT10_Day0	1,88500*	,12342	<,001	1,3950
	G_GI_CT@MT10_Day2	,38000	,12342	,194	-,1100
	G_GI_CT@MT10_Day4	,24500	,12342	,696	-,2450
	G_GI_CT@MT10_Day6	-1,63500*	,12342	<,001	-2,1250
	G_GI_EG@MT15_Day0	1,88500*	,12342	<,001	1,3950
	G_GI_EG@MT15_Day2	1,32000*	,12342	<,001	,8300
	G_GI_EG@MT15_Day6	-1,12500*	,12342	<,001	-1,6150

G_GI_EG@MT15_Day6	CONTROL_Day0	3,01000*	,12342	<,001	2,5200
	CONTROL_Day2	1,61000*	,12342	<,001	1,1200
	CONTROL_Day4	,36000	,12342	,244	-,1300
	CONTROL_Day6	-,88500*	,12342	<,001	-1,3750
	G_GI_CT@MT10_Day0	3,01000*	,12342	<,001	2,5200
	G_GI_CT@MT10_Day2	1,50500*	,12342	<,001	1,0150
	G_GI_CT@MT10_Day4	1,37000*	,12342	<,001	,8800
	G_GI_CT@MT10_Day6	-,51000*	,12342	,039	-1,0000
	G_GI_EG@MT15_Day0	3,01000*	,12342	<,001	2,5200
	G_GI_EG@MT15_Day2	2,44500*	,12342	<,001	1,9550
	G_GI_EG@MT15_Day4	1,12500*	,12342	<,001	,6350

*. The mean difference is significant at the 0.05 level.

Table S6. Statistical analysis results of pH

Descriptives

pH

					95% Confidence Interva
	N	Mean	Std. Deviation	Std. Error	Lower Bound
CONTROL_Day0	3	5,6467	,03055	,01764	5,5708
CONTROL_Day1	3	5,6567	,02082	,01202	5,6050
CONTROL_Day2	3	5,6700	,02646	,01528	5,6043
CONTROL_Day3	3	5,5467	,02082	,01202	5,4950
G_GI_CT@MT10_Day0	3	5,6467	,03055	,01764	5,5708
G_GI_CT@MT10_Day1	3	5,5733	,00577	,00333	5,5590
G_GI_CT@MT10_Day2	3	5,5167	,01155	,00667	5,4880
G_GI_CT@MT10_Day3	3	5,4333	,04041	,02333	5,3329
G_GI_EG@MT15_Day0	3	5,6467	,03055	,01764	5,5708
G_GI_EG@MT15_Day1	3	5,6133	,04041	,02333	5,5129
G_GI_EG@MT15_Day2	3	5,5767	,00577	,00333	5,5623
G_GI_EG@MT15_Day3	3	5,5800	,01000	,00577	5,5552

Total	36	5,5922	,07088	,01181	5,5682	5,

Multiple Comparisons

Dependent Variable: pH

Tukey HSD

					95% (
(I) Film_Code	(J) Film_Code	Mean Difference (I-J)	Std. Error	Sig.	Lowe
CONTROL_Day0	CONTROL_Day1	-,01000	,02099	1,000	-,085
	CONTROL_Day2	-,02333	,02099	,991	-,099
	CONTROL_Day3	,10000*	,02099	,003	,0243
	G_GI_CT@MT10_Day0	,00000	,02099	1,000	-,075
	G_GI_CT@MT10_Day1	,07333	,02099	,064	-,002
	G_GI_CT@MT10_Day2	,13000*	,02099	<,001	,0543
	G_GI_CT@MT10_Day3	,21333*	,02099	<,001	,1376
	G_GI_EG@MT15_Day0	,00000	,02099	1,000	-,075
	G_GI_EG@MT15_Day1	,03333	,02099	,897	-,042
	G_GI_EG@MT15_Day2	,07000	,02099	,088	-,005
	G_GI_EG@MT15_Day3	,06667	,02099	,121	-,009
CONTROL_Day1	CONTROL_Day0	,01000	,02099	1,000	-,065
	CONTROL_Day2	-,01333	,02099	1,000	-,089
	CONTROL_Day3	,11000*	,02099	,001	,0343
	G_GI_CT@MT10_Day0	,01000	,02099	1,000	-,065
	G_GI_CT@MT10_Day1	,08333*	,02099	,022	,0076
	G_GI_CT@MT10_Day2	,14000*	,02099	<,001	,0643
	G_GI_CT@MT10_Day3	,22333*	,02099	<,001	,1476
	G_GI_EG@MT15_Day0	,01000	,02099	1,000	-,065
	G_GI_EG@MT15_Day1	,04333	,02099	,650	-,032
	G_GI_EG@MT15_Day2	,08000*	,02099	,032	,0043
	G_GI_EG@MT15_Day3	,07667*	,02099	,045	,0010

CONTROL_Day2	CONTROL_Day0	,02333	,02099	,991	-,0524
	CONTROL_Day1	,01333	,02099	1,000	-,0624
	CONTROL_Day3	,12333*	,02099	<,001	,0476
	G_GI_CT@MT10_Day0	,02333	,02099	,991	-,0524
	G_GI_CT@MT10_Day1	,09667*	,02099	,005	,0210
	G_GI_CT@MT10_Day2	,15333*	,02099	<,001	,0776
	G_GI_CT@MT10_Day3	,23667*	,02099	<,001	,1610
	G_GI_EG@MT15_Day0	,02333	,02099	,991	-,0524
	G_GI_EG@MT15_Day1	,05667	,02099	,284	-,0190
	G_GI_EG@MT15_Day2	,09333*	,02099	,007	,0176
	G_GI_EG@MT15_Day3	,09000*	,02099	,011	,0143
CONTROL_Day3	CONTROL_Day0	-,10000*	,02099	,003	-,1757
	CONTROL_Day1	-,11000*	,02099	,001	-,1857
	CONTROL_Day2	-,12333*	,02099	<,001	-,1990
	G_GI_CT@MT10_Day0	-,10000*	,02099	,003	-,1757
	G_GI_CT@MT10_Day1	-,02667	,02099	,976	-,1024
	G_GI_CT@MT10_Day2	,03000	,02099	,946	-,0457
	G_GI_CT@MT10_Day3	,11333*	,02099	<,001	,0376
	G_GI_EG@MT15_Day0	-,10000*	,02099	,003	-,1757
	G_GI_EG@MT15_Day1	-,06667	,02099	,121	-,1424
	G_GI_EG@MT15_Day2	-,03000	,02099	,946	-,1057
	G_GI_EG@MT15_Day3	-,03333	,02099	,897	-,1090
G_GI_CT@MT10_Day0	CONTROL_Day0	,00000	,02099	1,000	-,0757
	CONTROL_Day1	-,01000	,02099	1,000	-,0857
	CONTROL_Day2	-,02333	,02099	,991	-,0990
	CONTROL_Day3	,10000*	,02099	,003	,0243
	G_GI_CT@MT10_Day1	,07333	,02099	,064	-,0024
	G_GI_CT@MT10_Day2	,13000*	,02099	<,001	,0543
	G_GI_CT@MT10_Day3	,21333*	,02099	<,001	,1376
	G_GI_EG@MT15_Day0	,00000	,02099	1,000	-,0757
	G_GI_EG@MT15_Day1	,03333	,02099	,897	-,0424
	G_GI_EG@MT15_Day2	,07000	,02099	,088	-,0057

	G_GI_EG@MT15_Day3	,06667	,02099	,121	-,0090
G_GI_CT@MT10_Day1	CONTROL_Day0	-,07333	,02099	,064	-,1490
	CONTROL_Day1	-,08333*	,02099	,022	-,1590
	CONTROL_Day2	-,09667*	,02099	,005	-,1724
	CONTROL_Day3	,02667	,02099	,976	-,0490
	G_GI_CT@MT10_Day0	-,07333	,02099	,064	-,1490
	G_GI_CT@MT10_Day2	,05667	,02099	,284	-,0190
	G_GI_CT@MT10_Day3	,14000*	,02099	<,001	,0643
	G_GI_EG@MT15_Day0	-,07333	,02099	,064	-,1490
	G_GI_EG@MT15_Day1	-,04000	,02099	,745	-,1157
	G_GI_EG@MT15_Day2	-,00333	,02099	1,000	-,0790
	G_GI_EG@MT15_Day3	-,00667	,02099	1,000	-,0824
G_GI_CT@MT10_Day2	CONTROL_Day0	-,13000*	,02099	<,001	-,2057
	CONTROL_Day1	-,14000*	,02099	<,001	-,2157
	CONTROL_Day2	-,15333*	,02099	<,001	-,2290
	CONTROL_Day3	-,03000	,02099	,946	-,1057
	G_GI_CT@MT10_Day0	-,13000*	,02099	<,001	-,2057
	G_GI_CT@MT10_Day1	-,05667	,02099	,284	-,1324
	G_GI_CT@MT10_Day3	,08333*	,02099	,022	,0076
	G_GI_EG@MT15_Day0	-,13000*	,02099	<,001	-,2057
	G_GI_EG@MT15_Day1	-,09667*	,02099	,005	-,1724
	G_GI_EG@MT15_Day2	-,06000	,02099	,218	-,1357
	G_GI_EG@MT15_Day3	-,06333	,02099	,164	-,1390
G_GI_CT@MT10_Day3	CONTROL_Day0	-,21333*	,02099	<,001	-,2890
	CONTROL_Day1	-,22333*	,02099	<,001	-,2990
	CONTROL_Day2	-,23667*	,02099	<,001	-,3124
	CONTROL_Day3	-,11333*	,02099	<,001	-,1890
	G_GI_CT@MT10_Day0	-,21333*	,02099	<,001	-,2890
	G_GI_CT@MT10_Day1	-,14000*	,02099	<,001	-,2157
	G_GI_CT@MT10_Day2	-,08333*	,02099	,022	-,1590
	G_GI_EG@MT15_Day0	-,21333*	,02099	<,001	-,2890
	G_GI_EG@MT15_Day1	-,18000*	,02099	<,001	-,2557

	G_GI_EG@MT15_Day2	-,14333*	,02099	<,001	-,2190
	G_GI_EG@MT15_Day3	-,14667*	,02099	<,001	-,2224
G_GI_EG@MT15_Day0	CONTROL_Day0	,00000	,02099	1,000	-,0757
	CONTROL_Day1	-,01000	,02099	1,000	-,0857
	CONTROL_Day2	-,02333	,02099	,991	-,0990
	CONTROL_Day3	,10000*	,02099	,003	,0243
	G_GI_CT@MT10_Day0	,00000	,02099	1,000	-,0757
	G_GI_CT@MT10_Day1	,07333	,02099	,064	-,0024
	G_GI_CT@MT10_Day2	,13000*	,02099	<,001	,0543
	G_GI_CT@MT10_Day3	,21333*	,02099	<,001	,1376
	G_GI_EG@MT15_Day1	,03333	,02099	,897	-,0424
	G_GI_EG@MT15_Day2	,07000	,02099	,088	-,0057
	G_GI_EG@MT15_Day3	,06667	,02099	,121	-,0090
G_GI_EG@MT15_Day1	CONTROL_Day0	-,03333	,02099	,897	-,1090
	CONTROL_Day1	-,04333	,02099	,650	-,1190
	CONTROL_Day2	-,05667	,02099	,284	-,1324
	CONTROL_Day3	,06667	,02099	,121	-,0090
	G_GI_CT@MT10_Day0	-,03333	,02099	,897	-,1090
	G_GI_CT@MT10_Day1	,04000	,02099	,745	-,0357
	G_GI_CT@MT10_Day2	,09667*	,02099	,005	,0210
	G_GI_CT@MT10_Day3	,18000*	,02099	<,001	,1043
	G_GI_EG@MT15_Day0	-,03333	,02099	,897	-,1090
	G_GI_EG@MT15_Day2	,03667	,02099	,830	-,0390
	G_GI_EG@MT15_Day3	,03333	,02099	,897	-,0424
G_GI_EG@MT15_Day2	CONTROL_Day0	-,07000	,02099	,088	-,1457
	CONTROL_Day1	-,08000*	,02099	,032	-,1557
	CONTROL_Day2	-,09333*	,02099	,007	-,1690
	CONTROL_Day3	,03000	,02099	,946	-,0457
	G_GI_CT@MT10_Day0	-,07000	,02099	,088	-,1457
	G_GI_CT@MT10_Day1	,00333	,02099	1,000	-,0724
	G_GI_CT@MT10_Day2	,06000	,02099	,218	-,0157
	G_GI_CT@MT10_Day3	,14333*	,02099	<,001	,0676

	G_GI_EG@MT15_Day0	-,07000	,02099	,088	-,1457
	G_GI_EG@MT15_Day1	-,03667	,02099	,830	-,1124
	G_GI_EG@MT15_Day3	-,00333	,02099	1,000	-,0790
G_GI_EG@MT15_Day3	CONTROL_Day0	-,06667	,02099	,121	-,1424
	CONTROL_Day1	-,07667*	,02099	,045	-,1524
	CONTROL_Day2	-,09000*	,02099	,011	-,1657
	CONTROL_Day3	,03333	,02099	,897	-,0424
	G_GI_CT@MT10_Day0	-,06667	,02099	,121	-,1424
	G_GI_CT@MT10_Day1	,00667	,02099	1,000	-,0690
	G_GI_CT@MT10_Day2	,06333	,02099	,164	-,0124
	G_GI_CT@MT10_Day3	,14667*	,02099	<,001	,0710
	G_GI_EG@MT15_Day0	-,06667	,02099	,121	-,1424
	G_GI_EG@MT15_Day1	-,03333	,02099	,897	-,1090
	G_GI_EG@MT15_Day2	,00333	,02099	1,000	-,0724

*. The mean difference is significant at the 0.05 level.

Table S7. Statistical analysis results of Lab

Tukey HSD

Dependent Variable	(I) Treatment Goup	(J) Treatment Goup	Mean Difference (I-J)	Std. Error	Sig.	95%[illegible] Low[illegible]
$\Delta L = L - L_0$ (Day 0 reference)	Control	Citral	-1,5738*	,20439	<,001	-2,0[illegible]
		Eugenol	-2,3220*	,20439	<,001	-2,8[illegible]
	Citral	Control	1,5738*	,20439	<,001	1,06[illegible]
		Eugenol	-,7482*	,20439	,003	-1,2[illegible]
	Eugenol	Control	2,3220*	,20439	<,001	1,81[illegible]
		Citral	,7482*	,20439	,003	,237[illegible]
$\Delta a = a - a_0$	Control	Citral	-2,1734*	,12072	<,001	-2,4[illegible]
		Eugenol	-3,0512*	,12072	<,001	-3,3[illegible]

	Citral	Control	2,1734*	,12072	<,001	1,8719
		Eugenol	-,8778*	,12072	<,001	-1,1793
	Eugenol	Control	3,0512*	,12072	<,001	2,7498
		Citral	,8778*	,12072	<,001	,5763
$\Delta b = b - b_0$	Control	Citral	1,3246*	,11124	<,001	1,0468
		Eugenol	1,3488*	,11124	<,001	1,0710
	Citral	Control	-1,3246*	,11124	<,001	-1,6024
		Eugenol	,0242	,11124	,974	-,2537
	Eugenol	Control	-1,3488*	,11124	<,001	-1,6266
		Citral	-,0242	,11124	,974	-,3020
Total color difference ΔE	Control	Citral	3,0731*	,12864	<,001	2,7519
		Eugenol	4,2881*	,12864	<,001	3,9669
	Citral	Control	-3,0731*	,12864	<,001	-3,3944
		Eugenol	1,2150*	,12864	<,001	,8938
	Eugenol	Control	-4,2881*	,12864	<,001	-4,6094
		Citral	-1,2150*	,12864	<,001	-1,5362

Based on observed means.

The error term is Mean Square(Error) = ,099.

*. The mean difference is significant at the ,05 level.

Multiple Comparisons

Tukey HSD

Dependent Variable	(I) Storage Day (0 = Day 0, 2 = Day 2, etc.)	(J) Storage Day (0 = Day 0, 2 = Day 2, etc.)	Mean Difference (I-J)	Std. Error	Sig.	95% Confidence Interva Lower Bound	Upper Boun
$\Delta L = L - L_0$ (Day 0 reference)	,00	2,00	1,3595*	,23601	<,001	,7084	2,0105
		4,00	2,6499*	,23601	<,001	1,9988	3,3009
		6,00	4,6757*	,23601	<,001	4,0246	5,3267

	2,00	,00	-1,3595*	,23601	<,001	-2,0105	-,7084
		4,00	1,2904*	,23601	<,001	,6393	1,9414
		6,00	3,3162*	,23601	<,001	2,6652	3,9673
	4,00	,00	-2,6499*	,23601	<,001	-3,3009	-1,9988
		2,00	-1,2904*	,23601	<,001	-1,9414	-,6393
		6,00	2,0258*	,23601	<,001	1,3748	2,6769
	6,00	,00	-4,6757*	,23601	<,001	-5,3267	-4,0246
		2,00	-3,3162*	,23601	<,001	-3,9673	-2,6652
		4,00	-2,0258*	,23601	<,001	-2,6769	-1,3748
$\Delta a = a - a_0$	,00	2,00	1,7366*	,13940	<,001	1,3520	2,1211
		4,00	3,8272*	,13940	<,001	3,4427	4,2118
		6,00	5,5600*	,13940	<,001	5,1755	5,9445
	2,00	,00	-1,7366*	,13940	<,001	-2,1211	-1,3520
		4,00	2,0906*	,13940	<,001	1,7061	2,4752
		6,00	3,8234*	,13940	<,001	3,4389	4,2080
	4,00	,00	-3,8272*	,13940	<,001	-4,2118	-3,4427
		2,00	-2,0906*	,13940	<,001	-2,4752	-1,7061
		6,00	1,7328*	,13940	<,001	1,3483	2,1173
	6,00	,00	-5,5600*	,13940	<,001	-5,9445	-5,1755
		2,00	-3,8234*	,13940	<,001	-4,2080	-3,4389
		4,00	-1,7328*	,13940	<,001	-2,1173	-1,3483
$\Delta b = b - b_0$	,00	2,00	-,7951*	,12845	<,001	-1,1494	-,4407
		4,00	-1,1814*	,12845	<,001	-1,5358	-,8271
		6,00	-1,7683*	,12845	<,001	-2,1227	-1,4140
	2,00	,00	,7951*	,12845	<,001	,4407	1,1494
		4,00	-,3864*	,12845	,029	-,7407	-,0320
		6,00	-,9733*	,12845	<,001	-1,3276	-,6189
	4,00	,00	1,1814*	,12845	<,001	,8271	1,5358
		2,00	,3864*	,12845	,029	,0320	,7407
		6,00	-,5869*	,12845	<,001	-,9412	-,2325

	6,00	,00	1,7683*	,12845	<,001	1,4140	2,1227
		2,00	,9733*	,12845	<,001	,6189	1,3276
		4,00	,5869*	,12845	<,001	,2325	,9412
Total color difference ΔE	,00	2,00	-1,4898*	,14854	<,001	-1,8996	-1,0800
		4,00	-3,8819*	,14854	<,001	-4,2917	-3,4722
		6,00	-6,5570*	,14854	<,001	-6,9668	-6,1473
	2,00	,00	1,4898*	,14854	<,001	1,0800	1,8996
		4,00	-2,3922*	,14854	<,001	-2,8019	-1,9824
		6,00	-5,0672*	,14854	<,001	-5,4770	-4,6575
	4,00	,00	3,8819*	,14854	<,001	3,4722	4,2917
		2,00	2,3922*	,14854	<,001	1,9824	2,8019
		6,00	-2,6751*	,14854	<,001	-3,0848	-2,2653
	6,00	,00	6,5570*	,14854	<,001	6,1473	6,9668
		2,00	5,0672*	,14854	<,001	4,6575	5,4770
		4,00	2,6751*	,14854	<,001	2,2653	3,0848

Based on observed means.

The error term is Mean Square(Error) = ,099.

*. The mean difference is significant at the ,05 level.

Table S8. Statistical analysis results of sensory analysis

Descriptives

		N	Mean	Std. Deviation	Std. Error	95% Confidence Interval for Mean		Minimum	Maximum
						Lower Bound	Upper Bound		
Color	CONTROL_Day0	4	5,0000	,00000	,00000	5,0000	5,0000	5,00	5,00
	CONTROL_Day2	4	4,5750	,33040	,16520	4,0493	5,1007	4,20	5,00
	CONTROL_Day4	4	3,9000	,27080	,13540	3,4691	4,3309	3,50	4,10
	CONTROL_Day6	4	2,9750	,33040	,16520	2,4493	3,5007	2,50	3,20
	G_GI_CT@MT10_Day0	4	5,0000	,00000	,00000	5,0000	5,0000	5,00	5,00
	G_GI_CT@MT10_Day2	4	4,7250	,25000	,12500	4,3272	5,1228	4,40	5,00
	G_GI_CT@MT10_Day4	4	4,0750	,22174	,11087	3,7222	4,4278	3,90	4,40
	G_GI_CT@MT10_Day6	4	3,1000	,33665	,16833	2,5643	3,6357	2,70	3,50
	G_GI_EG@MT15_Day0	4	5,0000	,00000	,00000	5,0000	5,0000	5,00	5,00
	G_GI_EG@MT15_Day2	4	4,7000	,21602	,10801	4,3563	5,0437	4,50	5,00
	G_GI_EG@MT15_Day4	4	4,1750	,23629	,11815	3,7990	4,5510	4,00	4,50
	G_GI_EG@MT15_Day6	4	3,5000	,00000	,00000	3,5000	3,5000	3,50	3,50
	Total	48	4,2271	,73506	,10610	4,0136	4,4405	2,50	5,00
Smell	CONTROL_Day0	4	5,0000	,00000	,00000	5,0000	5,0000	5,00	5,00
	CONTROL_Day2	4	4,1750	,53774	,26887	3,3193	5,0307	3,50	4,70
	CONTROL_Day4	4	3,5000	,73485	,36742	2,3307	4,6693	2,50	4,10
	CONTROL_Day6	4	3,0000	,81240	,40620	1,7073	4,2927	2,00	3,80
	G_GI_CT@MT10_Day0	4	5,0000	,00000	,00000	5,0000	5,0000	5,00	5,00
	G_GI_CT@MT10_Day2	4	4,6250	,20616	,10308	4,2970	4,9530	4,40	4,80
	G_GI_CT@MT10_Day4	4	4,0750	,15000	,07500	3,8363	4,3137	4,00	4,30
	G_GI_CT@MT10_Day6	4	3,5500	,40415	,20207	2,9069	4,1931	3,00	3,90
	G_GI_EG@MT15_Day0	4	5,0000	,00000	,00000	5,0000	5,0000	5,00	5,00
	G_GI_EG@MT15_Day2	4	4,8750	,09574	,04787	4,7227	5,0273	4,80	5,00
	G_GI_EG@MT15_Day4	4	4,4500	,44347	,22174	3,7443	5,1557	4,00	5,00
	G_GI_EG@MT15_Day6	4	4,0750	,68981	,34490	2,9774	5,1726	3,50	5,00
	Total	48	4,2771	,75688	,10925	4,0573	4,4969	2,00	5,00
Taste	CONTROL_Day0	4	5,0000	,00000	,00000	5,0000	5,0000	5,00	5,00
	CONTROL_Day2	4	4,7000	,18257	,09129	4,4095	4,9905	4,50	4,90
	CONTROL_Day4	4	3,9750	,34034	,17017	3,4334	4,5166	3,50	4,30
	CONTROL_Day6	4	3,1500	,51962	,25981	2,3232	3,9768	2,50	3,70
	G_GI_CT@MT10_Day0	4	5,0000	,00000	,00000	5,0000	5,0000	5,00	5,00
	G_GI_CT@MT10_Day2	4	4,7500	,23805	,11902	4,3712	5,1288	4,50	5,00
	G_GI_CT@MT10_Day4	4	4,0500	,26458	,13229	3,6290	4,4710	3,70	4,30

	G_GI_CT@MT10_Day6	4	3,4250	,67515	,33758	2,3507	4,4993	2,70	4,00
	G_GI_EG@MT15_Day0	4	5,0000	,00000	,00000	5,0000	5,0000	5,00	5,00
	G_GI_EG@MT15_Day2	4	4,6750	,15000	,07500	4,4363	4,9137	4,50	4,80
	G_GI_EG@MT15_Day4	4	4,2250	,38622	,19311	3,6104	4,8396	4,00	4,80
	G_GI_EG@MT15_Day6	4	3,8750	,66018	,33009	2,8245	4,9255	3,00	4,60
	Total	48	4,3188	,69149	,09981	4,1180	4,5195	2,50	5,00

Multiple Comparisons

Tukey HSD

Dependent Variable	(I) Film_Code	(J) Film_Code	Mean Difference (I-J)	Std. Error	Sig.	95% Confidence Interval	
						Lower Bound	Upper Bound
Color	CONTROL_Day0	CONTROL_Day2	,42500	,16062	,293	-,1356	,9856
		CONTROL_Day4	1,10000*	,16062	<,001	,5394	1,6606
		CONTROL_Day6	2,02500*	,16062	<,001	1,4644	2,5856
		G_GI_CT@MT10_Day0	,00000	,16062	1,000	-,5606	,5606
		G_GI_CT@MT10_Day2	,27500	,16062	,851	-,2856	,8356
		G_GI_CT@MT10_Day4	,92500*	,16062	<,001	,3644	1,4856
		G_GI_CT@MT10_Day6	1,90000*	,16062	<,001	1,3394	2,4606
		G_GI_EG@MT15_Day0	,00000	,16062	1,000	-,5606	,5606
		G_GI_EG@MT15_Day2	,30000	,16062	,770	-,2606	,8606
		G_GI_EG@MT15_Day4	,82500*	,16062	<,001	,2644	1,3856
		G_GI_EG@MT15_Day6	1,50000*	,16062	<,001	,9394	2,0606
	CONTROL_Day2	CONTROL_Day0	-,42500	,16062	,293	-,9856	,1356
		CONTROL_Day4	,67500*	,16062	,008	,1144	1,2356
		CONTROL_Day6	1,60000*	,16062	<,001	1,0394	2,1606
		G_GI_CT@MT10_Day0	-,42500	,16062	,293	-,9856	,1356
		G_GI_CT@MT10_Day2	-,15000	,16062	,998	-,7106	,4106
		G_GI_CT@MT10_Day4	,50000	,16062	,118	-,0606	1,0606
		G_GI_CT@MT10_Day6	1,47500*	,16062	<,001	,9144	2,0356
		G_GI_EG@MT15_Day0	-,42500	,16062	,293	-,9856	,1356
		G_GI_EG@MT15_Day2	-,12500	,16062	1,000	-,6856	,4356
		G_GI_EG@MT15_Day4	,40000	,16062	,377	-,1606	,9606
		G_GI_EG@MT15_Day6	1,07500*	,16062	<,001	,5144	1,6356
	CONTROL_Day4	CONTROL_Day0	-1,10000*	,16062	<,001	-1,6606	-,5394
		CONTROL_Day2	-,67500*	,16062	,008	-1,2356	-,1144
		CONTROL_Day6	,92500*	,16062	<,001	,3644	1,4856
		G_GI_CT@MT10_Day0	-1,10000*	,16062	<,001	-1,6606	-,5394
		G_GI_CT@MT10_Day2	-,82500*	,16062	<,001	-1,3856	-,2644
		G_GI_CT@MT10_Day4	-,17500	,16062	,993	-,7356	,3856
		G_GI_CT@MT10_Day6	,80000*	,16062	<,001	,2394	1,3606
		G_GI_EG@MT15_Day0	-1,10000*	,16062	<,001	-1,6606	-,5394
		G_GI_EG@MT15_Day2	-,80000*	,16062	<,001	-1,3606	-,2394
		G_GI_EG@MT15_Day4	-,27500	,16062	,851	-,8356	,2856

		G_GI_EG@MT15_Day6	,40000	,16062	,377	-,1606	,9606
	CONTROL_Day6	CONTROL_Day0	-2,02500*	,16062	<,001	-2,5856	-1,4644
		CONTROL_Day2	-1,60000*	,16062	<,001	-2,1606	-1,0394
		CONTROL_Day4	-,92500*	,16062	<,001	-1,4856	-,3644
		G_GI_CT@MT10_Day0	-2,02500*	,16062	<,001	-2,5856	-1,4644
		G_GI_CT@MT10_Day2	-1,75000*	,16062	<,001	-2,3106	-1,1894
		G_GI_CT@MT10_Day4	-1,10000*	,16062	<,001	-1,6606	-,5394
		G_GI_CT@MT10_Day6	-,12500	,16062	1,000	-,6856	,4356
		G_GI_EG@MT15_Day0	-2,02500*	,16062	<,001	-2,5856	-1,4644
		G_GI_EG@MT15_Day2	-1,72500*	,16062	<,001	-2,2856	-1,1644
		G_GI_EG@MT15_Day4	-1,20000*	,16062	<,001	-1,7606	-,6394
		G_GI_EG@MT15_Day6	-,52500	,16062	,084	-1,0856	,0356
	G_GI_CT@MT10_Day0	CONTROL_Day0	,00000	,16062	1,000	-,5606	,5606
		CONTROL_Day2	,42500	,16062	,293	-,1356	,9856
		CONTROL_Day4	1,10000*	,16062	<,001	,5394	1,6606
		CONTROL_Day6	2,02500*	,16062	<,001	1,4644	2,5856
		G_GI_CT@MT10_Day2	,27500	,16062	,851	-,2856	,8356
		G_GI_CT@MT10_Day4	,92500*	,16062	<,001	,3644	1,4856
		G_GI_CT@MT10_Day6	1,90000*	,16062	<,001	1,3394	2,4606
		G_GI_EG@MT15_Day0	,00000	,16062	1,000	-,5606	,5606
		G_GI_EG@MT15_Day2	,30000	,16062	,770	-,2606	,8606
		G_GI_EG@MT15_Day4	,82500*	,16062	<,001	,2644	1,3856
		G_GI_EG@MT15_Day6	1,50000*	,16062	<,001	,9394	2,0606
	G_GI_CT@MT10_Day2	CONTROL_Day0	-,27500	,16062	,851	-,8356	,2856
		CONTROL_Day2	,15000	,16062	,998	-,4106	,7106
		CONTROL_Day4	,82500*	,16062	<,001	,2644	1,3856
		CONTROL_Day6	1,75000*	,16062	<,001	1,1894	2,3106
		G_GI_CT@MT10_Day0	-,27500	,16062	,851	-,8356	,2856
		G_GI_CT@MT10_Day4	,65000*	,16062	,012	,0894	1,2106
		G_GI_CT@MT10_Day6	1,62500*	,16062	<,001	1,0644	2,1856
		G_GI_EG@MT15_Day0	-,27500	,16062	,851	-,8356	,2856
		G_GI_EG@MT15_Day2	,02500	,16062	1,000	-,5356	,5856

		G_GI_EG@MT15_Day4	,55000	,16062	,059	-,0106	1,1106
		G_GI_EG@MT15_Day6	1,22500*	,16062	<,001	,6644	1,7856
	G_GI_CT@MT10_Day4	CONTROL_Day0	-,92500*	,16062	<,001	-1,4856	-,3644
		CONTROL_Day2	-,50000	,16062	,118	-1,0606	,0606
		CONTROL_Day4	,17500	,16062	,993	-,3856	,7356
		CONTROL_Day6	1,10000*	,16062	<,001	,5394	1,6606
		G_GI_CT@MT10_Day0	-,92500*	,16062	<,001	-1,4856	-,3644
		G_GI_CT@MT10_Day2	-,65000*	,16062	,012	-1,2106	-,0894
		G_GI_CT@MT10_Day6	,97500*	,16062	<,001	,4144	1,5356
		G_GI_EG@MT15_Day0	-,92500*	,16062	<,001	-1,4856	-,3644
		G_GI_EG@MT15_Day2	-,62500*	,16062	,018	-1,1856	-,0644
		G_GI_EG@MT15_Day4	-,10000	,16062	1,000	-,6606	,4606
		G_GI_EG@MT15_Day6	,57500*	,16062	,040	,0144	1,1356
	G_GI_CT@MT10_Day6	CONTROL_Day0	-1,90000*	,16062	<,001	-2,4606	-1,3394
		CONTROL_Day2	-1,47500*	,16062	<,001	-2,0356	-,9144
		CONTROL_Day4	-,80000*	,16062	<,001	-1,3606	-,2394
		CONTROL_Day6	,12500	,16062	1,000	-,4356	,6856
		G_GI_CT@MT10_Day0	-1,90000*	,16062	<,001	-2,4606	-1,3394
		G_GI_CT@MT10_Day2	-1,62500*	,16062	<,001	-2,1856	-1,0644
		G_GI_CT@MT10_Day4	-,97500*	,16062	<,001	-1,5356	-,4144
		G_GI_EG@MT15_Day0	-1,90000*	,16062	<,001	-2,4606	-1,3394
		G_GI_EG@MT15_Day2	-1,60000*	,16062	<,001	-2,1606	-1,0394
		G_GI_EG@MT15_Day4	-1,07500*	,16062	<,001	-1,6356	-,5144
		G_GI_EG@MT15_Day6	-,40000	,16062	,377	-,9606	,1606
	G_GI_EG@MT15_Day0	CONTROL_Day0	,00000	,16062	1,000	-,5606	,5606
		CONTROL_Day2	,42500	,16062	,293	-,1356	,9856
		CONTROL_Day4	1,10000*	,16062	<,001	,5394	1,6606
		CONTROL_Day6	2,02500*	,16062	<,001	1,4644	2,5856
		G_GI_CT@MT10_Day0	,00000	,16062	1,000	-,5606	,5606
		G_GI_CT@MT10_Day2	,27500	,16062	,851	-,2856	,8356
		G_GI_CT@MT10_Day4	,92500*	,16062	<,001	,3644	1,4856
		G_GI_CT@MT10_Day6	1,90000*	,16062	<,001	1,3394	2,4606

		G_GI_EG@MT15_Day2	,30000	,16062	,770	-,2606	,8606
		G_GI_EG@MT15_Day4	,82500*	,16062	<,001	,2644	1,3856
		G_GI_EG@MT15_Day6	1,50000*	,16062	<,001	,9394	2,0606
	G_GI_EG@MT15_Day2	CONTROL_Day0	-,30000	,16062	,770	-,8606	,2606
		CONTROL_Day2	,12500	,16062	1,000	-,4356	,6856
		CONTROL_Day4	,80000*	,16062	<,001	,2394	1,3606
		CONTROL_Day6	1,72500*	,16062	<,001	1,1644	2,2856
		G_GI_CT@MT10_Day0	-,30000	,16062	,770	-,8606	,2606
		G_GI_CT@MT10_Day2	-,02500	,16062	1,000	-,5856	,5356
		G_GI_CT@MT10_Day4	,62500*	,16062	,018	,0644	1,1856
		G_GI_CT@MT10_Day6	1,60000*	,16062	<,001	1,0394	2,1606
		G_GI_EG@MT15_Day0	-,30000	,16062	,770	-,8606	,2606
		G_GI_EG@MT15_Day4	,52500	,16062	,084	-,0356	1,0856
		G_GI_EG@MT15_Day6	1,20000*	,16062	<,001	,6394	1,7606
	G_GI_EG@MT15_Day4	CONTROL_Day0	-,82500*	,16062	<,001	-1,3856	-,2644
		CONTROL_Day2	-,40000	,16062	,377	-,9606	,1606
		CONTROL_Day4	,27500	,16062	,851	-,2856	,8356
		CONTROL_Day6	1,20000*	,16062	<,001	,6394	1,7606
		G_GI_CT@MT10_Day0	-,82500*	,16062	<,001	-1,3856	-,2644
		G_GI_CT@MT10_Day2	-,55000	,16062	,059	-1,1106	,0106
		G_GI_CT@MT10_Day4	,10000	,16062	1,000	-,4606	,6606
		G_GI_CT@MT10_Day6	1,07500*	,16062	<,001	,5144	1,6356
		G_GI_EG@MT15_Day0	-,82500*	,16062	<,001	-1,3856	-,2644
		G_GI_EG@MT15_Day2	-,52500	,16062	,084	-1,0856	,0356
		G_GI_EG@MT15_Day6	,67500*	,16062	,008	,1144	1,2356
	G_GI_EG@MT15_Day6	CONTROL_Day0	-1,50000*	,16062	<,001	-2,0606	-,9394
		CONTROL_Day2	-1,07500*	,16062	<,001	-1,6356	-,5144
		CONTROL_Day4	-,40000	,16062	,377	-,9606	,1606
		CONTROL_Day6	,52500	,16062	,084	-,0356	1,0856
		G_GI_CT@MT10_Day0	-1,50000*	,16062	<,001	-2,0606	-,9394
		G_GI_CT@MT10_Day2	-1,22500*	,16062	<,001	-1,7856	-,6644
		G_GI_CT@MT10_Day4	-,57500*	,16062	,040	-1,1356	-,0144

		G_GI_CT@MT10_Day6	,40000	,16062	,377	-,1606	,9606
		G_GI_EG@MT15_Day0	-1,50000*	,16062	<,001	-2,0606	-,9394
		G_GI_EG@MT15_Day2	-1,20000*	,16062	<,001	-1,7606	-,6394
		G_GI_EG@MT15_Day4	-,67500*	,16062	,008	-1,2356	-,1144
Smell	CONTROL_Day0	CONTROL_Day2	,82500	,31617	,312	-,2785	1,9285
		CONTROL_Day4	1,50000*	,31617	,002	,3965	2,6035
		CONTROL_Day6	2,00000*	,31617	<,001	,8965	3,1035
		G_GI_CT@MT10_Day0	,00000	,31617	1,000	-1,1035	1,1035
		G_GI_CT@MT10_Day2	,37500	,31617	,987	-,7285	1,4785
		G_GI_CT@MT10_Day4	,92500	,31617	,175	-,1785	2,0285
		G_GI_CT@MT10_Day6	1,45000*	,31617	,003	,3465	2,5535
		G_GI_EG@MT15_Day0	,00000	,31617	1,000	-1,1035	1,1035
		G_GI_EG@MT15_Day2	,12500	,31617	1,000	-,9785	1,2285
		G_GI_EG@MT15_Day4	,55000	,31617	,838	-,5535	1,6535
		G_GI_EG@MT15_Day6	,92500	,31617	,175	-,1785	2,0285
	CONTROL_Day2	CONTROL_Day0	-,82500	,31617	,312	-1,9285	,2785
		CONTROL_Day4	,67500	,31617	,603	-,4285	1,7785
		CONTROL_Day6	1,17500*	,31617	,029	,0715	2,2785
		G_GI_CT@MT10_Day0	-,82500	,31617	,312	-1,9285	,2785
		G_GI_CT@MT10_Day2	-,45000	,31617	,951	-1,5535	,6535
		G_GI_CT@MT10_Day4	,10000	,31617	1,000	-1,0035	1,2035
		G_GI_CT@MT10_Day6	,62500	,31617	,705	-,4785	1,7285
		G_GI_EG@MT15_Day0	-,82500	,31617	,312	-1,9285	,2785
		G_GI_EG@MT15_Day2	-,70000	,31617	,551	-1,8035	,4035
		G_GI_EG@MT15_Day4	-,27500	,31617	,999	-1,3785	,8285
		G_GI_EG@MT15_Day6	,10000	,31617	1,000	-1,0035	1,2035
	CONTROL_Day4	CONTROL_Day0	-1,50000*	,31617	,002	-2,6035	-,3965
		CONTROL_Day2	-,67500	,31617	,603	-1,7785	,4285
		CONTROL_Day6	,50000	,31617	,905	-,6035	1,6035
		G_GI_CT@MT10_Day0	-1,50000*	,31617	,002	-2,6035	-,3965
		G_GI_CT@MT10_Day2	-1,12500*	,31617	,042	-2,2285	-,0215
		G_GI_CT@MT10_Day4	-,57500	,31617	,798	-1,6785	,5285

		G_GI_CT@MT10_Day6	-,05000	,31617	1,000	-1,1535	1,0535
		G_GI_EG@MT15_Day0	-1,50000*	,31617	,002	-2,6035	-,3965
		G_GI_EG@MT15_Day2	-1,37500*	,31617	,005	-2,4785	-,2715
		G_GI_EG@MT15_Day4	-,95000	,31617	,149	-2,0535	,1535
		G_GI_EG@MT15_Day6	-,57500	,31617	,798	-1,6785	,5285
	CONTROL_Day6	CONTROL_Day0	-2,00000*	,31617	<,001	-3,1035	-,8965
		CONTROL_Day2	-1,17500*	,31617	,029	-2,2785	-,0715
		CONTROL_Day4	-,50000	,31617	,905	-1,6035	,6035
		G_GI_CT@MT10_Day0	-2,00000*	,31617	<,001	-3,1035	-,8965
		G_GI_CT@MT10_Day2	-1,62500*	,31617	<,001	-2,7285	-,5215
		G_GI_CT@MT10_Day4	-1,07500	,31617	,062	-2,1785	,0285
		G_GI_CT@MT10_Day6	-,55000	,31617	,838	-1,6535	,5535
		G_GI_EG@MT15_Day0	-2,00000*	,31617	<,001	-3,1035	-,8965
		G_GI_EG@MT15_Day2	-1,87500*	,31617	<,001	-2,9785	-,7715
		G_GI_EG@MT15_Day4	-1,45000*	,31617	,003	-2,5535	-,3465
		G_GI_EG@MT15_Day6	-1,07500	,31617	,062	-2,1785	,0285
	G_GI_CT@MT10_Day0	CONTROL_Day0	,00000	,31617	1,000	-1,1035	1,1035
		CONTROL_Day2	,82500	,31617	,312	-,2785	1,9285
		CONTROL_Day4	1,50000*	,31617	,002	,3965	2,6035
		CONTROL_Day6	2,00000*	,31617	<,001	,8965	3,1035
		G_GI_CT@MT10_Day2	,37500	,31617	,987	-,7285	1,4785
		G_GI_CT@MT10_Day4	,92500	,31617	,175	-,1785	2,0285
		G_GI_CT@MT10_Day6	1,45000*	,31617	,003	,3465	2,5535
		G_GI_EG@MT15_Day0	,00000	,31617	1,000	-1,1035	1,1035
		G_GI_EG@MT15_Day2	,12500	,31617	1,000	-,9785	1,2285
		G_GI_EG@MT15_Day4	,55000	,31617	,838	-,5535	1,6535
		G_GI_EG@MT15_Day6	,92500	,31617	,175	-,1785	2,0285
	G_GI_CT@MT10_Day2	CONTROL_Day0	-,37500	,31617	,987	-1,4785	,7285
		CONTROL_Day2	,45000	,31617	,951	-,6535	1,5535
		CONTROL_Day4	1,12500*	,31617	,042	,0215	2,2285
		CONTROL_Day6	1,62500*	,31617	<,001	,5215	2,7285
		G_GI_CT@MT10_Day0	-,37500	,31617	,987	-1,4785	,7285

		G_GI_CT@MT10_Day4	,55000	,31617	,838	-,5535	1,6535
		G_GI_CT@MT10_Day6	1,07500	,31617	,062	-,0285	2,1785
		G_GI_EG@MT15_Day0	-,37500	,31617	,987	-1,4785	,7285
		G_GI_EG@MT15_Day2	-,25000	,31617	1,000	-1,3535	,8535
		G_GI_EG@MT15_Day4	,17500	,31617	1,000	-,9285	1,2785
		G_GI_EG@MT15_Day6	,55000	,31617	,838	-,5535	1,6535
	G_GI_CT@MT10_Day4	CONTROL_Day0	-,92500	,31617	,175	-2,0285	,1785
		CONTROL_Day2	-,10000	,31617	1,000	-1,2035	1,0035
		CONTROL_Day4	,57500	,31617	,798	-,5285	1,6785
		CONTROL_Day6	1,07500	,31617	,062	-,0285	2,1785
		G_GI_CT@MT10_Day0	-,92500	,31617	,175	-2,0285	,1785
		G_GI_CT@MT10_Day2	-,55000	,31617	,838	-1,6535	,5535
		G_GI_CT@MT10_Day6	,52500	,31617	,874	-,5785	1,6285
		G_GI_EG@MT15_Day0	-,92500	,31617	,175	-2,0285	,1785
		G_GI_EG@MT15_Day2	-,80000	,31617	,355	-1,9035	,3035
		G_GI_EG@MT15_Day4	-,37500	,31617	,987	-1,4785	,7285
		G_GI_EG@MT15_Day6	,00000	,31617	1,000	-1,1035	1,1035
	G_GI_CT@MT10_Day6	CONTROL_Day0	-1,45000*	,31617	,003	-2,5535	-,3465
		CONTROL_Day2	-,62500	,31617	,705	-1,7285	,4785
		CONTROL_Day4	,05000	,31617	1,000	-1,0535	1,1535
		CONTROL_Day6	,55000	,31617	,838	-,5535	1,6535
		G_GI_CT@MT10_Day0	-1,45000*	,31617	,003	-2,5535	-,3465
		G_GI_CT@MT10_Day2	-1,07500	,31617	,062	-2,1785	,0285
		G_GI_CT@MT10_Day4	-,52500	,31617	,874	-1,6285	,5785
		G_GI_EG@MT15_Day0	-1,45000*	,31617	,003	-2,5535	-,3465
		G_GI_EG@MT15_Day2	-1,32500*	,31617	,008	-2,4285	-,2215
		G_GI_EG@MT15_Day4	-,90000	,31617	,204	-2,0035	,2035
		G_GI_EG@MT15_Day6	-,52500	,31617	,874	-1,6285	,5785
	G_GI_EG@MT15_Day0	CONTROL_Day0	,00000	,31617	1,000	-1,1035	1,1035
		CONTROL_Day2	,82500	,31617	,312	-,2785	1,9285
		CONTROL_Day4	1,50000*	,31617	,002	,3965	2,6035
		CONTROL_Day6	2,00000*	,31617	<,001	,8965	3,1035

		G_GI_CT@MT10_Day0	,00000	,31617	1,000	-1,1035	1,1035
		G_GI_CT@MT10_Day2	,37500	,31617	,987	-,7285	1,4785
		G_GI_CT@MT10_Day4	,92500	,31617	,175	-,1785	2,0285
		G_GI_CT@MT10_Day6	1,45000*	,31617	,003	,3465	2,5535
		G_GI_EG@MT15_Day2	,12500	,31617	1,000	-,9785	1,2285
		G_GI_EG@MT15_Day4	,55000	,31617	,838	-,5535	1,6535
		G_GI_EG@MT15_Day6	,92500	,31617	,175	-,1785	2,0285
	G_GI_EG@MT15_Day2	CONTROL_Day0	-,12500	,31617	1,000	-1,2285	,9785
		CONTROL_Day2	,70000	,31617	,551	-,4035	1,8035
		CONTROL_Day4	1,37500*	,31617	,005	,2715	2,4785
		CONTROL_Day6	1,87500*	,31617	<,001	,7715	2,9785
		G_GI_CT@MT10_Day0	-,12500	,31617	1,000	-1,2285	,9785
		G_GI_CT@MT10_Day2	,25000	,31617	1,000	-,8535	1,3535
		G_GI_CT@MT10_Day4	,80000	,31617	,355	-,3035	1,9035
		G_GI_CT@MT10_Day6	1,32500*	,31617	,008	,2215	2,4285
		G_GI_EG@MT15_Day0	-,12500	,31617	1,000	-1,2285	,9785
		G_GI_EG@MT15_Day4	,42500	,31617	,967	-,6785	1,5285
		G_GI_EG@MT15_Day6	,80000	,31617	,355	-,3035	1,9035
	G_GI_EG@MT15_Day4	CONTROL_Day0	-,55000	,31617	,838	-1,6535	,5535
		CONTROL_Day2	,27500	,31617	,999	-,8285	1,3785
		CONTROL_Day4	,95000	,31617	,149	-,1535	2,0535
		CONTROL_Day6	1,45000*	,31617	,003	,3465	2,5535
		G_GI_CT@MT10_Day0	-,55000	,31617	,838	-1,6535	,5535
		G_GI_CT@MT10_Day2	-,17500	,31617	1,000	-1,2785	,9285
		G_GI_CT@MT10_Day4	,37500	,31617	,987	-,7285	1,4785
		G_GI_CT@MT10_Day6	,90000	,31617	,204	-,2035	2,0035
		G_GI_EG@MT15_Day0	-,55000	,31617	,838	-1,6535	,5535
		G_GI_EG@MT15_Day2	-,42500	,31617	,967	-1,5285	,6785
		G_GI_EG@MT15_Day6	,37500	,31617	,987	-,7285	1,4785
	G_GI_EG@MT15_Day6	CONTROL_Day0	-,92500	,31617	,175	-2,0285	,1785
		CONTROL_Day2	-,10000	,31617	1,000	-1,2035	1,0035
		CONTROL_Day4	,57500	,31617	,798	-,5285	1,6785

		CONTROL_Day6	1,07500	,31617	,062	-,0285	2,1785
		G_GI_CT@MT10_Day0	-,92500	,31617	,175	-2,0285	,1785
		G_GI_CT@MT10_Day2	-,55000	,31617	,838	-1,6535	,5535
		G_GI_CT@MT10_Day4	,00000	,31617	1,000	-1,1035	1,1035
		G_GI_CT@MT10_Day6	,52500	,31617	,874	-,5785	1,6285
		G_GI_EG@MT15_Day0	-,92500	,31617	,175	-2,0285	,1785
		G_GI_EG@MT15_Day2	-,80000	,31617	,355	-1,9035	,3035
		G_GI_EG@MT15_Day4	-,37500	,31617	,987	-1,4785	,7285
Taste	CONTROL_Day0	CONTROL_Day2	,30000	,25894	,989	-,6038	1,2038
		CONTROL_Day4	1,02500*	,25894	,015	,1212	1,9288
		CONTROL_Day6	1,85000*	,25894	<,001	,9462	2,7538
		G_GI_CT@MT10_Day0	,00000	,25894	1,000	-,9038	,9038
		G_GI_CT@MT10_Day2	,25000	,25894	,998	-,6538	1,1538
		G_GI_CT@MT10_Day4	,95000*	,25894	,032	,0462	1,8538
		G_GI_CT@MT10_Day6	1,57500*	,25894	<,001	,6712	2,4788
		G_GI_EG@MT15_Day0	,00000	,25894	1,000	-,9038	,9038
		G_GI_EG@MT15_Day2	,32500	,25894	,980	-,5788	1,2288
		G_GI_EG@MT15_Day4	,77500	,25894	,152	-,1288	1,6788
		G_GI_EG@MT15_Day6	1,12500*	,25894	,005	,2212	2,0288
	CONTROL_Day2	CONTROL_Day0	-,30000	,25894	,989	-1,2038	,6038
		CONTROL_Day4	,72500	,25894	,222	-,1788	1,6288
		CONTROL_Day6	1,55000*	,25894	<,001	,6462	2,4538
		G_GI_CT@MT10_Day0	-,30000	,25894	,989	-1,2038	,6038
		G_GI_CT@MT10_Day2	-,05000	,25894	1,000	-,9538	,8538
		G_GI_CT@MT10_Day4	,65000	,25894	,366	-,2538	1,5538
		G_GI_CT@MT10_Day6	1,27500*	,25894	,001	,3712	2,1788
		G_GI_EG@MT15_Day0	-,30000	,25894	,989	-1,2038	,6038
		G_GI_EG@MT15_Day2	,02500	,25894	1,000	-,8788	,9288
		G_GI_EG@MT15_Day4	,47500	,25894	,789	-,4288	1,3788
		G_GI_EG@MT15_Day6	,82500	,25894	,101	-,0788	1,7288
	CONTROL_Day4	CONTROL_Day0	-1,02500*	,25894	,015	-1,9288	-,1212
		CONTROL_Day2	-,72500	,25894	,222	-1,6288	,1788

		CONTROL_Day6	,82500	,25894	,101	-,0788	1,7288
		G_GI_CT@MT10_Day0	-1,02500*	,25894	,015	-1,9288	-,1212
		G_GI_CT@MT10_Day2	-,77500	,25894	,152	-1,6788	,1288
		G_GI_CT@MT10_Day4	-,07500	,25894	1,000	-,9788	,8288
		G_GI_CT@MT10_Day6	,55000	,25894	,610	-,3538	1,4538
		G_GI_EG@MT15_Day0	-1,02500*	,25894	,015	-1,9288	-,1212
		G_GI_EG@MT15_Day2	-,70000	,25894	,265	-1,6038	,2038
		G_GI_EG@MT15_Day4	-,25000	,25894	,998	-1,1538	,6538
		G_GI_EG@MT15_Day6	,10000	,25894	1,000	-,8038	1,0038
	CONTROL_Day6	CONTROL_Day0	-1,85000*	,25894	<,001	-2,7538	-,9462
		CONTROL_Day2	-1,55000*	,25894	<,001	-2,4538	-,6462
		CONTROL_Day4	-,82500	,25894	,101	-1,7288	,0788
		G_GI_CT@MT10_Day0	-1,85000*	,25894	<,001	-2,7538	-,9462
		G_GI_CT@MT10_Day2	-1,60000*	,25894	<,001	-2,5038	-,6962
		G_GI_CT@MT10_Day4	-,90000	,25894	,052	-1,8038	,0038
		G_GI_CT@MT10_Day6	-,27500	,25894	,995	-1,1788	,6288
		G_GI_EG@MT15_Day0	-1,85000*	,25894	<,001	-2,7538	-,9462
		G_GI_EG@MT15_Day2	-1,52500*	,25894	<,001	-2,4288	-,6212
		G_GI_EG@MT15_Day4	-1,07500*	,25894	,009	-1,9788	-,1712
		G_GI_EG@MT15_Day6	-,72500	,25894	,222	-1,6288	,1788
	G_GI_CT@MT10_Day0	CONTROL_Day0	,00000	,25894	1,000	-,9038	,9038
		CONTROL_Day2	,30000	,25894	,989	-,6038	1,2038
		CONTROL_Day4	1,02500*	,25894	,015	,1212	1,9288
		CONTROL_Day6	1,85000*	,25894	<,001	,9462	2,7538
		G_GI_CT@MT10_Day2	,25000	,25894	,998	-,6538	1,1538
		G_GI_CT@MT10_Day4	,95000*	,25894	,032	,0462	1,8538
		G_GI_CT@MT10_Day6	1,57500*	,25894	<,001	,6712	2,4788
		G_GI_EG@MT15_Day0	,00000	,25894	1,000	-,9038	,9038
		G_GI_EG@MT15_Day2	,32500	,25894	,980	-,5788	1,2288
		G_GI_EG@MT15_Day4	,77500	,25894	,152	-,1288	1,6788
		G_GI_EG@MT15_Day6	1,12500*	,25894	,005	,2212	2,0288
	G_GI_CT@MT10_Day2	CONTROL_Day0	-,25000	,25894	,998	-1,1538	,6538

		CONTROL_Day2	,05000	,25894	1,000	-,8538	,9538
		CONTROL_Day4	,77500	,25894	,152	-,1288	1,6788
		CONTROL_Day6	1,60000*	,25894	<,001	,6962	2,5038
		G_GI_CT@MT10_Day0	-,25000	,25894	,998	-1,1538	,6538
		G_GI_CT@MT10_Day4	,70000	,25894	,265	-,2038	1,6038
		G_GI_CT@MT10_Day6	1,32500*	,25894	<,001	,4212	2,2288
		G_GI_EG@MT15_Day0	-,25000	,25894	,998	-1,1538	,6538
		G_GI_EG@MT15_Day2	,07500	,25894	1,000	-,8288	,9788
		G_GI_EG@MT15_Day4	,52500	,25894	,673	-,3788	1,4288
		G_GI_EG@MT15_Day6	,87500	,25894	,065	-,0288	1,7788
	G_GI_CT@MT10_Day4	CONTROL_Day0	-,95000*	,25894	,032	-1,8538	-,0462
		CONTROL_Day2	-,65000	,25894	,366	-1,5538	,2538
		CONTROL_Day4	,07500	,25894	1,000	-,8288	,9788
		CONTROL_Day6	,90000	,25894	,052	-,0038	1,8038
		G_GI_CT@MT10_Day0	-,95000*	,25894	,032	-1,8538	-,0462
		G_GI_CT@MT10_Day2	-,70000	,25894	,265	-1,6038	,2038
		G_GI_CT@MT10_Day6	,62500	,25894	,423	-,2788	1,5288
		G_GI_EG@MT15_Day0	-,95000*	,25894	,032	-1,8538	-,0462
		G_GI_EG@MT15_Day2	-,62500	,25894	,423	-1,5288	,2788
		G_GI_EG@MT15_Day4	-,17500	,25894	1,000	-1,0788	,7288
		G_GI_EG@MT15_Day6	,17500	,25894	1,000	-,7288	1,0788
	G_GI_CT@MT10_Day6	CONTROL_Day0	-1,57500*	,25894	<,001	-2,4788	-,6712
		CONTROL_Day2	-1,27500*	,25894	,001	-2,1788	-,3712
		CONTROL_Day4	-,55000	,25894	,610	-1,4538	,3538
		CONTROL_Day6	,27500	,25894	,995	-,6288	1,1788
		G_GI_CT@MT10_Day0	-1,57500*	,25894	<,001	-2,4788	-,6712
		G_GI_CT@MT10_Day2	-1,32500*	,25894	<,001	-2,2288	-,4212
		G_GI_CT@MT10_Day4	-,62500	,25894	,423	-1,5288	,2788
		G_GI_EG@MT15_Day0	-1,57500*	,25894	<,001	-2,4788	-,6712
		G_GI_EG@MT15_Day2	-1,25000*	,25894	,001	-2,1538	-,3462
		G_GI_EG@MT15_Day4	-,80000	,25894	,125	-1,7038	,1038
		G_GI_EG@MT15_Day6	-,45000	,25894	,839	-1,3538	,4538

G_GI_EG@MT15_Day0	CONTROL_Day0	,00000	,25894	1,000	-,9038	,9038
	CONTROL_Day2	,30000	,25894	,989	-,6038	1,2038
	CONTROL_Day4	1,02500*	,25894	,015	,1212	1,9288
	CONTROL_Day6	1,85000*	,25894	<,001	,9462	2,7538
	G_GI_CT@MT10_Day0	,00000	,25894	1,000	-,9038	,9038
	G_GI_CT@MT10_Day2	,25000	,25894	,998	-,6538	1,1538
	G_GI_CT@MT10_Day4	,95000*	,25894	,032	,0462	1,8538
	G_GI_CT@MT10_Day6	1,57500*	,25894	<,001	,6712	2,4788
	G_GI_EG@MT15_Day2	,32500	,25894	,980	-,5788	1,2288
	G_GI_EG@MT15_Day4	,77500	,25894	,152	-,1288	1,6788
	G_GI_EG@MT15_Day6	1,12500*	,25894	,005	,2212	2,0288
G_GI_EG@MT15_Day2	CONTROL_Day0	-,32500	,25894	,980	-1,2288	,5788
	CONTROL_Day2	-,02500	,25894	1,000	-,9288	,8788
	CONTROL_Day4	,70000	,25894	,265	-,2038	1,6038
	CONTROL_Day6	1,52500*	,25894	<,001	,6212	2,4288
	G_GI_CT@MT10_Day0	-,32500	,25894	,980	-1,2288	,5788
	G_GI_CT@MT10_Day2	-,07500	,25894	1,000	-,9788	,8288
	G_GI_CT@MT10_Day4	,62500	,25894	,423	-,2788	1,5288
	G_GI_CT@MT10_Day6	1,25000*	,25894	,001	,3462	2,1538
	G_GI_EG@MT15_Day0	-,32500	,25894	,980	-1,2288	,5788
	G_GI_EG@MT15_Day4	,45000	,25894	,839	-,4538	1,3538
	G_GI_EG@MT15_Day6	,80000	,25894	,125	-,1038	1,7038
G_GI_EG@MT15_Day4	CONTROL_Day0	-,77500	,25894	,152	-1,6788	,1288
	CONTROL_Day2	-,47500	,25894	,789	-1,3788	,4288
	CONTROL_Day4	,25000	,25894	,998	-,6538	1,1538
	CONTROL_Day6	1,07500*	,25894	,009	,1712	1,9788
	G_GI_CT@MT10_Day0	-,77500	,25894	,152	-1,6788	,1288
	G_GI_CT@MT10_Day2	-,52500	,25894	,673	-1,4288	,3788
	G_GI_CT@MT10_Day4	,17500	,25894	1,000	-,7288	1,0788
	G_GI_CT@MT10_Day6	,80000	,25894	,125	-,1038	1,7038
	G_GI_EG@MT15_Day0	-,77500	,25894	,152	-1,6788	,1288
	G_GI_EG@MT15_Day2	-,45000	,25894	,839	-1,3538	,4538

		G_GI_EG@MT15_Day6	,35000	,25894	,965	-,5538	1,2538
	G_GI_EG@MT15_Day6	CONTROL_Day0	-1,12500*	,25894	,005	-2,0288	-,2212
		CONTROL_Day2	-,82500	,25894	,101	-1,7288	,0788
		CONTROL_Day4	-,10000	,25894	1,000	-1,0038	,8038
		CONTROL_Day6	,72500	,25894	,222	-,1788	1,6288
		G_GI_CT@MT10_Day0	-1,12500*	,25894	,005	-2,0288	-,2212
		G_GI_CT@MT10_Day2	-,87500	,25894	,065	-1,7788	,0288
		G_GI_CT@MT10_Day4	-,17500	,25894	1,000	-1,0788	,7288
		G_GI_CT@MT10_Day6	,45000	,25894	,839	-,4538	1,3538
		G_GI_EG@MT15_Day0	-1,12500*	,25894	,005	-2,0288	-,2212
		G_GI_EG@MT15_Day2	-,80000	,25894	,125	-1,7038	,1038
		G_GI_EG@MT15_Day4	-,35000	,25894	,965	-1,2538	,5538

*. The mean difference is significant at the 0.05 level.

Kinetic Statistics

Descriptives

	graph_code			Statistic	Std. Error
k	a	Mean		,000360867000	,0000153246797
		95% Confidence Interval for Mean	Lower Bound	,000294930225	
			Upper Bound	,000426803775	
		5% Trimmed Mean		.	
		Median		,000365531000	
		Variance		,000	
		Std. Deviation		,0000265431239	
		Minimum		,0003323010	
		Maximum		,0003847690	
		Range		,0000524680	
		Interquartile Range		.	
		Skewness		-,766	1,225
		Kurtosis		.	.
	b	Mean		,004086666667	,0001752458590

		95% Confidence Interval for Mean	Lower Bound	,003332644593	
			Upper Bound	,004840688740	
		5% Trimmed Mean		.	
		Median		,004140000000	
		Variance		,000	
		Std. Deviation		,0003035347317	
		Minimum		,0037600000	
		Maximum		,0043600000	
		Range		,0006000000	
		Interquartile Range		.	
		Skewness		-,766	1,225
		Kurtosis		.	.
	c	Mean		,001114630000	,0001397320182
		95% Confidence Interval for Mean	Lower Bound	,000513411651	
			Upper Bound	,001715848349	
		5% Trimmed Mean		.	
		Median		,001190000000	
		Variance		,000	
		Std. Deviation		,0002420229549	
		Minimum		,0008438900	
		Maximum		,0013100000	
		Range		,0004661100	
		Interquartile Range		.	
		Skewness		-1,265	1,225
		Kurtosis		.	.
	d	Mean		,005786666667	,0010690234381
		95% Confidence Interval for Mean	Lower Bound	,001187030053	
			Upper Bound	,010386303281	
		5% Trimmed Mean		.	
		Median		,005310000000	
		Variance		,000	
		Std. Deviation		,0018516029092	
		Minimum		,0042200000	
		Maximum		,0078300000	
		Range		,0036100000	

		Interquartile Range		.	
		Skewness		1,082	1,225
		Kurtosis		.	.
	e	Mean		,001980000000	,0002532455988
		95% Confidence Interval for Mean	Lower Bound	,000890372133	
			Upper Bound	,003069627867	
		5% Trimmed Mean		.	
		Median		,002160000000	
		Variance		,000	
		Std. Deviation		,0004386342440	
		Minimum		,0014800000	
		Maximum		,0023000000	
		Range		,0008200000	
		Interquartile Range		.	
		Skewness		-1,536	1,225
		Kurtosis		.	.
	f	Mean		,005250000000	,0005307855813
		95% Confidence Interval for Mean	Lower Bound	,002966213970	
			Upper Bound	,007533786030	
		5% Trimmed Mean		.	
		Median		,004770000000	
		Variance		,000	
		Std. Deviation		,0009193475948	
		Minimum		,0046700000	
		Maximum		,0063100000	
		Range		,0016400000	
		Interquartile Range		.	
		Skewness		1,709	1,225
		Kurtosis		.	.
qe	a	Mean		,688690000000	,0298705323019
		95% Confidence Interval for Mean	Lower Bound	,560167472652	
			Upper Bound	,817212527348	
		5% Trimmed Mean		.	
		Median		,677400000000	
		Variance		,003	
		Std. Deviation		,0517372795961	

		Minimum		,6435300000	
		Maximum		,7451400000	
		Range		,1016100000	
		Interquartile Range		.	
		Skewness		,935	1,225
		Kurtosis		.	.
	b	Mean		,386983333333	,0167828824832
		95% Confidence Interval for Mean	Lower Bound	,314772418204	
			Upper Bound	,459194248463	
		5% Trimmed Mean		.	
		Median		,380640000000	
		Variance		,001	
		Std. Deviation		,0290688051583	
		Minimum		,3616100000	
		Maximum		,4187000000	
		Range		,0570900000	
		Interquartile Range		.	
		Skewness		,935	1,225
		Kurtosis		.	.
	c	Mean		,771566666667	,0220520764958
		95% Confidence Interval for Mean	Lower Bound	,676684239535	
			Upper Bound	,866449093798	
		5% Trimmed Mean		.	
		Median		,767530000000	
		Variance		,001	
		Std. Deviation		,0381953169032	
		Minimum		,7355500000	
		Maximum		,8116200000	
		Range		,0760700000	
		Interquartile Range		.	
		Skewness		,470	1,225
		Kurtosis		.	.
	d	Mean		,407226666667	,0170772766499
		95% Confidence Interval for Mean	Lower Bound	,333749075672	
			Upper Bound	,480704257661	
		5% Trimmed Mean		.	

		Median		,404900000000	
		Variance		,001	
		Std. Deviation		,0295787108126	
		Minimum		,3788800000	
		Maximum		,4379000000	
		Range		,0590200000	
		Interquartile Range		.	
		Skewness		,352	1,225
		Kurtosis		.	.
	e	Mean		,972630000000	,0424330888969
		95% Confidence Interval for Mean	Lower Bound	,790055154226	
			Upper Bound	1,155204845774	
		5% Trimmed Mean		.	
		Median		,931640000000	
		Variance		,005	
		Std. Deviation		,0734962658915	
		Minimum		,9287700000	
		Maximum		1,0574800000	
		Range		,1287100000	
		Interquartile Range		.	
		Skewness		1,729	1,225
		Kurtosis		.	.
	f	Mean		,513803333333	,0150227131741
		95% Confidence Interval for Mean	Lower Bound	,449165815487	
			Upper Bound	,578440851180	
		5% Trimmed Mean		.	
		Median		,523890000000	
		Variance		,001	
		Std. Deviation		,0260201024851	
		Minimum		,4842500000	
		Maximum		,5332700000	
		Range		,0490200000	
		Interquartile Range		.	
		Skewness		-1,482	1,225
		Kurtosis		.	.
R2	a	Mean		,948440000000	,000000000000

		95% Confidence Interval for Mean	Lower Bound	,948440000000	
			Upper Bound	,948440000000	
		5% Trimmed Mean		,948440000000	
		Median		,948440000000	
		Variance		,000	
		Std. Deviation		,0000000000000	
		Minimum		,9484400000	
		Maximum		,9484400000	
		Range		,0000000000	
		Interquartile Range		,0000000000	
		Skewness		.	.
		Kurtosis		.	.
	b	Mean		,801626666667	,0148333333333
		95% Confidence Interval for Mean	Lower Bound	,737803984509	
			Upper Bound	,865449348825	
		5% Trimmed Mean		.	
		Median		,816460000000	
		Variance		,001	
		Std. Deviation		,0256920869789	
		Minimum		,7719600000	
		Maximum		,8164600000	
		Range		,0445000000	
		Interquartile Range		.	
		Skewness		-1,732	1,225
		Kurtosis		.	.
	c	Mean		,952986666667	,0032948764941
		95% Confidence Interval for Mean	Lower Bound	,938809957325	
			Upper Bound	,967163376008	
		5% Trimmed Mean		.	
		Median		,952410000000	
		Variance		,000	
		Std. Deviation		,0057068934924	
		Minimum		,9475900000	
		Maximum		,9589600000	
		Range		,0113700000	

	Interquartile Range		.	
	Skewness		,450	1,225
	Kurtosis		.	.
d	Mean		,906123333333	,0094109799938
	95% Confidence Interval for Mean	Lower Bound	,865631154573	
		Upper Bound	,946615512093	
	5% Trimmed Mean		.	
	Median		,906010000000	
	Variance		,000	
	Std. Deviation		,0163002954983	
	Minimum		,8898800000	
	Maximum		,9224800000	
	Range		,0326000000	
	Interquartile Range		.	
	Skewness		,031	1,225
	Kurtosis		.	.
e	Mean		,982280000000	,0017967748885
	95% Confidence Interval for Mean	Lower Bound	,974549101621	
		Upper Bound	,990010898379	
	5% Trimmed Mean		.	
	Median		,983100000000	
	Variance		,000	
	Std. Deviation		,0031121053967	
	Minimum		,9788400000	
	Maximum		,9849000000	
	Range		,0060600000	
	Interquartile Range		.	
	Skewness		-1,103	1,225
	Kurtosis		.	.
f	Mean		,948620000000	,0109268156386
	95% Confidence Interval for Mean	Lower Bound	,901605706865	
		Upper Bound	,995634293135	
	5% Trimmed Mean		.	
	Median		,956670000000	
	Variance		,000	
	Std. Deviation		,0189257998510	

	Minimum	,9270000000	
	Maximum	,9621900000	
	Range	,0351900000	
	Interquartile Range	.	
	Skewness	-1,568	1,225
	Kurtosis	.	.

References

1. Saleh, T.A. Chapter 3 - Kinetic Models and Thermodynamics of Adsorption Processes: Classification. In *Interface Science and Technology*; Saleh, T.A., Ed.; Surface Science of Adsorbents and Nanoadsorbents; Elsevier, 2022; Vol. 34, pp. 65–97.
2. Asimakopoulos, G.; Baikousi, M.; Salmas, C.; Bourlinos, A.B.; Zboril, R.; Karakassides, M.A. Advanced Cr(VI) Sorption Properties of Activated Carbon Produced via Pyrolysis of the “<i>Posidonia Oceanica”</i> Seagrass. *J. Hazard. Mater.* **2021**, *405*, 124274, doi:10.1016/j.jhazmat.2020.124274.
3. Frenkel, J. Theorie der Adsorption und verwandter Erscheinungen. *Z. Für Phys.* **1924**, *26*, 117–138, doi:10.1007/BF01327320.
4. Knopf, D.A.; Ammann, M. Technical Note: Adsorption and Desorption Equilibria from Statistical Thermodynamics and Rates from Transition State Theory. *Atmospheric Chem. Phys.* **2021**, *21*, 15725–15753, doi:10.5194/acp-21-15725-2021.
5. Arrhenius, S. Über die Dissociationswärme und den Einfluss der Temperatur auf den Dissociationsgrad der Elektrolyte. *Z. Für Phys. Chem.* **1889**, *4U*, 96–116, doi:10.1515/zpch-1889-0408.
6. Kechagias, A.; Salmas, C.E.; Chalmpes, N.; Leontiou, A.A.; Karakassides, M.A.; Giannelis, E.P.; Giannakas, A.E. Laponite vs. Montmorillonite as Eugenol Nanocarriers for Low Density Polyethylene Active Packaging Films. *Nanomaterials* **2024**, *14*, 1938, doi:10.3390/nano14231938.

7. Salmas, C.E.; Giannakas, A.E.; Baikousi, M.; Kollia, E.; Tsigkou, V.; Proestos, C. Effect of Copper and Titanium-Exchanged Montmorillonite Nanostructures on the Packaging Performance of Chitosan/Poly-Vinyl-Alcohol-Based Active Packaging Nanocomposite Films. *Foods* **2021**, *10*, 3038, doi:10.3390/foods10123038.
8. Karabagias, I.K.; Karabagias, V.K.; Badeka, A.V. In Search of the EC60: The Case Study of Bee Pollen, Quercus Ilex Honey, and Saffron. *Eur. Food Res. Technol.* **2020**, *246*, 2451–2459, doi:10.1007/s00217-020-03588-8.
9. Connolly, J.M.; Kane, M.T.; Quinlan, L.R.; Hynes, A.C. Enhancing Oxygen Delivery to Ovarian Follicles by Three Different Methods Markedly Improves Growth in Serum-Containing Culture Medium. *Reprod. Fertil. Dev.* **2019**, *31*, 1339–1352, doi:10.1071/RD18286.
10. Stratakos, A.C.; Koidis, A. Chapter 4 - Methods for Extracting Essential Oils. In *Essential Oils in Food Preservation, Flavor and Safety*; Preedy, V.R., Ed.; Academic Press: San Diego, 2016; pp. 31–38 ISBN 978-0-12-416641-7.
11. Turalija, M.; Bischof, S.; Budimir, A.; Gaan, S. Antimicrobial PLA Films from Environment Friendly Additives. *Compos. Part B Eng.* **2016**, *102*, 94–99, doi:10.1016/j.compositesb.2016.07.017.
12. Ardjoum, N.; Chibani, N.; Shankar, S.; Fadhel, Y.B.; Djidjelli, H.; Lacroix, M. Development of Antimicrobial Films Based on Poly(Lactic Acid) Incorporated with *Thymus Vulgaris* Essential Oil and Ethanolic Extract of Mediterranean Propolis. *Int. J. Biol. Macromol.* **2021**, *185*, 535–542, doi:10.1016/j.ijbiomac.2021.06.194.
13. Zaharioudakis, K.; Salmas, C.E.; Andritsos, N.D.; Kollia, E.; Leontiou, A.; Karabagias, V.K.; Karydis-Messinis, A.; Moschovas, D.; Zafeiropoulos, N.E.; Avgeropoulos, A.; et al. Carvacrol, Citral, Eugenol and Cinnamaldehyde Casein Based Edible Nanoemulsions as Novel Sustainable Active Coatings for Fresh Pork Tenderloin Meat Preservation. *Front. Food Sci. Technol.* **2024**, *4*, doi:10.3389/frfst.2024.1400224.
